# A Highly Active and Selective Zirconium-Based Catalyst System for the Industrial Production of Poly(lactic acid)

**DOI:** 10.1021/acscatal.2c05690

**Published:** 2023-02-07

**Authors:** Antoine Buchard, Christopher J. Chuck, Matthew G. Davidson, Gerrit Gobius du Sart, Matthew D Jones, Strachan N. McCormick, Andrew D. Russell

**Affiliations:** † Institute for Sustainability, University of Bath, BathBA2 7AY, U.K.; ‡ Department of Chemistry, University of Bath, BathBA2 7AY, U.K.; § Department of Chemical Engineering, University of Bath, BathBA2 7AY, U.K.; ∥ TotalEnergies Corbion, Arkelsedijk 46, 4206 ACGorinchem, The Netherlands

**Keywords:** lactide, ring-opening polymerization, poly(lactic acid), zirconium, amine tris(phenolate), industrially relevant, melt polymerization, liquid catalyst formulation

## Abstract

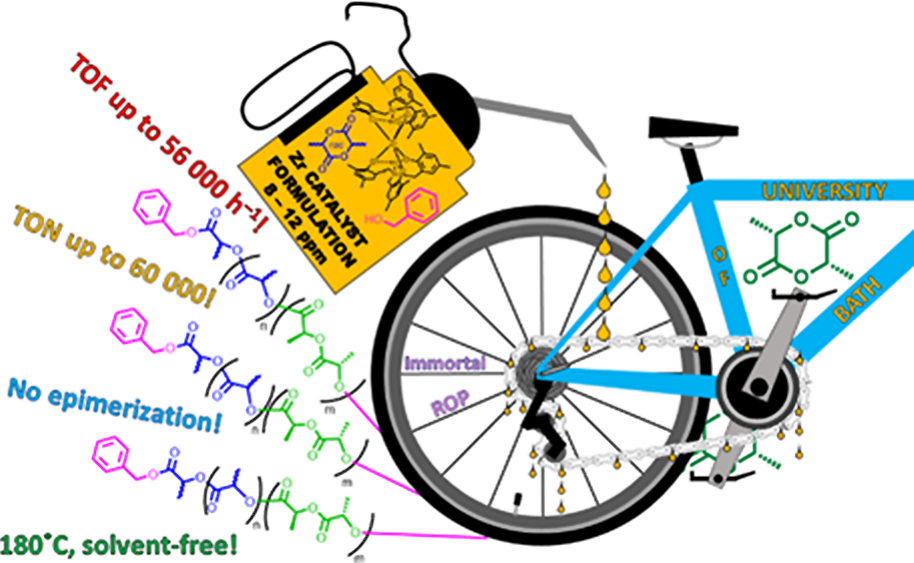

The biodegradable, aliphatic polyester poly(lactic acid), PLA, is a leading bio-based alternative to petrochemical-derived plastic materials across a range of applications. Widely reported in the available literature as a benchmark for PLA production *via* the bulk ring-opening polymerization of lactides is the use of divalent tin catalysts, and particularly tin(II) bis(2-ethylhexanoate). We present an alternative zirconium-based system that combines an inexpensive Group IV metal with the robustness, high activity, control, and designed compatibility with existing facilities and processes, that are required for industrial use. We have carried out a comprehensive kinetic study and applied a combined experimental and theoretical approach to understanding the mechanism by which the polymerization of lactide proceeds in the presence of this system. In the laboratory-scale (20 g) polymerization of recrystallized racemic d,l-lactide (*rac*-lactide), we have measured catalyst turnover frequencies up to at least 56,000 h^–1^, and confirmed the reported protocols’ resistance toward undesirable epimerization, transesterification, and chain scission processes, deleterious to the properties of the polymer product. Further optimization and scale-up under industrial conditions have confirmed the relevance of the catalytic protocol to the commercial production of melt-polymerized PLA. We were able to undertake the efficient preparation of high-molecular-weight PLA on the 500–2000 g scale, *via* the selective and well-controlled polymerization of commercial polymer-grade l-lactide under challenging, industrially relevant conditions, and at metal concentrations as low as 8–12 ppm Zr by weight ([Zr] = 1.3 × 10^–3^ to 1.9 × 10^–3^ mol %). Under those conditions, a catalyst turnover number of at least 60,000 was attained, and the activity of the catalyst was comparable to that of tin(II) bis(2-ethylhexanoate).

## Introduction

Since beginning in the 1950s, commercial commodity plastics production has seen enormous expansion, now annually exceeding 400 million tons, almost all of which is of nonrenewable, petrochemical origin.^1^ The diverse thermal and mechanical properties, robustness, and stability of many plastics have ensured remarkable proliferation across innumerable applications. In particular, plastics dominate in the packaging sector, with little of the associated material undergoing closed-loop recycling.^1^ Accordingly, plastics’ extreme longevity has caused extensive contamination throughout Earth’s ecosystems, an estimated 5000 billion kg having entered landfills or the environment to-date, with a projected increase to 12,000 billion kg by 2050.^2^ Provision of bio-derived and biodegradable alternatives is an urgent necessity.

**Scheme 1:**
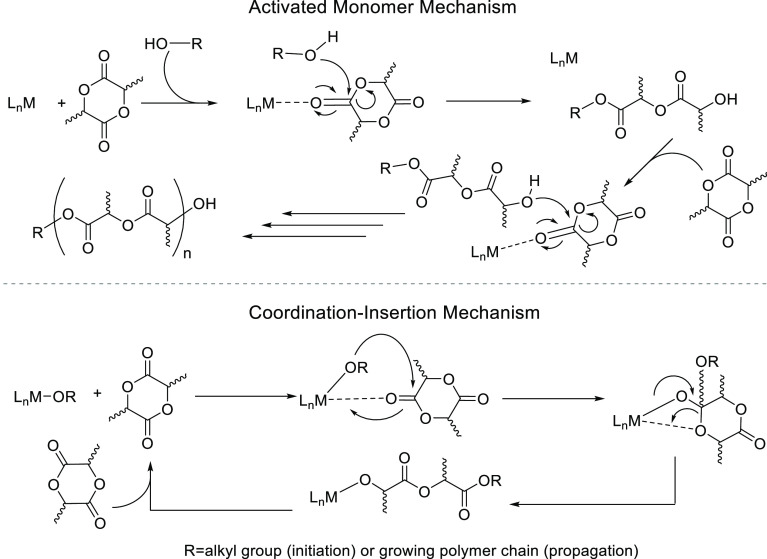
General Mechanistic Pathways for the Activated Monomer- (Lewis Acid-Catalyzed) and Coordination–Insertion-Type ROP of Lactide in the Presence of a Metal-Based Catalyst, L_*n*_M or L_*n*_MOR, Respectively^3−11^

The most commercially viable bio-based thermoplastic is poly(lactic acid), PLA, typically prepared *via* the controlled catalytic ring-opening polymerization (ROP) of lactide (LA), allowing efficient production of a high-molecular-weight polymer.^12^ Typically, for commercial production, the metal-catalyzed ROP of lactones and lactides proceeds *via* a coordination–insertion mechanism.^3−9^ An alternative, activated monomer, pathway for the ROP of LA (Scheme 1) has also been reported,^7,10,11^ this invariably affording immortal kinetics. In a variation of this latter mechanism, Carpentier and co-workers have described rare earth aminoether-phenolate catalysts for the solution-state ROP of l-lactide (l-LA) that favor a “ligand-assisted activated monomer” (LAAM) mechanism, in which activation of the alcohol co-initiator by the anionic phenolate occurs in tandem with activation of the monomer at the Lewis-acidic metal center.^13^ Wu and co-workers have reported highly isoselective alkali metal monophenolate systems for the ROP of racemic d,l-lactide (*rac*-LA), proceeding *via* a similar pathway.^14−16^ Although this work focuses on metal-based ROP protocols and associated mechanisms, diverse organocatalytic systems have also been reported.^4,17−20^

Scalable syntheses of PLA are conducted at elevated temperatures (140–200 °C) under solvent-free (melt) conditions,^21^ with tin(II) bis(2-ethylhexanoate), Sn(Oct)_2_, generally accepted as the benchmark precatalyst.^4,7,9,12,20,22−26^ Sn(Oct)_2_ is inexpensive, is highly active at low loadings,^27^ and induces minimal epimerization of stereopure monomers under the high-temperature, solvent-free conditions required at scale.

Sn(Oct)_2_ affords immortal ROP kinetics,^28−32^ such that addition of an exogenous alcohol nucleophile (chain-transfer agent, co-initiator) can be used to control the polymer molecular weight independently of the Sn(Oct)_2_ concentration. Sn(Oct)_2_ is also a liquid, reasonably tolerant toward ambient atmospheric conditions,^33^ ensuring easy storage and manipulation. Mechanistic studies of Sn(Oct)_2_-mediated ROP have pointed to initiation being predicated upon the equilibrium formation of a Sn-alcohol or Sn alkoxide complex on reaction of the precatalyst and nucleophile. This proceeds initially *via* partial abstraction of the acidic proton of the alcohol by the retained octanoate ligand, somewhat reminiscent of alcohol activation in the LAAM mechanism, followed by complete protonation occurring in tandem with coordination of the monomer.^8,34−36^

Development of alternatives to Sn(Oct)_2_, compatible with existing infrastructure, is a significant and enduring challenge. Consequently, the application of benign and earth-abundant metals to the catalytic ROP of LA has received much research attention over several decades. Nonetheless, catalyst performance and compatibility with challenging conditions have often been lacking.

Robust, air- and moisture-tolerant initiators for use under solvent-free conditions, such as several of Herres-Pawlis and co-workers’ zinc species,^37−50^ and aluminum systems prepared by Feijen and others,^51,52^ have typically afforded only modest activities. Nonetheless, several Al-catalyzed protocols have exhibited stereoselectivity under solvent-free conditions, including Feijen’s system,^51^ and others reported by Nomura^53,54^ and by Paetz.^21^ However, the high catalyst loadings at which use of these systems has typically been reported ([Al] > 0.3 mol %) and associated shortcomings, variously, in robustness, activity, and selectivity, severely limit their potential relevance to the commercial production of high-performance PLA.

Dysprosium- and yttrium-based protocols reported by the groups of Wu and Lin, while extremely robust, have similarly suffered from drawbacks including low activity, poor control, and non-immortal kinetics, alongside the problem of metal scarcity.^33,55^ Mehrkhodovandi and co-workers developed an immortal indium salan initiator, suitable for use in ambient air, affording high activity and good control at 120 °C ([In] = 1.0 × 10^–2^ mol %, 120 min, > 70% conversion, *M*_n_ = 101,000). However, In is neither biochemically benign, nor abundant or inexpensive, and 120 °C is far below the temperature range used industrially for the bulk ROP of LA.

Several more recent protocols have somewhat overcome these limitations. Pellecchia and co-workers described monophenolate-supported Zn amide complexes, comparable in activity to Sn(Oct)_2_ in polymerizing unpurified technical-grade LA at 190 °C (TOF = 3 × 10^4^ h^–1^).^56^ Similarly, Herres-Pawlis and co-workers have achieved rates significantly surpassing Sn(Oct)_2_ at 150 °C with both a Zn bisguanidine initiator^57^ and a Zn germylene system,^58^ although neither was reported to be sufficiently robust for use at scale. Most notably, however, the same group’s Fe guanidine system offered activity significantly surpassing that of Sn(Oct)_2_ in the ROP of unpurified technical LA, at low metal loadings ([Fe] < 2 × 10^–2^ mol %).^59^

Protocols employing the benign, earth-abundant Group 4 elements, titanium and zirconium, have received attention from our group and others.^5,60−75^ The resulting reports have included highly water-tolerant (but non-immortal) Ti and Zr Schiff base complexes^71^ and robust, albeit poorly controlled, *N*,*N*,*N*′,*N*′-tetrakis(2-hydroxyethyl)ethylenediamino complexes of the same metals.^75^

We have previously prepared highly active and heteroselective amine tris(phenolate)-supported Zr and Hf isopropoxides for the solvent-free ROP of *rac*-LA (Zr, *P*_r_ = 0.96), proceeding *via* a coordination–insertion mechanism, wherein stereocontrol was attributed to a dynamic enantiomorphic site.^61,69^ PLA produced with the Zr species was shown *in vitro* to be well-suited to biomedical applications,^73^ but both complexes’ air- and moisture-sensitivity precludes industrial adoption.^76^ More recently, Kol and co-workers have described a Zr(IV) isopropoxide initiator supported by a bulky mesityl-substituted amine tris(phenolate) ligand exhibiting remarkable activity, reportedly outperforming Sn(Oct)_2_ at precatalyst loadings below [Zr] = 5.0 × 10^–4^ mol % in the immortal ROP of unpurified LA at 180 °C, and remaining active at [Zr] = 1.0 × 10^–4^ mol %.^77^

Herein, we report an industrially relevant, inexpensive, and synthetically straightforward zirconium amine tris(phenolate)-catalyzed protocol for the immortal ring-opening polymerization of lactides. The robust, air- and moisture-stable initiating system is highly active under challenging conditions, and ROP likely proceeds *via* a ligand-assisted activated monomer mechanism. A novel preparation method has yielded stable, storable, liquid catalyst formulations, ensuring compatibility with existing facilities, and producing well-controlled reaction kinetics, while adhering to the Principles of Green Chemistry.^78,79^

## Results and Discussion

### Preparation of Catalyst Formulation

**Figure 1:**
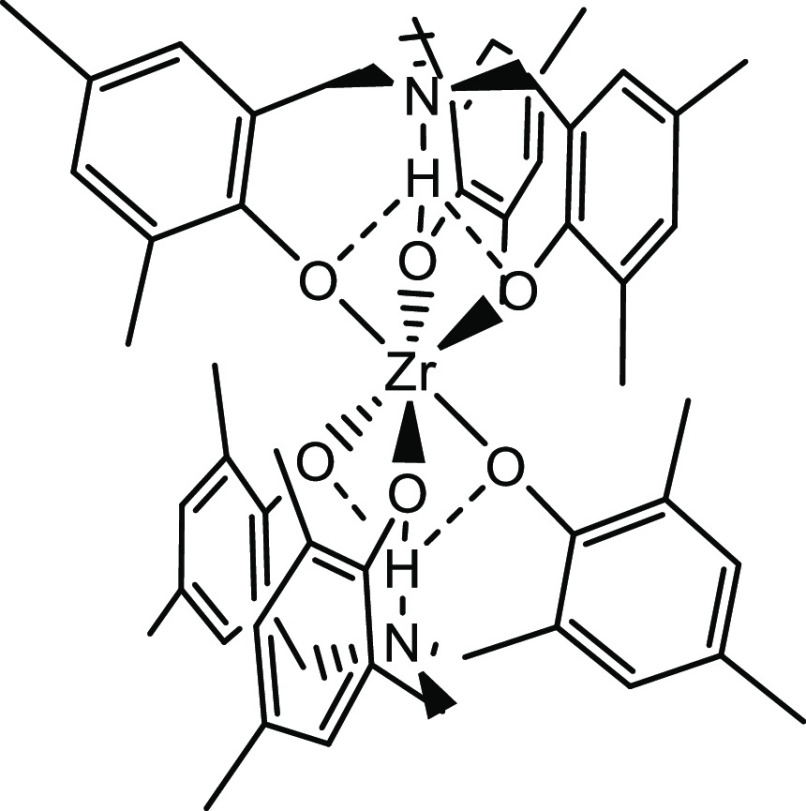
Zwitterionic zirconium amine tris(phenolate) complex Zr(HL^Me^)_2_, **1**.

We have previously reported the structure of colorless, air- and moisture-stable, zwitterionic zirconium amine tris(phenolate) complex Zr(HL^Me^)_2_, **1** (Figure 1).^80^

Initial screening experiments (not reported in detail here) revealed **1** to be highly active for the ROP of LA under solvent-free conditions and in the presence of a co-initiator. However, the solubility of solid complex **1** in industrially relevant (high-boiling, aprotic, low-toxicity) solvents was found to be very poor, limiting its potential for application as a bulk polymerization catalyst, able to afford the predictable reaction kinetics required for an industrial process. Solubility was not enhanced by modification of the ligand architecture (see the Supporting Information), necessitating development of a novel catalyst formulation method.

**Scheme 2:**
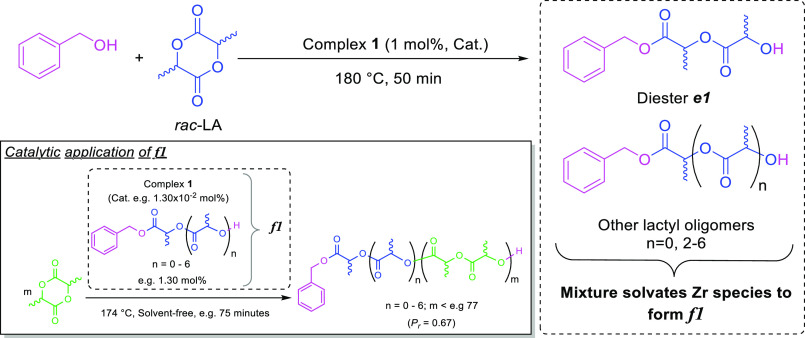
Preparation of Catalyst Formulation ***f1*** to Generate the Oligoester Solvent Phase and, Inset, Catalytic Application to the Immortal ROP of *rac*-LA

Heating **1**, *rac*-LA, and benzyl alcohol, BnOH, in the molar ratio 1:100:100, to 180 °C for 50 min under an inert atmosphere yielded a clear, yellow solution, denoted formulation ***f1***. After cooling to ambient temperature ***f1*** readily initiated ROP on addition to bulk molten LA and was amenable to long-term storage, with no precipitation of solid material observed after 18 months. Mass spectrometry and ^1^H and ^13^C{^1^H} NMR spectroscopic analyses revealed ***f1*** to be primarily composed of benzyl esters of oligomers comprising up to 7 lactate units, rather than simply the anticipated product of a single LA ring-opening event, diester ***e1*** (Scheme 2). Observation of a 72 g mol^–1^ repeat unit, derived from a single lactic acid molecule, was indicative of transesterification having occurred during formulation, rather than simply deviation from ideal immortal polymerization kinetics. The concentration of Zr in ***f1*** ([Zr]_***f1***_) was calculated to be 4.27 × 10^–2^ mol dm^–3^ (3.90 g L^–1^), the system containing 1 mol % Zr with respect to the number of alcohol groups present. Liquid formulations containing higher Zr concentrations than ***f1*** were not accessible using the current approach. Although ***f1*** was anticipated to be stable toward ambient atmospheric conditions, exposure to air was minimized to prevent contamination with protic impurities (moisture) which could reduce polymer molecular weight control in catalytic use.

In addition to ensuring compatibility with bulk polymerization conditions, use of ***f1*** for the ROP of LA eliminates any need for extraneous solvent and ensures rapid dispersion of the catalyst throughout the monomer melt. The *in situ*-generated solvent phase serves as the co-initiator, thus being incorporated into the product and fulfilling many of the Principles of Green Chemistry.^78,79^ Due to the large mass variation in the oligomeric co-initiating species, and the many variables associated with preparation and use of ***f1***, theoretical molecular weights for polymers prepared in the current work are given to the nearest 500 g mol^–1^. Although the intrinsic molar ratio of [ROH]:[Zr] = 100:1 in ***f1*** necessitates use of extremely low metal concentrations for the preparation of high-molecular-weight PLA, the high activity of the system ensures retention of industrial viability.

### Polymerization of LA and Kinetic Studies

**Figure 2:**
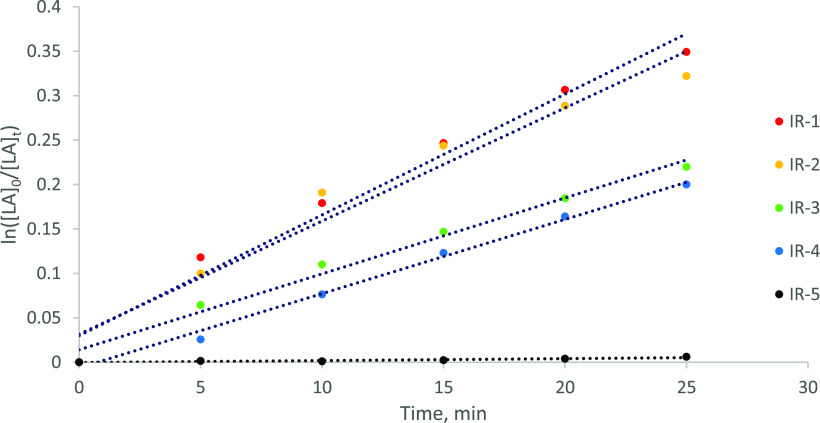
Semilogarithmic initial rate plots for determination of the effects of monomer stereochemistry and catalyst delivery method on the rate of the ROP of LA at [Zr] = 2.5 × 10^–3^ mol % ([LA]:[Zr]:[ROH] = 40,000:1:100; 16 ppm Zr by weight). Labels refer to entry numbers in Table 1. *R*^2^ values for IR-1–IR-5 = 0.98, 0.96, 0.99, 0.99, and 0.89, respectively.

Kinetic analyses of the solvent-free ROP of LA employing **1** and ***f1***, respectively, were undertaken *via in situ* ATR-FT-IR spectroscopic reaction monitoring, as described previously by our group and others (Table 1, Figure 2).^48,75,81,82^ Observed rate constants, *k*_obs_, were obtained from semilogarithmic plots of ln([LA]_0_/[LA]_*t*_) *versus* time, with the ROP being first-order with respect to LA. To minimize any solubility-related effects, initial comparison of the solid catalyst **1** and formulation ***f1*** for the ROP of both l-LA and *rac*-LA used a very low metal loading of [Zr] = 2.5 × 10^–3^ mol % ([LA]:[Zr]:[ROH] = 40,000:1:100; 16 ppm Zr by weight). For both monomers, **1** (with 100 equiv. BnOH) and ***f1*** afforded comparable rates, but *rac*-LA was consistently polymerized more quickly than l-LA.

**Table 1:** Polymerization Data for Comparison of Monomer Stereochemistry and Catalyst Delivery Methods^a^

*Entry*	*Monomer*	*Catalyst*	*Duration, min*	Conversion,^e^ %	*M*_*n*_^*Theo*^,^f^*g mol*^*–1*^	*M*_*n*_^*GPC*^*,*^g^*g mol^–1^*	*Đ*_*M*_^g^	*k*_*obs*_*,^h^ min*^*–1*^
*IR-1*^b^	*rac*-LA	***f1***	880	91	52500	29050	1.25	1.36 × 10^–2^
*IR-2*^c^	*rac*-LA	solid **1**	335	84	48500	39750	1.23	1.27 × 10^–2^
*IR-3*^b^	l-LA	***f1***	355	72	41500	16450	1.08	8.5 × 10^–3^
*IR-4*^c^	l-LA	solid **1**	470	72	41500	36650	1.19	8.4 × 10^–3^
*IR-5*^d^	*rac*-LA	none	315	4	N/A	N/A	N/A	2 × 10^–4^

a Conditions: 20 g of LA, solvent-free, 174 °C, [Zr] = 2.5 × 10^–3^ mol %, [ROH] = 0.25 mol % ([LA]:[Zr]:[ROH] = 40,000:1:100; 16 ppm Zr by weight).

b ***f1*** used.

c Solid **1** and BnOH dosed separately and undiluted.

d Control reaction; no catalyst, 0.25 mol % BnOH ([LA]:[Zr]:[ROH] = 40,000:0:100).

e Conversion determined *via*^1^H NMR spectroscopy, by integration of LA and PLA methine resonances.

f *M*_n_^Theo^ calculated from conversion and alcohol concentration: 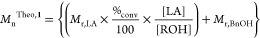

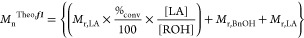

g Determined *via* GPC analysis in THF using Triple Detection.

h Determined by initial rate analysis *via in situ* ATR-FT-IR spectroscopic reaction monitoring.

The stereoselectivity of **1** and ***f1***, respectively, was determined both by (i) the relative rates of ROP of *rac*-LA and l-LA^53^ and (ii) homonuclear decoupled ^1^H NMR spectroscopic analysis to determine the polymer microstructure.^83^ The probability of racemic enchainment, *P*_r_ = 0.67, describing a slight heterotactic bias, was consistent for **1** and ***f1*** (Table 2), and there was excellent agreement between methods. Although the mechanistic basis of the stereoselectivity is unclear, these results suggest that **1** and ***f1*** share a common active species, and that stereochemical defects arising from epimerization and transesterification processes are negligible under the ROP conditions.

**Table 2:** Stereoselectivity Data for Catalysts ***f1*** and Solid **1**^a^

*Entry*	*Initiator*	*P*_*r*_^*NMR*^ b	*P*_*r*_^*k_obs_*^ c	*P*_*r*_^*Mean*^ d	*Tacticity*
*IR-1*	***f1***	0.65	0.69	0.67	heterotactically enriched
*IR-2*	solid **1** + BnOH	0.67	0.67	0.67	heterotactically enriched

a Details: [Zr] = 2.5 × 10^–3^ mol %, [ROH] = 0.25 mol % ROH ([LA]:[Zr]:[ROH] = 40,000:1:100; 16 ppm Zr by weight).

b *P*_r_ calculated *via* polymer microstructure analysis (^1^H{^1^H} NMR); *P*_r_ = √(2-[*sis*]).^83^

c *P*_r_ calculated *via* kinetic methods, *P*_r_ = 1 – (0.5(*k*_obs(_l-LA)/*k*_obs(*rac*-LA)_);^53^ for determination of *P*_r_ for IR-1, the value *k*_obs_(l-LA) was taken from IR-3, and for IR-2, the value *k*_obs_(l-LA) was taken from IR-4.

d Mean *P*_r_ calculated from both methods.

Homochiral poly(l-lactic acid), PlLA, is of greater industrial relevance than *rac*-LA-derived materials, and management of stereochemical defects is essential to control its crystallinity and thermomechanical properties.^1,8^

**Figure 3:**
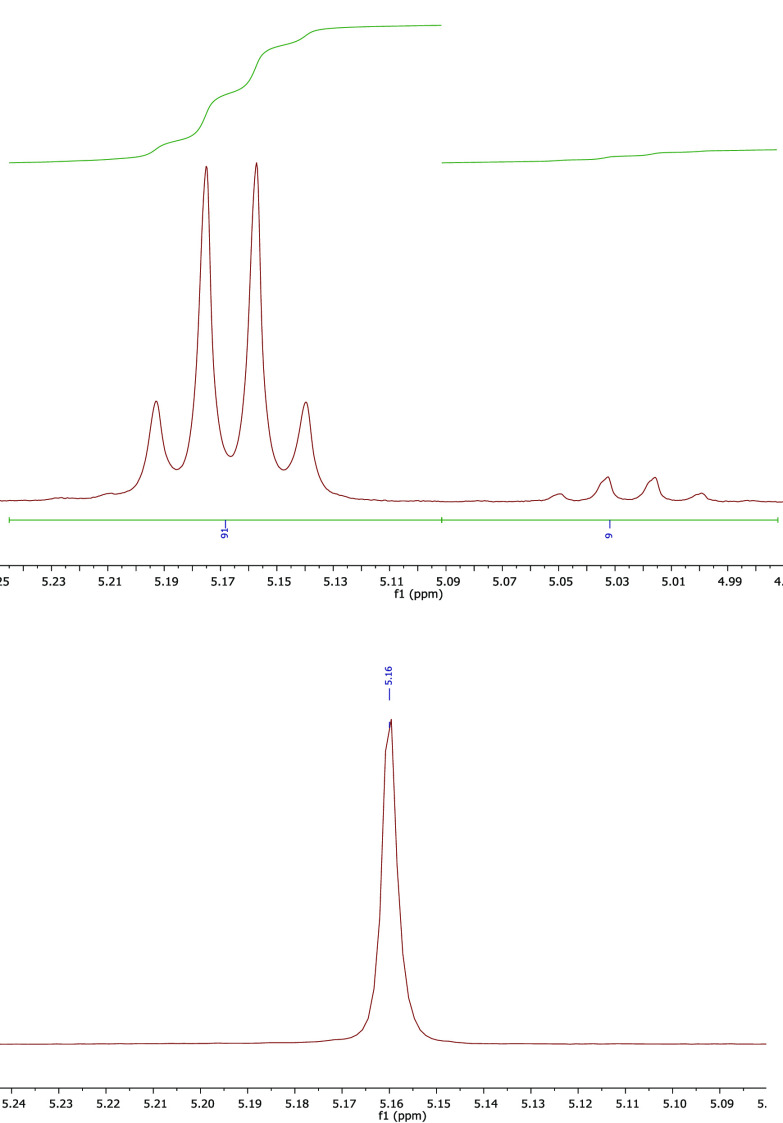
Methine region of the ^1^H NMR spectrum of the crude product from the ROP of l-LA where [Zr] = 2.60 × 10^–2^ mol % ([LA]:[Zr]:[BnOH] = 3850:1:5; 160 ppm Zr by weight) and [BnOH] = 0.13 mol %, containing signals corresponding to isotactic PlLA and residual monomer species (top), and the methine signal from the homonuclear decoupled ^1^H{^1^H} NMR spectrum of the PlLA product of the same reaction, where all linkages are represented by the [*iii*] tetrad (bottom).^83^ 5 g of l-LA, solvent-free, 180 °C, 2 h, solid **1** and BnOH were used.

The apparent resistance of **1** (and ***f1***) toward promoting epimerization was further demonstrated by polymerizing l-LA under conditions anticipated to favor unwanted side reactions (high Zr loading, reduced molar [BnOH]:[Zr] ratio relative to ***f1***, and extended reaction times). For example, entirely isotactic (analysis *via* homonuclear decoupled ^1^H NMR spectroscopy), high-molecular-weight PlLA was readily prepared in the presence of **1**, where [Zr] = 2.60 × 10^–2^ mol % (160 ppm Zr by weight) and [BnOH] = 0.13 mol % ([LA]:[Zr]:[BnOH] = 3850:1:5), and a temperature of 180 °C was maintained for 2 h; *M*_n_^GPC^ = 103,400 g mol^–1^, *M*_n_^Theo^ = 106,500 g mol^–1^, *Đ*_M_ = 1.56, *P*_r_ = 0 (furthermore, where [BnOH] = 1.3 mol %, ([LA]:[Zr]:[BnOH] = 3850:1:50); *M*_n_^GPC^ = 14,200 g mol^–1^, *M*_n_^Theo^ = 11,000 g mol^–1^, *Đ*_M_ = 1.49, *P*_r_ = 0) (Figure 3, see the Supporting Information for further details). Increased polymer dispersity, *Đ*_M_, under those conditions, relative to other studies described in this work, was likely indicative of some transesterification having occurred.

**Figure 4:**
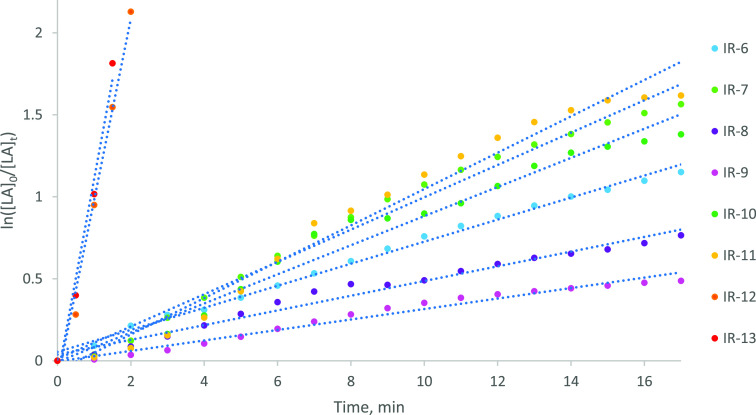
Semilogarithmic initial rate plots for comparison of ***f1***, solid **1**, and Sn(Oct)_2_ at several metal loadings. Labels refer to entry numbers in Table 3. *R*^2^ values for IR-6–IR-13 = 0.99, 0.99, 0.98, 0.98, 0.97, 0.97, 0.99, and 0.98, respectively.

At higher metal loadings than those described in Table 1 ([Zr] = 1.30 × 10^–2^ mol % and [Zr] = 1.95 × 10^–2^ mol %; [LA]:[Zr]:[ROH] = 7700:1:100 and [LA]:[Zr]:[ROH] = 5100:1:100, where [LA] is given to the nearest hundred equivalents; 81 and 121 ppm Zr by weight; Table 3, Figure 4), the ROP of *rac*-LA was generally slower and its kinetics more erratic in the presence of **1** than ***f1*** (see Figure S18 in the Supporting Information), the latter exhibiting characteristic *pseudo*-first-order kinetics. Moreover, the semilogarithmic initial rate plot obtained when the solid catalyst was used at high loading ([Zr] = 1.95 × 10^–2^ mol %) exhibited a distinct S-shaped curvature, attributable to a mixing effect. Nonetheless, polymer molecular weight control and dispersity were similar for the two systems, and consistent with the highly predictable, immortal regime and suppression of side reactions afforded by ***f1*** and related systems, *vide infra*. We attribute this high degree of control to the coordinatively saturated, sterically enclosed nature of the metal center of **1**.

**Table 3:** Polymerization Data for Comparison of Initiators ***f1***, Solid **1**, and Sn(Oct)_2_ at Several Metal Loadings^a^

*Entry*	*Initiator*	*Duration, min*	*[Metal],mol %*	*[Metal],^b^ ppm*	*[ROH],mol %*	Conversion,^c^ %	*M*_*n*_^*Theo*^*,*^d^*g mol^–1^*	*M*_*n*_^*GPC*^*,*^e^*g mol^–1^*	*Đ*_*M*_^e^	*k*_*obs*_*,^f^ min*^*–1*^
*IR-6*	***f1***	75	1.30 × 10^–2^	81	1.30	91	10500	9250	1.08	6.72 × 10^–2^
*IR-7*	***f1***	62	1.95 × 10^–2^	121	1.95	93	7000	6700	1.07	9.86 × 10^–2^
*IR-8*	**1** + BnOH	60	1.30 × 10^–2^	81	1.30	80	9000	8950	1.08	4.48 × 10^–2^
*IR-9*	**1** + BnOH	60	1.30 × 10^–2^	81	1.30	57	6500	6100	1.06	3.19 × 10^–2^
*IR-10*	**1** + BnOH	60	1.95 × 10^–2^	121	1.95	93	7000	6500	1.13	8.88 × 10^–2^
*IR-11*	**1** + BnOH	60	1.95 × 10^–2^	121	1.95	92	7000	7550	1.16	0.1109
*IR-12*	Sn(Oct)_2_ + BnOH	10	1.30 × 10^–2^	105	1.30	96	11000	18550	1.43	1.1042
*IR-13*	Sn(Oct)_2_ + BnOH	10	1.95 × 10^–2^	157	1.95	95	7500	10600	1.35	1.2118

a Conditions: 20 g *rac*-LA, solvent-free, 174 °C. Where [Metal] = 1.30 × 10^–2^, [LA]:[Metal]:[ROH] = 7700:1:100; where [Metal] = 1.95 × 10^–2^, [LA]:[Metal]:[ROH] = 5100:1:100.

b Metal concentration, ppm by weight.

c Conversion determined *via*^1^H NMR spectroscopy, by integration of LA and PLA methine resonances.

d *M*_n_^Theo^ calculated from conversion and alcohol concentration; *M*_n_^Theo,***f*1**^ = {(*M*_r,*rac*-LA_ ×  × ) + *M*_r,BnOH_ + *M*_r,LA_}; *M*_n_^Theo,**1**^ = *M*_n_^Theo,Sn(Oct)_2_^ = {(*M*_r,*rac*-LA_ × ×  + *M*_r,RnOH_}.

e Determined *via* GPC analysis in THF using Triple Detection.

f Determined by initial rate analysis *via in situ* ATR-FT-IR spectroscopic reaction monitoring.

Particles of solid **1** visibly persisted throughout all reactions for which it was used, and its correspondingly irregular kinetic profile may therefore be attributed to the catalyst's poor solubility in the monomer melt, and a possible heterogeneous regime at the particle surface. Rate discrepancies between **1** and ***f1*** were not observed at very low metal loadings ([Zr] = 2.50 × 10^–3^ mol %; 16 ppm Zr by weight; Table 1). While both ***f1*** and solid **1** appeared to afford improved molecular weight control and reduced *Đ*_M_ relative to Sn(Oct)_2_, it is plausible that the apparently lower selectivity of the latter system is due to proliferation of side-reactions (e.g., transesterification, chain scission) following rapid attainment of equilibrium conversion in the presence of the highly active Sn(II) species.

Data from further polymerization studies, with various loadings of ***f1*** (Table 4), were subjected to Variable Time Normalization Analysis (VTNA),^84,85^ followed by initial rate analysis to obtain the propagation rate constant *k*_p_.

**Table 4:** Polymerization Data for Determination of Order in Catalyst, **1**, and Propagation Rate Constant *k*_p_ for the ROP of *rac*-LA Initiated by ***f1***^a^

*Entry*	*Duration, min*	*[Zr],mol %*	*[Zr],^b^ ppm*	*[ROH],mol %*	*Conversion,^c^ %*	*M*_*n*_^*Theo*^*,*^d^*g mol^–1^*	*M*_*n*_^*GPC*^*,^e^g mol^–1^*	*Đ*_*M*_^e^	*k*_*obs*_,^f^*min*^*–1*^
*IR-14*	183	6.50 × 10^–3^	40	0.65	78	17500	7900	1.09	N/A
*IR-15*	160	9.90 × 10^–3^	61	0.99	91	13500	15100	1.08	4.65 × 10^–2^
*IR-6*	75	1.30 × 10^–2^	81	1.30	91	10500	9250	1.08	6.72 × 10^–2^
*IR-16*	105	1.77 × 10^–2^	110	1.76	94	8000	7900	1.10	9.06 × 10^–2^
*IR-7*	62	1.95 × 10^–2^	121	1.95	93	7000	6700	1.07	9.86 × 10^–2^
*IR-17*	60	2.53 × 10^–2^	158	2.53	95	5500	12650	1.09	0.1229
*IR-5*^g^	315	0	0	0.25	4	N/A	N/A	N/A	2 × 10^–4^

a Conditions: 20 g of *rac*-LA, solvent-free, 174 °C, ***f1*** used. [LA]:[Zr]:[ROH]; IR-14 = 15,400:1:100; IR-15 = 10,100:1:100; IR-6 = 7700:1:100; IR-16 = 5600:1:100; IR-7 = 5100:1:100; IR 17 = 4000:1:100; IR-5 = 400:0:1.

b Metal concentration, ppm by weight, calculated assuming [Zr]_***f1***_ = 4.27 × 10^–2^ mol dm^–3^.

c Conversion determined *via*^1^H NMR spectroscopy, by integration of LA and PLA methine resonances.

d *M*_n_^Theo^ calculated from conversion and alcohol concentration:
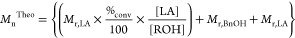

e Determined *via* GPC analysis in THF using Triple Detection.

f Rate constants determined *via* initial rate analysis.

g Control reaction; no catalyst, 0.25 mol % BnOH.

**Figure 5:**
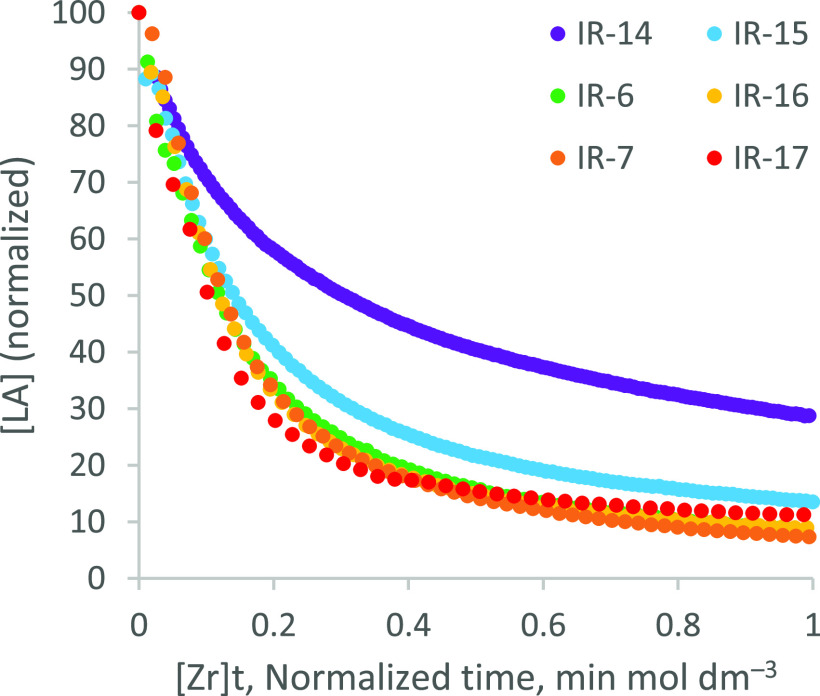
VTNA plots for the ROP of *rac*-LA in the presence of various loadings of ***f1***. Labels refer to entry numbers in Table 4.

VTNA showed that the ROP of LA is first order with respect to ***f1***, characterized by superimposition of plots of [LA] *versus* normalized time, [Zr]*t* (Figure 5). Immortal ROP is typically zero-order with respect to the concentration of growing chains, so the current process is presumed to be first-order with respect to the active catalyst. However, plots corresponding to very low metal loadings deviated from superimposition in a manner consistent with a catalyst deactivation effect, likely due to gradual air ingress over long reaction times. Consequently, data from reaction IR-14 ([Zr] = 6.5 × 10^–3^ mol %; [LA]:[Zr]:[ROH] = 15,400:1:100; 40 ppm Zr by weight), which exhibited the earliest and most dramatic deviation, was disregarded in subsequent initial rate analysis.

Initial rate analysis confirmed that the ROP was first-order with respect to LA (see the Supporting Information), consistent with a chain-growth polymerization governed by the rate equation, eq 1.
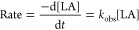
1

**Figure 6:**
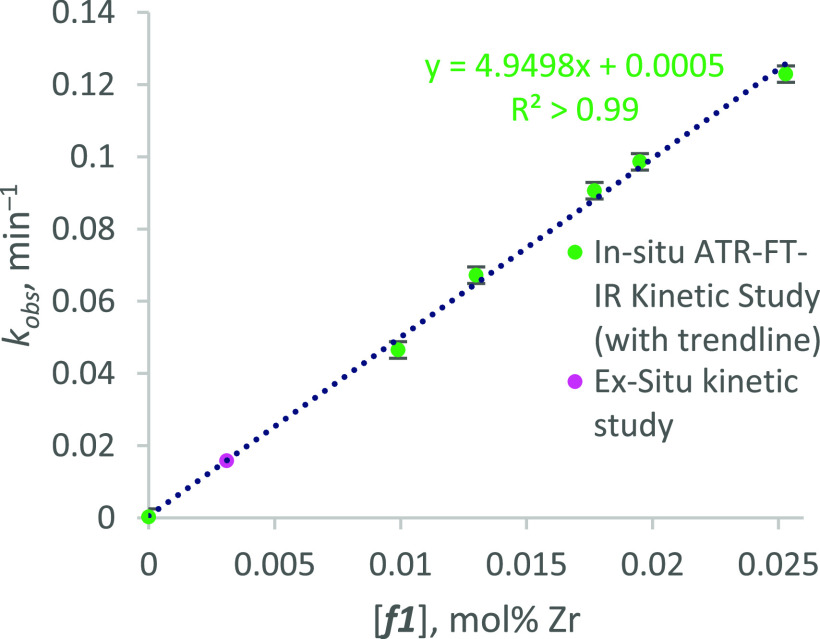
A plot of *k*_obs_*versus* [***f1***], where [***f1***] = [Zr], for determination of the propagation rate constant, *k*_p_, for the ROP of *rac*-LA initiated by ***f1***. Values of *k*_obs_ were obtained from semilogarithmic initial rate plots, constructed for determination of order in catalyst, **1**. Error bars correspond to the root mean squared error.

**Figure 7:**
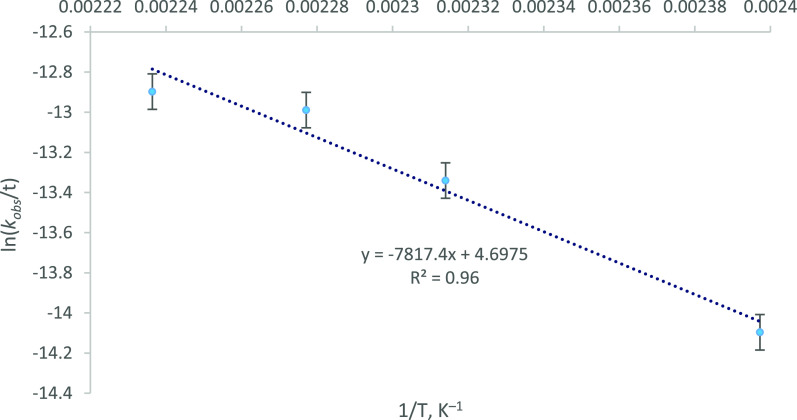
Eyring plot for the determination of Δ*G*^⧧^ for the solvent-free ROP of *rac*-LA at 174 °C in the presence of 1.3 × 10^–2^ mol % Zr ([LA]:[Zr]:[ROH] = 7700:1:100; 15.6 ppm Zr by weight), dosed as ***f1***. Experimental details in Table 5. Error bars correspond to the root mean squared error.

A plot of the observed rate constant, *k*_obs_, against [Zr] (dosed as ***f1***) produced a linear plot with gradient equal to the propagation rate constant, *k*_p_ = 4.9498 min^–1^ mol %^–1^ (7.425 × 10^–3^ s^–1^ mol^–1^ dm^3^) (Figure 6). This confirms that the reaction is first-order with respect to ***f1***, corresponding to an immortal regime, first-order in **1**, and zero-order in growing chains, as described by eq 2, where the values [***f1***] and [**1**] are both equivalent to the molar Zr concentration, [Zr].
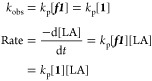
2The inhibitive effect of air ingress over very long reaction times was confirmed by an *ex situ* kinetic study in which ten 1 g-scale polymerizations, employing ***f1*** such that [Zr] = 3.1 × 10^–3^ mol % (19 ppm Zr by weight, [LA]:[Zr]:[ROH] = 32,300:1:100), and undertaken in sealed Young’s ampules under stringent air-free conditions, were terminated after various time intervals. Despite the extremely low Zr concentration, the rate constant, *k*_obs_ = 1.58 × 10^–2^ min^–1^, was in excellent agreement with the rate law and the calculated value of *k*_p_ (eq 3, Figure 6). Moreover, ^1^H NMR spectroscopic analysis of the crude reaction mixtures showed that a catalyst turnover frequency of at least TOF = 25,000 h^–1^ was attained under these conditions (this value being a lower bound, assuming all Zr centers remained catalytically active following preparation of ***f1***).
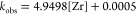
Thus,
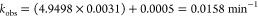
3To support our mechanistic investigation, *vide infra*, Δ*G*^⧧^ for the ROP of LA at 174 °C, initiated by ***f1***, was determined experimentally *via* construction of an Eyring plot using kinetic data collected at 174, 166, 159, and 144 °C, respectively (Table 5, Figure 7). The magnitude of the resulting value, Δ*G*^⧧^ = +32.5 kcal mol^–1^ (+135.9 kJ mol^–1^; Δ*H*^⧧^ = +15.5 kcal mol^–1^, +65.0 kJ mol^–1^; Δ*S*^⧧^ = −37.9 cal K^–1^ mol^–1^, −158.5 J K^–1^ mol^–1^) appears reasonable for a catalytic ROP of LA and compares favorably with literature values for various Sn(II) systems.^86−88^

**Table 5:** Polymerization Data for the ROP of *rac*-LA Catalyzed by ***f1*** at Several Temperatures for the Determination of Δ*G*^⧧^ a

*Entry*	*Temperature, °C*	*Duration, min*	*Conversion,^b^ %*	*M*_*n*_^*Theo*^*,*^c^*g mol^–1^*	*M*_*n*_^*GPC*^*,*^d^*g mol^–1^*	*Đ*_*M*_^d^	*k*_*obs*_,^e^*min*^*–1*^
*IR-6*	174	75	91	10500	9250	1.08	6.72 × 10^–2^
*IR-18*	166	60	88	10000	9050	1.03	6.02 × 10^–2^
*IR-19*	159	120	88	10000	7500	1.04	4.17 × 10^–2^
*IR-20*	144	240	84	9500	5100	1.02	1.89 × 10^–2^

a Conditions: 20 g *rac*-LA, solvent-free, ***f1*** used. [Zr] = 1.3 × 10^–2^ mol %, [ROH] = 1.3 mol % ([LA]:[Zr]:[ROH] = 7700:1:100; 15.6 ppm Zr by weight).

b Conversion determined *via*^1^H NMR spectroscopy, by integration of the monomer and polymer methine resonances.

c *M*_n_^Theo^ calculated from conversion and alcohol concentration:
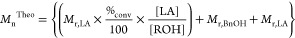

d Determined *via* GPC analysis in THF using Triple Detection.

e Rate constants determined by initial rate analysis *via in situ* ATR-FT-IR spectroscopic reaction monitoring.

### Protocol Optimization and Scale-up

**Figure 8:**
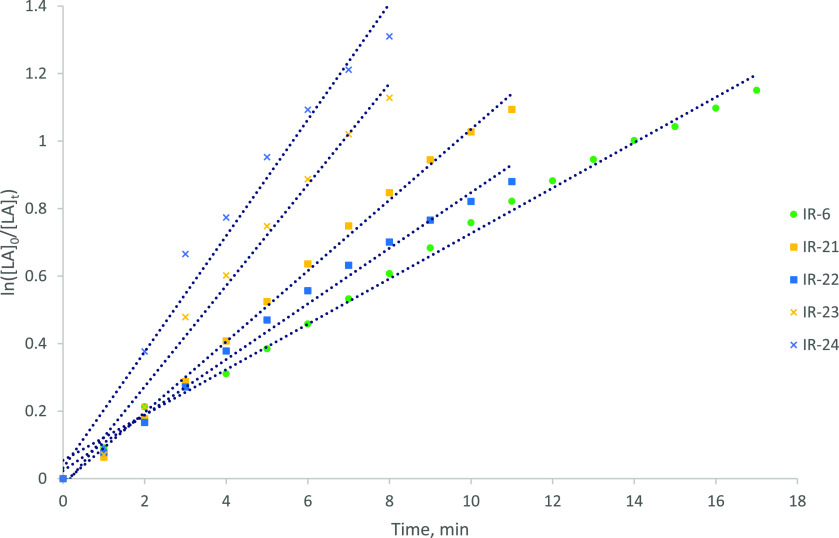
Semilogarithmic initial rate plots for the ROP of *rac*-LA in the presence of ***f1***, ***f2***, and ***f3***, at equimolar concentrations of Zr and alcohol, respectively. Labels refer to entry numbers in Table 6. *R*^2^ values for IR-6 and IR-21–IR-24 = 0.99, 0.99, >0.99, 0.99, and 0.98, respectively.

Encouraged by the catalytic performance of ***f1***, two further formulations of **1** were prepared, denoted ***f2*** and ***f3***, in which the concentration of **1** was reduced, such that [Zr]_***f2***_ = 0.67 mol % and [Zr]_***f3***_ = 0.50 mol %. Both ***f2*** and ***f3*** were much more active than anticipated for the ROP of LA, with ATR-FT-IR data indicating remarkable TOF values of up to at least 56,000 h^–1^ (Table 6; Figure 8). This is attributed to reduced degradation of **1** occurring during the formulation process for ***f2*** or ***f3*** relative to that for ***f1***, due to the shorter heating time required (35 min for ***f2***; 30 min for ***f3***; 50 min for ***f1***), and is consistent with **1** being the active catalyst. Nonetheless, it is feasible, and reconcilable with the proposed mechanism, that a contributory factor is a non-zero-order rate dependence on [ROH] below a threshold molar ratio of [ROH]:[Zr] < 150:1 (see the Supporting Information).^29,32^

**Table 6:** Polymerization Data for the Application of ***f1***, ***f2***, and ***f3*** to the ROP of *rac*-LA, at Equimolar Concentrations of Zr and ROH, Respectively^a^

*Entry*	*Formulation*	*Duration, min*	*[Zr],mol %*	*[Zr],^b^ ppm*	*[ROH],mol %*	*Conversion,^c^ %*	*M*_*n*_^*Theo*^*,*^d^*g mol^–1^*	*M*_*n*_^*GPC*^*,*^e^*g mol^–1^*	*Đ*_*M*_^e^	*k*_*obs*_,^f^*min*^*–1*^	*TOF,^g^ h*^*–1*^
*IR-6*	***f1***	75	1.3 × 10^–2^	81	1.30	92	10464	9250	1.08	6.72 × 10^–2^	23000
*IR-21*	***f2***	90	8.7 × 10^–3^	54	1.30	93	10576	9650	1.10	0.1049	50500
*IR-22*	***f3***	180	6.5 × 10^–3^	40	1.30	94	10687	10050	1.11	8.24 × 10^–2^	45500
*IR-23*	***f2***	60	1.3 × 10^–2^	81	1.95	93	7120	6200	1.07	0.1496	46000
*IR-24*	***f3***	75	1.3 × 10^–2^	81	2.60	94	5478	5200	1.06	0.1720	56000

a Conditions: 20 g of *rac*-LA, solvent-free, 174 °C. [LA]:[Zr]:[ROH]; IR-6 = 7700:1:100, IR-21 = 11,500:1:150, IR-22 = 15,400:1:200, IR-23 = 7700:1:150, IR-24 = 7700:1:200.

b ppm Zr by weight.

c Conversion determined *via*^1^H NMR spectroscopy, by integration of LA and PLA methine resonances.

d *M*_n_^Theo^ calculated from conversion and alcohol concentration:
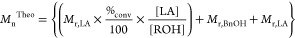

e Determined *via* GPC analysis in THF using Triple Detection.

f *k*_obs_ determined *via* initial rate analysis.

g TOF calculated from percentage conversion, determined *via*^1^H NMR spectroscopy, , using the data point for which [LA] is closest to 40 mol %: IR-6, [LA] = 39.7 mol % at 12 min; IR-21, [LA] = 41.2 mol % at 8 min; IR-22, [LA] = 40.7 mol % at 12 min; IR-23, [LA] = 40.0 mol % at 6 min; IR-24, [LA] = 39.2 mol % at 5 min. Values reported to the nearest 500.

Even where the alcohol concentration was equivalent, the absolute rates afforded by ***f2*** and ***f3*** exceeded that produced by ***f1***, despite the metal loading being significantly lower in both cases than for ***f1***. These results presented the possibility of preparing high-molecular-weight PLA on an industrially relevant time scale in the presence of extraordinarily low metal concentrations. ***f1***, ***f2***, and ***f3*** were therefore applied to the ROP of commercial polymer-grade l-LA at 180 °C under industrially relevant conditions on a 500–2000 g scale (Table 7), to exemplify the performance of the catalyst under such demanding conditions.

**Table 7:** Polymerization Data for the ROP of l-LA in the Presence of Catalyst Formulations ***f1***, ***f2***, and ***f3***, under Industrially Relevant Conditions^a^

*Entry*	*Formulation*	*Duration, min*	*[Metal],^f^ ppm*	*[Metal],mol %*	*[ROH],mol %*	*Conversion,^g^ %*	*M*_*n*_^*Theo*^*,*^h^*g mol^–1^*	*M*_*n*_^*GPC*^*,*^i^*g mol^–1^*	*Đ*_*M*_^i^	*k*_*obs*_,^j^*min*^*–1*^
*IE-1*^b^^,^^k^	***f1***	210	18 + 9	2.9 × 10^–3^ +1.5 × 10^–3^	0.29 + 0.15	71	N/A	26900, 9700^l^	1.17^l^	4.20 × 10^–3^
*IE-2*^c^	***f3***	120	50	8.1 × 10^–3^	1.62	94	8500	8750	1.18	3.16 × 10^–2^
*IE-3*^c^	***f3***	1350	9	1.5 × 10^–3^	0.29	91	45500	37700	1.30	3.40 × 10^–3^
*IE-4*^c^	***f2***	240	12	1.9 × 10^–3^	0.29	78	39000	32200	1.27	7.80 × 10^–3^
*IE-5*^d^	***f2***	390	8	1.3 × 10^–3^	0.19	70	53500	41350	1.36	3.50 × 10^–3^
*IE-6*^e^	Sn(Oct)_2_ +Co-In.^m^	120	44	5.3 × 10^–3^	0.29	93	46000	41800	1.94	4.01 × 10^–2^

a Conditions: l-LA, solvent-free, 180 °C. [LA]:[Zr]:[ROH]; IE-1 = 34,500:1:100, then 22,700:1:100 (w.r.t. [LA] at *t* = 0), IE-2 = 12,300:1:200, IE-3 = 66,700:1:200, IE-4 = 52,600:1:150, IE-5 = 76,900:1:150. IE-6 [LA]:[Sn]:[ROH] = 18,900:1:55.

b 2000 g LA.

c 500 g LA.

d 600 g LA.

e 750 g LA.

f [Metal], ppm by weight, calculated assuming [Zr]_***f1***_ = 4.270 × 10^–2^ mol dm^–3^, [Zr]_***f2***_ = 2.850 × 10^–2^ mol dm^–3^, [Zr]_***f3***_ = 2.135 × 10^–2^ mol dm^–3^.

g Conversion determined *via*^1^H NMR spectroscopy, by integration of LA and PLA methine resonances.

h *M*_n_^Theo^ calculated from conversion and alcohol concentration (mol %, 2 d.p.), , except in the case of reaction IE-6, where the *M*_r_ of the initiator has not been considered.

i Determined *via* GPC analysis in THF using a refractive index detector, calibrated against polystyrene standards. A Mark–Houwink factor of 0.58, suitable for isotactic PlLA, has been applied.

j Rate constants determined by initial rate analysis, using conversion values determined by ^1^H NMR analysis of aliquots taken from the reactor during polymerization.

k Additional catalyst formulation ***f1*** was added after 2 h.

l Molecular weight distribution bimodal (see the Supporting Information).

m Co-initiator, “Co-In”, not disclosed due to commercial sensitivity.

As anticipated, ***f2*** remained the most active system under industrially relevant conditions, while ***f1*** and ***f3*** afforded similar rates to one another in the preparation of PLA of equivalent theoretical molecular weight, despite a 2-fold difference in [Zr]. ***f2*** produced PLA of >30,000 g mol^–1^ in 3 h, where [Zr] = 1.9 × 10^–3^ mol % ([LA]:[Zr]:[ROH] = 52,600:1:150; 12 ppm Zr by weight), and surpassed 40,000 g mol^–1^ in 6 h, where [Zr] = 1.3 × 10^–3^ mol % ([LA]:[Zr]:[ROH] = 76,900:1:150; 8 ppm Zr by weight). The latter reaction was terminated after 6.5 h, by which time the catalyst turnover number (TON) had exceeded 58,000 (lower bound, assuming all Zr centers were catalytically active) and a polymerization using ***f3*** ([Zr] = 1.5 × 10^–3^ mol %, [ROH] = 0.29 mol %; [LA]:[Zr]:[ROH] = 66,700:1:200; 9 ppm Zr by weight) exceeded a TON of 60,000, reaching 91% conversion after 22.5 h and yielding PLA of *M*_n_ = 38,000 g mol^–1^.

Where ***f3*** was used, such that [Zr] = 8.1 × 10^–3^ mol % ([ROH] = 1.62 mol %; [LA]:[Zr]:[ROH] = 12,300:1:200; 50 ppm Zr by weight), the rate constant, *k*_obs_ = 3.16 × 10^–2^ min^–1^, was similar to that observed in the presence of 5.3 × 10^–3^ mol % Sn(Oct)_2_ ([Co-In] = 0.29 mol %; [LA]:[Sn]:[ROH] = 18,900:1:55; 44 ppm of Sn by weight), *k*_obs_ = 4.01 × 10^–2^ min^–1^. Accordingly, under comparable conditions, the TOF of the Sn centers exceeded that of Zr (assuming all Zr centers were active) by a factor of less than 2 (TOF_Sn_:TOF_Zr_ = 1.94:1), and the activity ratio of Sn *versus* Zr, *per* unit metal weight, was just 1.44:1. By contrast, the TOF of the respective metal centers in ***f3*** and Sn(Oct)_2_ in 20 g-scale studies using recrystallized *rac*-LA differed by a factor of 6.4. The much smaller activity discrepancy observed under industrial conditions, despite the lower polymerizability of l-LA than *rac*-LA in the presence of **1** and ***f1***, and therefore presumably ***f2*** and ***f3***, suggests that **1** and associated formulations are more robust, particularly toward deactivation by acidic contaminants, than Sn(Oct)_2_. We attribute this to kinetic stabilization afforded by the amine tris(phenolate) ligand environment. Although such comparisons between ***f3*** and Sn(Oct)_2_ do not consider the difference in [Sn]:[ROH] ratio between 20 g-scale and ≥500 g-scale reactions, in all cases, this value far exceeded the threshold above which solution-phase Sn(Oct)_2_- and other Sn(II)-catalyzed LA ROP processes have been shown to be zero-order with respect to the co-initiator.^29,32^

Although the protocols described here are evidently robust toward industrial conditions, a slight reduction in *M*_n_ relative to theoretical values, and increase in *Đ*_M_, in comparison to 20 g-scale studies, was attributed to some co-initiation of ROP by protic contaminants. Nonetheless, control was consistently good, and transesterification minimal. This was confirmed by introduction of additional ***f1*** to reaction IE-1 after 2 h (40% conversion), with the reaction conditions being maintained for a further 1.5 h thereafter (reaching 71% conversion). GPC analysis of the product revealed two distinct polymer distributions (*M*_n_ = 26,900 g mol^–1^, *Đ*_M_ = 1.17; *M*_n_ = 9300 g mol^–1^, *Đ*_M_ = 1.02). Polymer molecular weight increased linearly with conversion in all reactions undertaken under industrial conditions (see the Supporting Information), with no epimerization evident, indicating excellent adherence to immortal kinetics with suppression of side-reactions. Differential scanning calorimetry (DSC) of the crystalline resin pellets produced from the product of reaction IE-3 revealed a melting event with maximum negative heat flow occurring at 173.25 °C (see Figure S56 in the Supporting Information). This is consistent with literature values for isotactic P*L*LA of comparable molecular weight, confirming the absence of any detectable epimerization activity in the presence of **1** (Table 2, Figure 3).^89^ While Sn(Oct)_2_ remains more active than **1**, GPC data indicated significant deviation from immortal polymerization kinetics in the presence of that species (*Đ*_M_ = 1.94), particularly at high conversion. This can likely be attributed to the proliferation of side reactions under conditions of low residual monomer concentration. Reduction of polymer molecular weight and a small but observable increase in monomer concentration late in the course of reaction IE-6 (Figures S40 and S41 in the Supporting Information) are suggestive of Sn(II)-catalyzed backbiting activity, while the occurrence of chain scission reactions is also feasible. It is similarly likely that end-to-end cyclization of the polymer chains occurred during the latter phase of the Sn(Oct)_2_-catalyzed reaction, as recently described by Kricheldorf, resulting in the presence of a small molar percentage of macrocyclic PLA among the linear bulk.^90−92^ Moreover, the impressive utility of the formulations ***f1***, ***f2***, and ***f3***, in efficiently producing PlLA of high, well-defined molecular weight, with metal loadings as low as 1.3 × 10^–3^ to 1.9 × 10^–3^ mol % (8–12 ppm Zr by weight), confirms their genuine viability for the commercial production of optically pure PLA of extremely low metal content.

### Mechanistic Study

The catalytic ROP of LA under a high-temperature, solvent-free regime is not readily amenable to direct mechanistic investigation. Accordingly, motivated by the apparent structural incompatibility of **1** with standard coordination–insertion or activated monomer mechanisms, a combined theoretical–experimental approach was taken to investigate the LA polymerization pathway in the presence of **1** and BnOH. Therein, the proposed mechanism, determined *via* application of density functional theory (DFT), was validated by the results of a kinetic isotope study and the experimental determination of Δ*G*^⧧^, described above.

Due to the size of the catalytic system (involving up to 159 atoms in the initiation step), a layered basis set protocol, involving the Stuttgart/Dresden effective core potential (SDD ECP) and associated basis set for the Zr center, was used. To best approximate solvation in molten LA and PLA, the solvent phase was modeled as ethyl acetate, using a self-consistent reaction-cavity continuum solvation model (cpcm),^93,94^ and calculations were undertaken using a temperature of 453.15 K (180 °C).

The computational study modeled the transition states and intermediates associated with the various plausible mechanistic pathways (see the Supporting Information for full details). The possibility of a coordination–insertion mechanism was considered, requiring displacement of one amine tris(phenolate) ligand, *via* protonation of one phenolate group by BnOH and two intramolecular proton transfer events from the respective ligands’ tertiary ammonium groups, to form a heteroleptic Zr benzyl alkoxide species. This was calculated to be thermodynamically inaccessible under the reaction conditions (Δ*G* = +50.7 kcal mol^–1^). Similarly, a coordination–insertion mechanism, proceeding *via* formation of a zwitterionic benzyl alkoxide through displacement of a single phenolate group, is unviable. The energetic barrier to nucleophilic attack, in that circumstance requiring dissociation of a second phenolate group to enable coordination of LA, is insurmountable.

**Figure 9:**
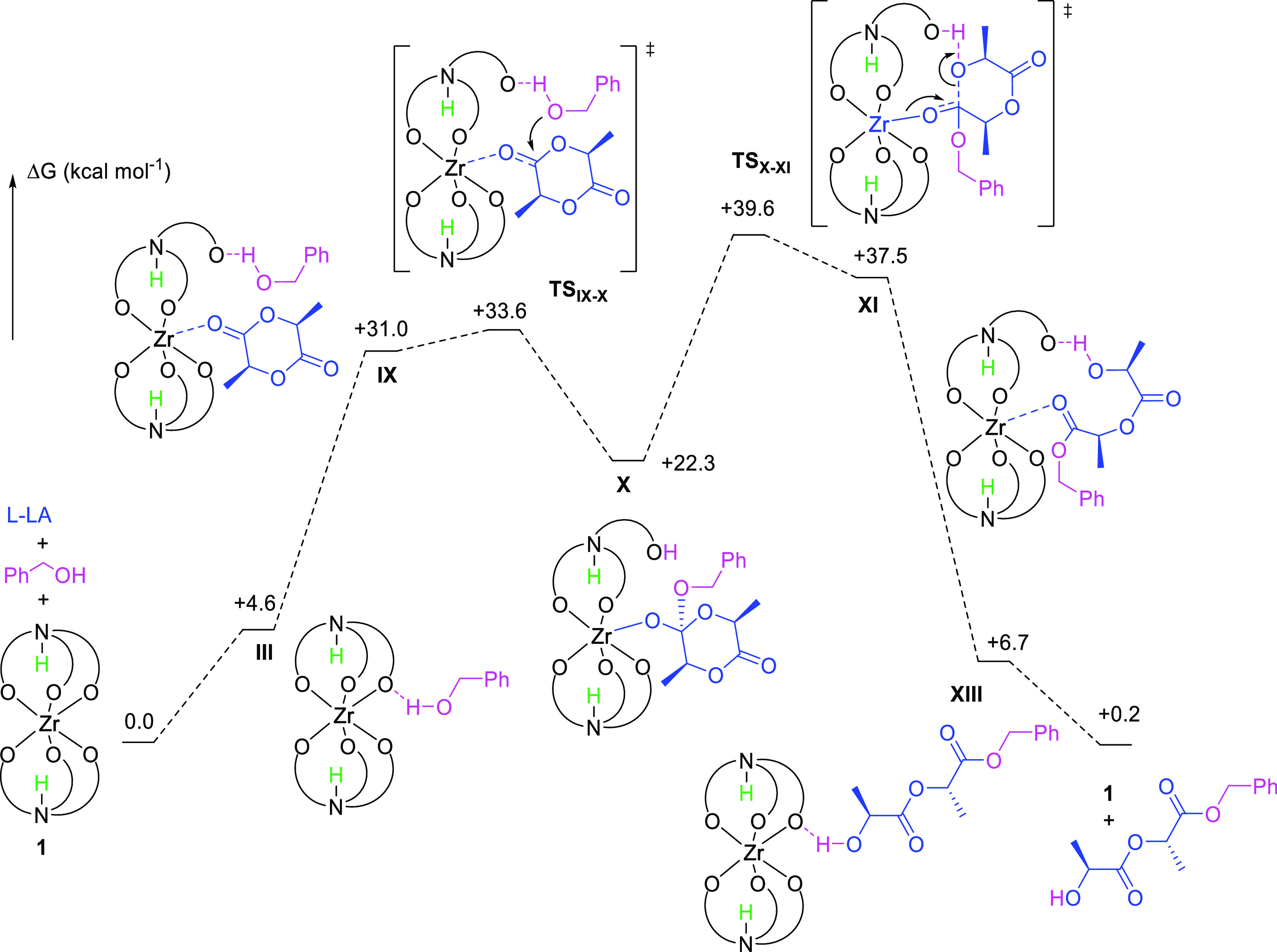
Free enthalpy profile of the favored mechanism for the initiation step of the ROP of l-LA in the presence of **1** and BnOH, calculated using the PBE0-D3 protocol. Propagation is anticipated to follow an analogous pathway.

Finally, a classical activated monomer mechanism was ruled out, due to steric congestion at the metal center obstructing coordination of LA. It was determined in modeling the interaction of **1** with BnOH that a binary hydrogen-bonded complex of **1** and BnOH (**III** in Figure 9) was the likely resting state of the catalyst under the experimental conditions of excess alcohol with respect to **1** (Δ*G* = +4.6 kcal mol^–1^), reminiscent both of Carpentier’s LAAM mechanism^13^ and of Kricheldorf and others’ studies of the interaction between the alcohol and octanoate ligand in the initiation of Sn(Oct)_2_-mediated ROP.^34−36^

In the presence of excess LA, as under solvent-free ROP conditions, it is plausible that protonation of a phenolate group by BnOH be followed by occupation of the resulting vacant coordination site by LA, and this is calculated to be thermodynamically accessible (**IX** in Figure 9). Thereafter, nucleophilic attack on the activated monomer by the alcohol, itself activated by the anionic phenolate, is the most accessible pathway (**TS**_**IX–X**_ and **X** in Figure 9). From the resulting species, several distinct ring-opening events were modeled, yielding the LAAM pathway described by Figure 9 as the favored mechanistic route. This is expected to return the “resting state” of **1** and the growing chain in a hydrogen-bonded binary complex, **XIII**, analogous to **III**, closing the catalytic cycle. The metal center of **1** remains very enclosed throughout the calculated pathway, with exposure of only a single, highly constrained coordination site at which activation of LA can occur. This is compatible with the observed resistance of the system toward undesirable side-reactions, in clear contrast to the exposed metal center and correspondingly lower selectivity of Sn(Oct)_2_.

The slight endergonicity we observe in modeling the ROP of LA has precedent in the work of Gibson and Rzepa,^95,96^ among others.^97^ Only initiation was modeled, due to the computational resources demanded by the large size of the system, and the limiting free energy of the ROP process has therefore not necessarily been accurately represented. Indeed, with an increased number of monomer units, the entropy of the system is anticipated to increase, and the free enthalpy of polymerization is therefore expected to become more favorable as polymerization progresses, in closer agreement with the experimental value of Δ*G*^⧧^. Accordingly, propagation is expected to be mechanistically analogous to initiation, with **1** consequently being the active catalyst, rather than a precatalyst. All transition states were reoptimized and computed using different functionals (ωB97XD and M06-D3; see the Supporting Information) confirming the favored LAAM pathway.

Although energetic barriers calculated at 180 °C were considered high (Δ*G*^⧧^ = +39.6 kcal mol^–1^), transition state theory modeling indicated that mechanistic events with values below Δ*G*^⧧^ = +40 kcal mol^–1^ were accessible under the current conditions, and the experimentally determined Δ*G*^⧧^ value for the ROP of LA in the presence of **1**, Δ*G*^⧧^ = +32.5 kcal mol^–1^, was of a similar magnitude. The transition state for the rate-determining step was also recalculated at 174 °C, to ensure consistency with experimental work, and no significant difference in the activation barrier was observed. Finally, all transition states were reoptimized and computed at 25 °C. The LAAM mechanism remained the favored pathway at that temperature. The limiting activation barrier for the LAAM pathway at 25 °C was Δ*G*^⧧^ = +24.5 kcal mol^–1^, typical for a ROP under those conditions.

In the accompanying kinetic isotope study (see section 2.3 in the Supporting Information), which was undertaken at 150 °C to aid discernment of any rate discrepancy, 3.1 × 10^–3^ mol % **1** (19 ppm Zr by weight) was introduced, as a solution in toluene, to two sets of seven parallel 1 g-scale reactions, co-initiated by (0.31 mol %) *protio*-ethanol EtO^1^H (^2^D at natural abundance) and *deutero*-ethanol EtO^2^D, respectively ([LA]:[Zr]:[EtO^1^H/^2^D] = 32,300:1:100). The relative rates corresponding to the two alcohol isotopologues, *k*_obs,EtOH_ = 0.0166 ± 0.001896 min^–1^ and *k*_obs,EtOD_ = 0.0121 ± 0.001108 min^–1^, reveal a modest kinetic isotope effect, there being a 27% rate reduction for ^2^D, relative to ^1^H. This magnitude is consistent with the rate-determining step identified computationally, involving partial transfer of the labeled hydrogen atom from the alcohol (or growing chain) to a ligand phenolate group dissociated from the metal center of **1**, and therefore supports the proposed mechanism.

## Conclusion

In conclusion, we report a robust, highly active, and inexpensive catalyst, based around a benign and earth-abundant metal as a credible alternative to Sn(Oct)_2_ for the melt ROP of lactides. A method for the preparation of stable liquid formulations of the catalyst, ***f1***, ***f2***, and ***f3***, has been developed and optimized, yielding systems suitable for large-scale preparation and storage, and compatible with both bulk polymerization infrastructure and continuous dosing of the catalyst. Furthermore, the *in situ* catalytic formation of an oligomeric matrix eliminates any need for exogenous solvent, enhancing industrial relevance and improving adherence to the Principles of Green Chemistry.^78^ Comprehensive kinetic study and polymer analysis has shown that the ROP of lactides in the presence of such formulations is a predictable and well-controlled, immortal process, tolerant to variations in reaction conditions, highly resistant to undesirable side-reactions, and adherent to a first-order rate dependence with respect to the catalyst. Suitability for industrial use is demonstrated by the preparation of high-molecular-weight PlLA from commercial polymer-grade l-LA on an industrially relevant time scale, in the presence of extraordinarily low catalyst loadings, and under a variety of conditions. A computational study has elucidated and confirmed the thermodynamic feasibility of an unusual ligand-assisted activated monomer mechanism, accessible under the reaction conditions. Experimental findings are consistent with the proposed mechanism.

## Supporting Information Available

Complete experimental procedures and analytical details, additional kinetic data and analysis, and other Supporting Information (PDF)

## Notes

Some of the work described in this article is protected by the following patents: WO2014177543 (A1) (Method to Manufacture PLA Using a New Polymerization Catalyst) and WO2021094525 (A1) (Process For Polymerizing Lactide Using a Liquid Catalyst Formulation)
