# Supplementary material for: A Highly Active and Selective Zirconium-Based Catalyst System for the Industrial Production of Poly(lactic acid)
Source: ACS Catal. 2023 Feb 7;13(4):2681–95. doi: 10.1021/acscatal.2c05690 (PMC9942235; doi:10.1021/acscatal.2c05690)
Supplement: Supplementary file 1 — cs2c05690_si_001.pdf [file cs2c05690_si_001.pdf]

Supporting Information

A Highly Active and Selective Zirconium-based Catalyst System for the Industrial Production of Poly(lactic acid)

Antoine Buchard,[1,2] Christopher J. Chuck,[1,3] Matthew G. Davidson,[1,2*] Gerrit Gobius du Sart,[4] Matthew D. Jones,[1,2] Strachan N. McCormick[1,2*] and Andrew D. Russell[2]

Affiliations:

[1]Institute for Sustainability, University of Bath, Bath BA2 7AY, UK.

[2]Department of Chemistry, University of Bath, Bath BA2 7AY, UK.

[3]Department of Chemical Engineering, University of Bath, Bath BA2 7AY, UK.

[4]TotalEnergies Corbion, Arkelsedijk 46, 4206 AC Gorinchem, The Netherlands

*Correspondence to: S.N.McCormick@bath.ac.uk; M.G.Davidson@bath.ac.uk

Contents

1 Materials and methods 8

1.1 Experimental procedures 8

1.2 Synthetic procedures and analytical data 10

Figure S1. Pro-ligands H_3L^{Me}, $H_3L^{Me/tBu}$, and zwitterionic complexes $Zr(HL^{Me})_2$, **1**; and $Zr(HL^{Me/tBu})_2$, **2** 10

1.3 Physical properties of complexes 1 and 2 11

Figure S2. Solubility curve for catalyst **1** in toluene-d_8, produced using variable-temperature NMR spectroscopy 12

Figure S3. Solubility curve for catalyst **1** in *o*-xylene-d_{10}, produced using variable-temperature NMR spectroscopy 12

Table S1. Solubility values of **1** in a range of solvents from crude solvent screening 13

Figure S4. TG and dTG curves corresponding to the thermal decomposition of **1** (TG shown in red, dTG in orange) and **2** (TG shown in blue, dTG in green) under an argon atmosphere 14

1.4 Preparation of catalyst formulations 14

1.4.1 Preparation of *f1* 14

Scheme S1. Diester ***e1***, the product of the ring-opening of one equivalent of lactide in the presence of a benzyl alcohol nucleophile, and heavier oligomers arising from transesterification of ***e1***, comprising the solvent system of catalyst formulation ***f1*** 15

Figure S5. The mass spectrum of catalyst formulation ***f1***, showing lactyl oligomers of up to seven lactic acid repeat units. All are observed as the corresponding Na^+ adducts 16

Figure S6. The ^1H NMR spectrum of catalyst formulation ***f1*** (400 MHz, $CDCl_3$, 298 K), consistent with the presence of benzyl ester ***e1*** and heavier lactyl oligomers. Signal at 7.26 ppm is residual $CHCl_3$ 16

1.4.2 Preparation of *f2* and *f3* 17

Table S2. Molar composition of catalyst formulations ***f1***, ***f2***, ***f3***, and heating time required for preparation of each formulation 17

1.5 *In-situ* ATR-FT-IR-monitored polymerization of *rac*-LA or *L*-LA 18

1.5.1 General procedure 18

Figure S7. A photograph of the jacketed glass reactor used for ATR-FT-IR-monitored polymerization reactions. ATR-FT-IR insertion probe (left), Schlenk line attachment (center) and mechanical stirrer (right) are *in-situ*. Heat transfer fluid was passed through the outer jacket *via* insulated tubing (foreground) 18

Figure S8. Example three-dimensional plot showing change of *rac*-LA and PLA ATR-FT-IR signals spectra corresponding to the solvent-free ROP of *rac*-LA in the presence of catalyst formulation ***f1*** 20

Figure S9. Two-Dimensional ATR-FT-IR spectra corresponding to the solvent-free ROP of *rac*-LA in the presence of catalyst formulation ***f1***; lactide and poly(lactic acid) signals corresponding to the C-O-C stretching mode appear at wavenumber regions 1203-1265 cm^{-1} and 1160-1200 cm^{-1}, respectively 21

Figure S10. Two-Dimensional ATR-FT-IR spectra corresponding to the solvent-free ROP of *rac*-LA in the presence of catalyst formulation ***f1*** with visualised signal integration method; lactide and poly(lactic acid) signals corresponding to the C-O-C stretching mode appear at wavenumber regions 1203-1265 cm^{-1} and 1160-1200 cm^{-1}, respectively.................................... 21

1.5.2 Calibration of ATR-FT-IR reaction monitoring apparatus... 22

Figure S11. Calibration curve for determination of *rac*-LA concentration from integrated ATR-FT-IR signal area at 174 °C 22

Equation S1. Equation describing the relationship between integrated lactide ATR-FT-IR signal area and concentration 22

Figure S12. Calibration curves for determination of *rac*-LA concentration from integrated ATR-FT-IR signal area at various temperatures 23

Figure S13. Variation of integrated *rac*-LA ATR-FT-IR signal area with temperature for *rac*-LA/ PLA mixtures of various composition 23

Equation S2. Equation describing the relationship between integrated lactide ATR-FT-IR signal area and concentration 24

Figure S14. Calibration curve for determination of PLA concentration from integrated ATR-FT-IR signal area at 174 °C 24

Figure S15. Calibration curves for determination of PLA concentration from integrated ATR-FT-IR signal area at various temperatures 25

Figure S16. Variation of integrated PLA ATR-FT-IR signal area with temperature for *rac*-LA/ PLA mixtures of various composition 25

2 Kinetic studies of lactide polymerization... 26

2.1 *In-situ* ATR-FT-IR-monitored polymerization of *rac*-LA or *L*-LA.................................... 26

2.1.1 Plots of lactide concentration *versus* time for various catalyst loadings 26

Table S3. Polymerization data for determination of order in catalyst, **1**, and propagation rate constant k_p for the ROP of *rac*-LA initiated by ***f1*** (reproduced from main paper) 26

Figure S17. Plots of [*rac*-LA] versus time, for reactions IR-14, IR-15, IR-6, IR-16, IR-7, and IR-17, time-resolved data showing the variation in reaction times required to reach high conversion at different catalyst loadings. Labels refer to entry numbers in Table S3.................................... 27

Figure S18. Plots of [*rac*-LA] versus time, for reactions IR-6, IR-7, IR-8, IR-9, IR-10, and IR-11, showing the erratic nature of the kinetics afforded by solid catalyst **1**, relative to the predictable reaction course produced by ***f1***. Labels refer to entry numbers in Table 3 of the main paper.. 27

2.1.3 Variable Time Normalisation Analysis 28

2.1.4 Initial rate analysis... 29

Figure S19. Semi-logarithmic initial-rate plots for the ROP of *rac*-LA in the presence of ***f1***. Labels refer to entry numbers in Table S3.. 29

2.1.5 Addition of exogenous alcohol during polymerization 30

Figure S20. Semi-logarithmic plots corresponding to reactions carried out to investigate the effect of exogenous BnOH on the ROP of *rac*-LA in the presence of ***f1***. Vertical lines denote addition of BnOH. Labels refer to entries in Table S4. 30

Table S4. Polymerization data corresponding to reactions IR-6, IR-25, and IR-26, carried out to investigate the effect of exogenous BnOH on the ROP of *rac*-LA in the presence of ***f1*** 30

Figure S21. Plots of LA concentration against time for reactions IR-6, IR-25 and IR-26. Labels refer to entries in Table S4. 31

Figure S22. Plots of relative rate for reaction IR-25, the ROP of *rac*-LA in the presence of ***f1***, with stepwise addition of aliquots of exogenous BnOH at four-minute intervals (indicated by vertical lines). Two plots have been constructed, using calibrated data corresponding to the LA and PLA signals, respectively. Labels refer to entries in Table S4. 32

Figure S23. A visual representation of the chain growth anticipated to occur for the distribution of polymer chains corresponding to each initiation event in IR-25 (***f1***, followed by four aliquots of BnOH) in each four-minute interval, expressed as a percentage of the monomer feed, and the resulting theoretical molecular weight of each distribution, assuming all chains propagate at an equal rate at any given time 34

2.2 Determination of k_{obs} *via ex-situ* kinetic analysis using parallel reactions 34

2.2.1 General procedure 34

Table S5. Polymerization data for *ex-situ* kinetic study of ROP of lactide catalyzed by ***f1*** 35

Figure S24. Reaction profile (conversion against time) for *ex-situ*-monitored 1 g-scale ROP of *rac*-LA catalyzed by ***f1*** (Table S5, Entries B-1 – B-10). 36

Figure S25. Semi-logarithmic initial rate plot for *ex-situ*-monitored 1 g-scale ROP of *rac*-LA catalyzed by ***f1*** (Table S5, Entries B-1 – B-4) 36

2.3 Kinetic isotope study 36

2.3.1 General procedure 36

Table S6. Polymerization data for kinetic isotope study of the ROP of *rac*-LA in the presence of solid **1** and exogenous ethanol 38

Figure S26. Semi-logarithmic plots for the ROP of *rac*-LA catalyzed by **1** in the presence of exogenous *deutero*- and *protio*-ethanol. Data for all constituent reactions is in Table S6. 38

2.4 Experimental determination of ΔG‡ for the rate-determining step of propagation in the ROP of *rac*-LA initiated by *f1* 39

Table S7. Polymerization data for the ROP of *rac*-LA catalyzed by ***f1*** *at* several temperatures for the determination of ΔG‡ (reproduced from main paper) 40

Figure S28. Eyring plot for the determination of ΔG‡ for the ROP of *rac*-LA in the presence of ***f1*** 41

3 Polymerization of *L*-LA in the presence of *f1, f2* and *f3* under industrially relevant conditions 41

3.1 General procedure 41

Table S8. Polymerization data for the ROP of *L*-LA in the presence of catalyst formulations ***f1***, ***f2***, and ***f3***, under industrial conditions (reproduced from main paper) 42

Figure S29. Semi-logarithmic plots for the ROP of *L*-LA catalyzed by formulations ***f1***, ***f2***, ***f3***, and $Sn(Oct)_2$ under industrially relevant conditions. Labels refer to entry numbers in Table S8. 43

Table S9. Conversion and molecular weight data for samples taken during polymerization reaction IE-1 44

Figure S30. Plots of conversion and number average molecular weight, M_n^{GPC}, against time for reaction IE-1..44

Figure S31. A plot of number average molecular weight, M_n^{GPC}, against conversion for reaction IE-1 44

Table S10. Conversion and molecular weight data for samples taken during polymerization reaction IE-2..45

Figure S32. Plots of conversion and number average molecular weight, M_n^{GPC}, against time for reaction IE-2..45

Figure S33. A plot of number average molecular weight, M_n^{GPC}, against conversion for reaction IE-2 45

Table S11. Conversion and molecular weight data for samples taken during polymerization reaction IE-3..46

Figure S34. Plots of conversion and number average molecular weight, M_n^{GPC}, against time for reaction IE-3..46

Figure S35. A plot of number average molecular weight, M_n^{GPC}, against conversion for reaction IE-3 46

Table S12. Conversion and molecular weight data for samples taken during polymerization reaction IE-4..47

Figure S36. Plots of conversion and number average molecular weight, M_n^{GPC}, against time for reaction IE-4..47

Figure S37. A plot of number average molecular weight, M_n^{GPC}, against conversion for reaction IE-4 47

Table S13. Conversion and molecular weight data for samples taken during polymerization reaction IE-5..48

Figure S38. Plots of conversion and number average molecular weight, M_n^{GPC}, against time for reaction IE-5..48

Figure S39. A plot of number average molecular weight, M_n^{GPC}, against conversion for reaction IE-5 48

Table S14. Conversion and molecular weight data for samples taken during polymerization reaction IE-6..49

Figure S40. Plots of conversion and number average molecular weight, M_n^{GPC}, against time for reaction IE-6..49

Figure S41. A plot of number average molecular weight, M_n^{GPC}, against conversion for reaction IE-6. The curve links datapoints chronologically, see Table S14. 49

4 Selected Gel Permeation Chromatograms...50

Figure S42. GPC trace for the polymer product of reaction IR-6; [Zr] = 1.30x10^{-2} mol%, [ROH] = 1.30 mol%, dosed as formulation ***f1***. 50

Figure S43. GPC trace for the polymer product of reaction IR-10; [Zr] = 1.95x10^{-2} mol%, [ROH] = 1.95 mol%, dosed as solid **1** with exogenous BnOH. 50

Figure S44. GPC trace for the polymer product of reaction L-2; [Zr] = 2.60x10^{-2} mol%, [BnOH] = 0.13 mol%, dosed as separate species, solid **1** and exogenous BnOH......................................51

Figure S45. GPC trace for the polymer product of reaction IR-25; [Zr] = 1.3x10^{-2} mol%, [ROH] = 1.30 mol% dosed as formulation ***f1***, further 0.8 mol% aliquots of exogenous BnOH added at 4, 8, 12 and 16 minutes. The corresponding tail to low molecular weight is visible. 51

Figure S46. GPC trace for the polymer product of reaction IE-1, showing a bimodal molecular weight distribution, corresponding to two separate alcohol (and catalyst) addition events. [Zr] = 2.9x10^{-3} mol% + 1.5x10^{-3} mol%, [ROH] = 0.29 mol% + 0.15 mol%, dosed as ***f1*** 52

Figure S47. GPC trace for the sample of the reaction mixture drawn from reaction IE-1 after 90 minutes, showing a monomodal molecular weight distribution, prior to the second alcohol addition event. [Zr] = 2.9x10^{-3} mol% [ROH] = 0.29 mol%, dosed as ***f1*** 52

Figure S48. GPC trace for the polymer product of reaction IE-2. [Zr] = 8.1x10^{-3} mol%, [ROH] = 1.62 mol%, dosed as ***f3*** 53

Figure S49. GPC trace for the polymer product of reaction IE-3. [Zr] = 1.5x10^{-3} mol%, [ROH] = 0.29 mol%, dosed as ***f3*** 53

Figure S50. GPC trace for the polymer product of reaction IE-4. [Zr] = 1.9x10^{-3} mol%, [ROH] = 0.29 mol%, dosed as ***f2*** 54

Figure S51. GPC trace for the polymer product of reaction IE-5. [Zr] = 1.3x10^{-3} mol%, [ROH] = 0.29 mol%, dosed as ***f2*** 54

Figure S52. GPC trace for the polymer product of reaction IE-6, catalyzed by $Sn(Oct)_2$. [Sn] = 5.3x10^{-3} mol%, [Initiator] = 0.29 mol% 55

5 ROP of L-LA under conditions anticipated to promote side-reactions 55

Table S15. Polymerization data for the ROP of *L*-LA in the presence of solid **1** and BnOH 55

5.1 ^{1}H NMR spectra of P*L*LA samples 56

Figure S53. Homonuclear decoupled ^{1}H NMR signal corresponding to the methine protons of the polymer backbone of the P*L*LA product of polymerization L-1 56

Figure S54. Homonuclear decoupled ^{1}H NMR signal corresponding to the methine protons of the polymer backbone of the P*L*LA product of polymerization L-2 57

Figure S55. The methine region of the ^{1}H NMR spectrum of the PLA product of reaction IE-3 (without decoupling). The well-defined quartet at δ_H = 5.17 ppm corresponds to isotactic P*L*LA, with no evidence of epimerization after a 22.5-hour reaction time under industrially relevant conditions. 57

Figure S56. DSC data corresponding to the crystalline P*L*LA product of reaction IE-3. 58

6 Structural data for complex 2, Zr(HL$^{Me/tBu}$)$_2$ 59

Figure S57. Molecular structures of isostructural complexes **1** and **2**. Complex **2** is discussed in the Supporting Information only. 59

6.1 General Procedure 59

Figure S58. The solid-state structure of complex **2**, Zr(HL$^{Me/tBu}$)$_2$. All ellipsoids are shown at the 30 % probability level and H atoms have been omitted, except for those bonded to the N atoms. Selected bond lengths (Å): Zr(1)-O(1) 2.0576(14), Zr(1)-O(2) 2.0490(14), Zr(1)-O(3) 2.0494(13). 60

6.2 Crystallographic parameters for complex 2, Zr(HL$^{Me/tBu}$)$_2$ 60

7 Computational analyses 61

Scheme S2. Energetically inaccessible ligand-dissociation and zirconium benzyl alkoxide formation 62

Scheme S3. Ligand arm-dissociation to form alkoxide complex, 4, and various subsequent mechanistic scenarios 63

Scheme S4. Evaluated mechanistic scenarios for activated monomer-type mechanistic pathways 64

Scheme S5. Energetically inaccessible ligand-dissociation and zirconium benzyl alkoxide formation 66

Figure S59. Energetic profile for the favored mechanism of ROP calculated using the PBE0-D3 protocol 67

Table S16. Transition state energies for the favored mechanism calculated with PBE0-D3, ωB97XD and M06-D3 protocols for comparison 67

Figure S60. Normalized free Gibbs energies of the transition states optimized and computed using various functionals, relative to TS_{IX-X} 68

Table S17. Transition state energies for the favored mechanism calculated with PBE0-D3 at 180 °C and 25 °C 68

Table S18. Computed Gibbs Free Energies at the PBE0-D3/cpcm=ethylacetate/453.15K level of theory for various mechanisms possible for the ring-opening of *L*-lactide catalyzed by **1** and initiated by benzyl alcohol (*vide supra* for layered basis set details) 69

References 71

1 Materials and methods

1.1 Experimental procedures

All manipulations were carried out under a dry argon atmosphere, using standard Schlenk line and glove box techniques, unless otherwise stated. An MBraun glove box was used, with <0.1 ppm H_2O and <10.0 ppm O_2. All dry solvents were drawn from an MBraun solvent purification system and stored over 4 Å molecular sieves.

Zirconium isopropoxide isopropanol complex $[Zr(O^iPr)_4]\cdot(HO^iPr)$ was purchased from Alfa Aesar and used without further purification. All other chemicals were purchased from Sigma Aldrich. Anhydrous benzyl alcohol, BnOH, was degassed under dynamic vacuum for 20 hours prior to use, and stored thereafter under dry argon. *rac*-LA and *L*-LA were recrystallized three times from toluene and then dried under dynamic vacuum for 16 hours, before storage under a dry argon atmosphere. The toluene was drawn directly from the solvent purification system and the recrystallizations carried out under ambient air. After each recrystallization the lactide was filtered over a sintered glass frit and washed sparingly with fresh SPS toluene. All other chemicals were used without further purification.

All NMR spectra were acquired using a 400 MHz (^1H), 101 MHz (^{13}C) Bruker Avance spectrometer, unless otherwise stated. $CDCl_3$ was purchased from Sigma Aldrich and used as received for polymer analysis. For analysis of metal complexes, $CDCl_3$ was dried over calcium hydride, distilled under vacuum, and stored over 4 Å molecular sieves under a dry argon atmosphere. Toluene-d_8 was purchased from Sigma Aldrich and stored over 4 Å molecular sieves under a dry argon atmosphere. Processing was carried out using Mestrelab Research MestReNova version 11.0.2-18153.

Crystallographic data was collected using a Supernova EOS detector diffractometer with Cu-Kα radiation (λ=14184 Å) at 150(2) K. Structures were solved using direct methods and refined on all F^2 data using the SHELXL-97 suite of programs. All hydrogen atoms were included in idealised positions and refined using the Riding model.

Polymer molecular weight data was acquired using an Agilent 1260 Gel permeation Chromatography (GPC) system equipped with triple detection (differential refractive index detector, viscometer and dual angle light scattering detector (90°/15°, only 90° data was used). A PLgel 5 µm MIXED-D 300 x 7.5 mm column was used, with a PLgel 5 µm MIXED Guard 50 x 7.5 mm guard column. The mobile phase was THF, at a flow rate of 1 ml min^{-1}. Columns and detectors were maintained at 35 °C. Data was processed using Agilent's GPC/SEC Software, Revision A.02.01. Unless otherwise stated, polymer samples were not purified prior to GPC analysis, and molecular weight values have been adjusted according to the final percentage conversion, as determined by ^1H NMR spectroscopy.

Thermogravimetric analysis was carried out using a Setaram Setsys Evolution TGA 16/18, equipped with a 170 µl alumina crucible. Samples of mass ~20 mg were heated to 1000 °C at a rate of 10 K min^{-1}, under a flow of dry argon. The furnace was purged with dry argon for 40 minutes prior to use, at a flow rate of 200 ml min^{-1}. Data was processed using the Calisto software package (version 1.41).

Mass spectrometry was carried out with a Bruker Daltonik microToFelectrospray time-of-flight (ESI-ToF) mass spectrometer. Samples were dissolved in methanol at a concentration of 10 µg mL^{-1}, and positive ionisation mode was used.

In-situ Attenuated Total Reflectance Fourier-Transform Infra-Red (ATR-FT-IR) Spectroscopic reaction monitoring of polymerization reactions was carried out using a Bruker Matrix-MF spectrometer, fitted with a IN350-T fibre-optic, diamond insertion probe. Spectroscopic data was processed using Bruker's OPUS 7.5 software package, and Microsoft Excel 2013. Temperature control was achieved using a Huber Petite Fleur temperature control system. (For further experimental details see general procedure described in **1.5.1**).

1.2 Synthetic procedures and analytical data

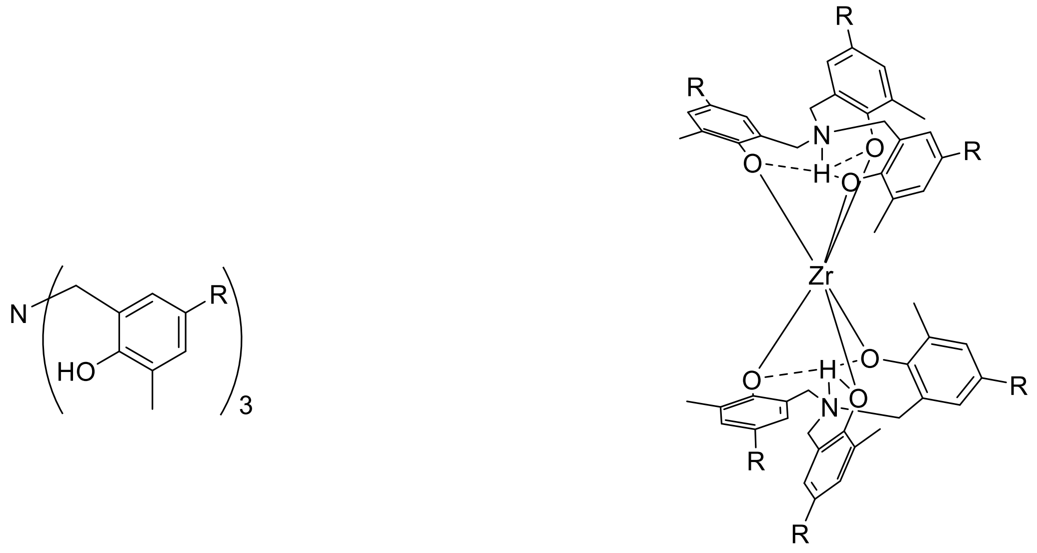

H_3L^{Me}: R=Me
$H_3L^{Me/tBu}$: R=^tBu

$Zr(HL^{Me})_2$, **1** : R=Me
$Zr(HL^{Me/tBu})_2$, **2**: R=^tBu

Figure S1. Pro-ligands H_3L^{Me}, $H_3L^{Me/tBu}$, and zwitterionic complexes $Zr(HL^{Me})_2$, **1**; and $Zr(HL^{Me/tBu})_2$, **2**

Synthesis of pro-ligand tris(2-hydroxy-3,5-dimethylbenzyl)amine, H_3L^{Me}

Pro-ligand H_3L^{Me} was synthesised according to the following adapted literature procedure.[1] To 818 mmol (100 g) 2,4-dimethylphenol was added 68.18 mmol (9.5 g) hexamethylenetetramine, 545 mmol (16.4 g) paraformaldehyde, and 2.72 mol (50 ml) of deionised water. The mixture was then refluxed with vigorous stirring for 120 hours in an oil bath at 150 °C, with two further 10 ml aliquots of 2,4-dimethylphenol added after 72 hours and 90 hours respectively. The reaction mixture was cooled and the resulting orange solid washed over a glass frit with 5 x 400 ml MeOH, to yield a white powder. The powder was then dried under dynamic vacuum for 24 hours. ^1H and $^{13}C\{^1H\}$ NMR data in $CDCl_3$ was in agreement with the literature.[1] *Yield: 63 g, 55 %*

Synthesis of pro-ligand tris(2-hydroxy-3-methyl-5-*tert*-butylbenzyl)amine, $H_3L^{Me/tBu}$

Pro-ligand $H_3L^{Me/tBu}$ was synthesised according to the following adapted literature procedure.[2] To 85 mmol (9.6 g) 2-methyl-4-*tert*-butylphenol was added 4.9 mmol (0.68 g) hexamethylenetetramine, 38.9 mmol (1.17 g) paraformaldehyde, and 200 mmol (3.7 ml) of deionised water. The mixture was then refluxed with vigorous stirring for 120 hours in an oil bath at 120 °C, with two further 2.2 ml aliquots of 2-methyl-4-*tert*-butylphenol added after 72 hours and 90 hours respectively. The reaction mixture was cooled and the resulting yellow solid dissolved in methanol and precipitated by addition of water. The precipitate was isolated by gravity filtration (filter paper) and air dried, before recrystallization from hexane and drying under dynamic vacuum, yielding a yellow solid. ^1H and $^{13}C\{^1H\}$ NMR data in $CDCl_3$ was in agreement with the literature.[2] *Yield: 2.3 g, 22 %*

Synthesis of zirconium amine tris(2-hydroxy-3,5-dimethylphenolate) complex, $Zr(HL^{Me})_2$, 1.

1 was synthesised by adapted literature procedure. 24.5 mmol (10.3 g) H_3L^{Me}, was added to 200 ml of dry toluene. To the resulting stirred suspension was added a solution of 12.3 mmol (4.7 g) $[Zr(O^iPr)_4]\cdot(HO^iPr)$ in minimum dry toluene (~20 ml). The reaction mixture was heated until a clear yellow-brown solution was observed. Precipitation of a white solid was observed on removal of the heat source. The mixture was allowed to cool to room temperature, and the solid isolated by cannula filtration and washed with 70 ml dry toluene. The air- and moisture-stable solid product was then dried under dynamic vacuum for 16 hours. Further purification was carried out where appropriate by recrystallization from toluene. ^1H and $^{13}C\{^1H\}$ NMR data for **1** in $CDCl_3$ was in agreement with the literature.[3] *Yield: 7.5 g, 66 %*

Synthesis of zirconium amine tris(2-hydroxy-3-methyl-5-*tert*-butyl) complex, $Zr(HL^{Me/tBu})_2$, 2.

2 was prepared by an analogous procedure to **1**. The air- and moisture-stable product was not recrystallized. ^1H and $^{13}C\{^1H\}$ NMR data for **2** in $CDCl_3$ was in agreement with the literature.[2] The crystal structure of **2** is reported for the first time in the current work (see below). *Yield: 1.5 g, 63 %*

1.3 Physical properties of complexes 1 and 2

Solubility of catalyst 1 in toluene and o-xylene

For industrial relevance (economy, sustainability and safety), ambient-temperature storage and delivery of **1** in concentrated solution is desirable, ensuring compatibility with current infrastructure. The solubility of **1** in high-boiling, industrially relevant, aprotic solvents was investigated. Using variable-temperature ^1H NMR spectroscopy, the solubility of **1** at various temperatures in toluene-d_8 and *o*-xylene-d_{10} was assessed, by comparison with a 1,3,5-trimethoxybenzene standard.

In each case, a stock solution was prepared at 100 °C, of 33.4 mg of 1,3,5-trimethoxybenzene in 2 ml toluene- d_8, or 11.7 mg of 1,3,5-trimethoxybenzene in 1.7 ml *o*-xylene-d_{10}. To this, **1** was added stepwise until saturation was reached (23.8 mg was added to toluene, corresponding to a Zr concentration of ~1.3×10^{-2} mol dm^{-3}, or 1.2 g L^{-1}, and 23.4 mg to *o*-xylene, corresponding to ~1.5×10^{-2} mol dm^{-3}, or 1.4 g L^{-1}). 0.6 ml of the relevant saturated solution was transferred to a J Young's NMR tube, and sealed under air. After cooling to –78 °C, the solution was left at ambient temperature for 20 hours, allowing precipitation of **1**. ^1H NMR spectra were acquired at a range of ascending temperatures. Prior to each acquisition, the temperature was increased to the required value and maintained for 5 minutes. The sample was then removed from the spectrometer, shaken vigorously and returned,

whereupon the temperature was maintained for a further five minutes prior to data acquisition. 1 ml of toluene was found to dissolve at least 1 g of 1,3,5-trimethoxybenzene at ambient temperature (20 °C). Accordingly, in the course of NMR solubility studies, the 1,3,5-trimethoxybenzene internal standard was assumed to be always fully dissolved. The concentration of **1** determined by comparison of integrated methyl ^{1}H resonances corresponding to both species.

The solubility of **1** in toluene-d_8 reached the maximum value at ~70 °C (Figure S2), and under the experimental conditions, the solubility of **1** in *o*-xylene-d_{10} reached a plateau at ~80 °C (Figure S3), corresponding to a Zr concentration of ~1.3x10^{-2} mol dm^{-3} (~1.2 g L^{-1}).

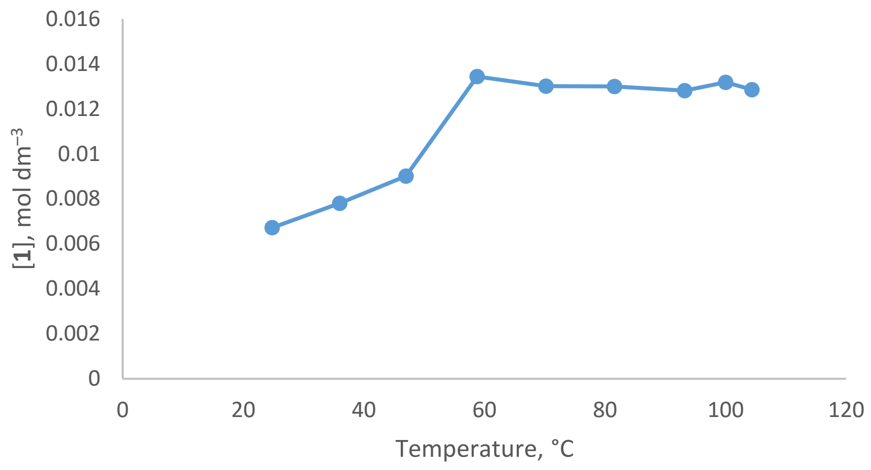

Figure S2. Solubility curve for catalyst **1** in toluene-d_8, produced using variable-temperature NMR spectroscopy

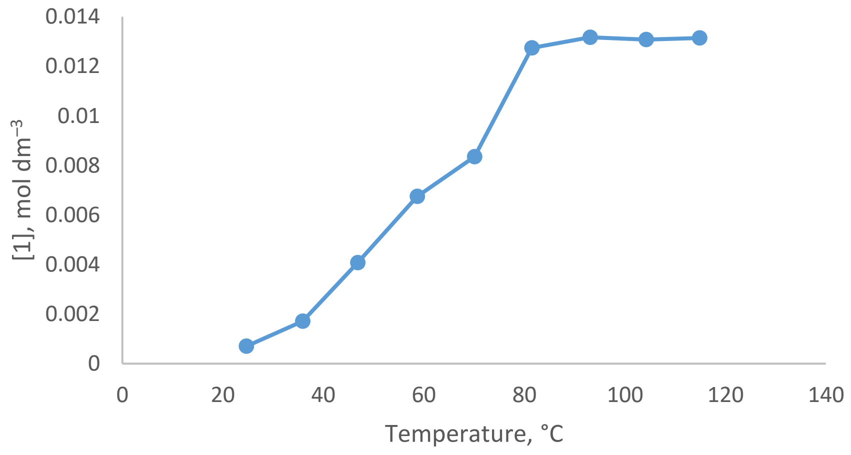

Figure S3. Solubility curve for catalyst **1** in *o*-xylene-d_{10}, produced using variable-temperature NMR spectroscopy

The calculated solubility of **1** in toluene-d_8 was corroborated by solvation of **1** in *protio*-toluene on a larger scale. At 70 °C, 15 ml of *protio*-toluene was required to solvate 120 mg of **1**. This corresponded to a Zr concentration of ~0.8 g L^{-1}.

The discrepancy between the measured maximum solubility of **1** obtained using the two methods is likely due to experimental errors regarding the concentration of the 1,3,5-trimethoxybenzene standard during the NMR study, and evaporation of solvent during the larger-scale experiment. However, the solubility in both cases was much too low to be industrially useful, and so further measurements were not made.

Rapid solubility screening of 1 in industrially relevant solvents

Other solvent systems were screened, in each case by dropwise addition of the relevant solvent to 100 mg of **1**. Where solubility was lower than that of toluene and *o*-xylene (1.2 g L^{-1}), the experiment was terminated. For industrial use, a Zr concentration of 3.5 g L^{-1} was targeted. Solubility was insufficient in all cases for industrial application (Table S1), with the exception of benzyl alcohol. However, extensive heating was required to solubilise **1** in BnOH (120 mins at 180 °C), and it did not remain fully solvated on cooling to ambient temperature.

Solubility screening for each solvent was undertaken in a 30 ml vial, held in an aluminium block heated to the required temperature. The mass of solvent added to fully dissolve the solid material allowed determination of the concentration of **1**.

Table S1. Solubility values of **1** in a range of solvents from crude solvent screening

Solvent	Temperature/ °C	Solubility of **1**, g L^{-1} [Zr]
Toluene	70	0.8-1.2
Xylene	80	~1.2
Acetonitrile	50	<1.2
Tetrahydrofuran (THF)	50	<1.2
2-methyl THF	50	<1.2
Propylene carbonate	170	~1.7
N,*N'*-dimethylpropyleneurea (DMPU)	100	~2.5
2-butanone	50	<1.2
Benzyl alcohol (BnOH) (under air-free conditions)	180	≥8.5

Solubility of complex 2

It was anticipated that the presence of *tert*-butyl groups on the ligand framework of **2** would enhance solubility in hydrocarbon solvents, relative to **1.** However, **2** was much less soluble in toluene than **1** (~0.33 g L^{-1}, $2.90x10^{-1}$ g L^{-1} at 30 °C, decreasing with increased temperature).

Thermal stability of complexes 1 and 2

Thermogravimetric analysis showed that complex **2** was more thermally stable than complex **1**. **1** decomposed in the temperature range 226-281 °C when heated from ambient temperature at 10 K min^{-1}, whereas **2** decomposed in the range 245-327 °C (inflection temperature = 247 °C and 287 °C, respectively; Figure S4), suggesting there would be an increased activation barrier for the ROP of LA in the presence of **2**, relative to **1**, *via* the proposed mechanism. Accordingly, the catalytic use of **2** was not investigated further. Notably, **1** did not undergo any decomposition event below 226 °C, suggesting that ligand dissociation does not spontaneously occur at the temperature used for the solvent-free ROP of lactides in the current work (≤180 °C), although this, alone, does not discount ligand dissociation occurring more readily in the presence of the monomer and/or co-initiator.

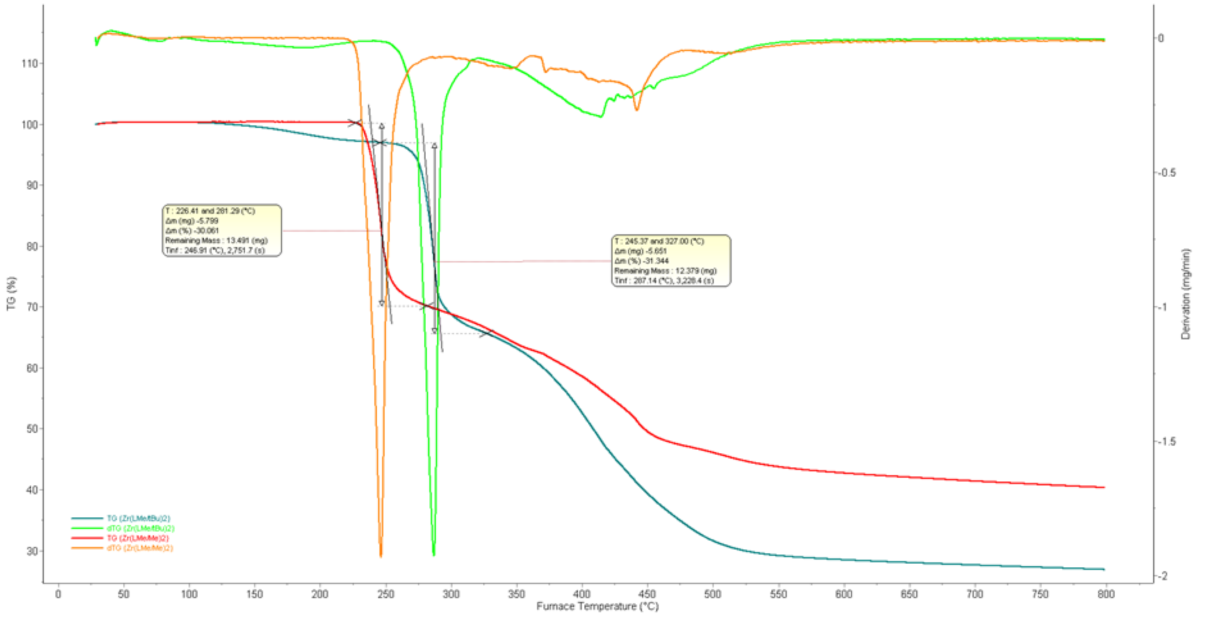

Figure S4. TG and dTG curves corresponding to the thermal decomposition of **1** (TG shown in red, dTG in orange) and **2** (TG shown in blue, dTG in green) under an argon atmosphere

1.4 Preparation of catalyst formulations

1.4.1 Preparation of *f1*

In the glove box, a J Young's ampoule was charged with 0.347 mmol (0.32 g) of zirconium complex **1**, 34.7 mmol (5 g) of *rac*-LA and 34.7 mmol (3.6 ml) of BnOH. The ampoule was then sealed, removed from the glove box, and heated in an oil bath with vigorous stirring, to 180 °C for 50 minutes, until a clear yellow solution, ***f1***, was obtained. ***f1*** remained liquid on cooling to ambient temperature. The major component of ***f1*** was anticipated to be the racemate of diester $PhCH_2OC(O)CH(CH_3)OC(O)CH(CH_3)OH$, ***e1***; the product of ring-opening one equivalent of LA in the presence of an immortal polymerization catalyst (Zr complex, **1**) and a BnOH nucleophile (Scheme S1). The fast initiation step and slower propagation characteristic of an immortal regime was expected to favor this species as the major component. Mass spectrometry revealed the presence of ***e1***, as well as heavier lactyl oligomers (up to 7 lactic acid units, all detected as the corresponding Na^+ adducts) in ***f1***.

The repeat unit of the oligomers, observed in the mass spectrum, was 72 g mol^{-1}, corresponding to a single lactic acid unit, rather than the dimeric LA monomer (Figure S5). Thus, the presence of heavier oligomers was attributed to transesterification of ***e1*** in the presence of a high concentration of complex **1** during formulation, rather than any significant non-living character with respect to polymerization catalyst **1**. Oligomers with a 72 g mol^{-1} repeat unit comprising the co-initiating species in formulation ***f1*** precludes use of MALDI-TOF-MS analysis of polymer products to identify or quantify any transesterification activity occurring during catalytic application of ***f1*** to the ROP of lactides. The presence of diester ***e1*** and heavier lactyl oligomers was supported by ^{1}H and ^{13}C{^{1}H} NMR analysis of ***f1*** (Figure S6). The concentration of Zr, introduced as catalyst **1**, in ***f1*** was experimentally determined to be 4.27x10^{-2} mol dm^{-3} (3.90 g L^{-1}) corresponding to 1 mol% with respect to the total number of benzyl residues, or alcohol groups present. This value was 2.847 x10^{-2} mol dm^{-3} and 2.135 x10^{-2} mol dm^{-3} for ***f2*** and ***f3***, respectively, used under industrial conditions to calculate ppm Zr catalyst loadings, see relevant section below. Catalyst degradation during formulation, the potential for error associated with the method used to determine $[Zr]_{f1}$, and other measurement errors and variables arising from the formulation step make these values approximations. Nonetheless, they are used consistently throughout the current work, allowing valid investigation of the system for industrial application, and are supported by good agreement between theoretical and experimental polymer molecular weights. Although a solution of equivalent molar concentration of catalyst **1** can be prepared in neat BnOH, that system appeared to be less stable than ***f1***, precipitating solid catalyst particles within one day on cooling to ambient temperature. This is incompatible with large-scale preparation and storage.

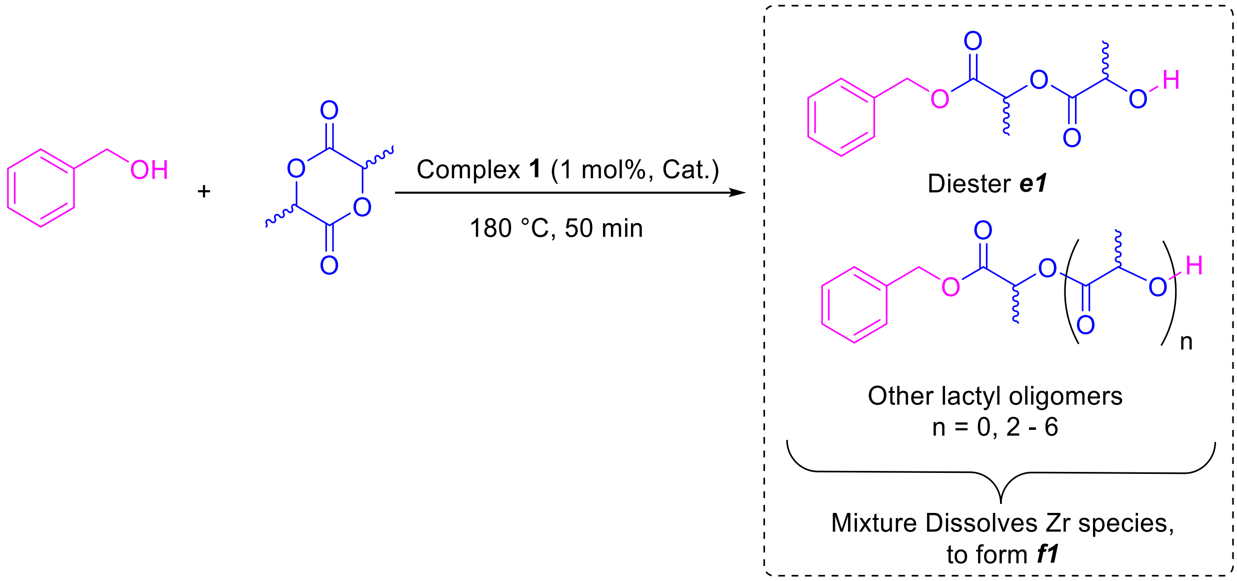

Scheme S1. Diester ***e1***, the product of the ring-opening of one equivalent of lactide in the presence of a benzyl alcohol nucleophile, and heavier oligomers arising from transesterification of ***e1***, comprising the solvent system of catalyst formulation ***f1***

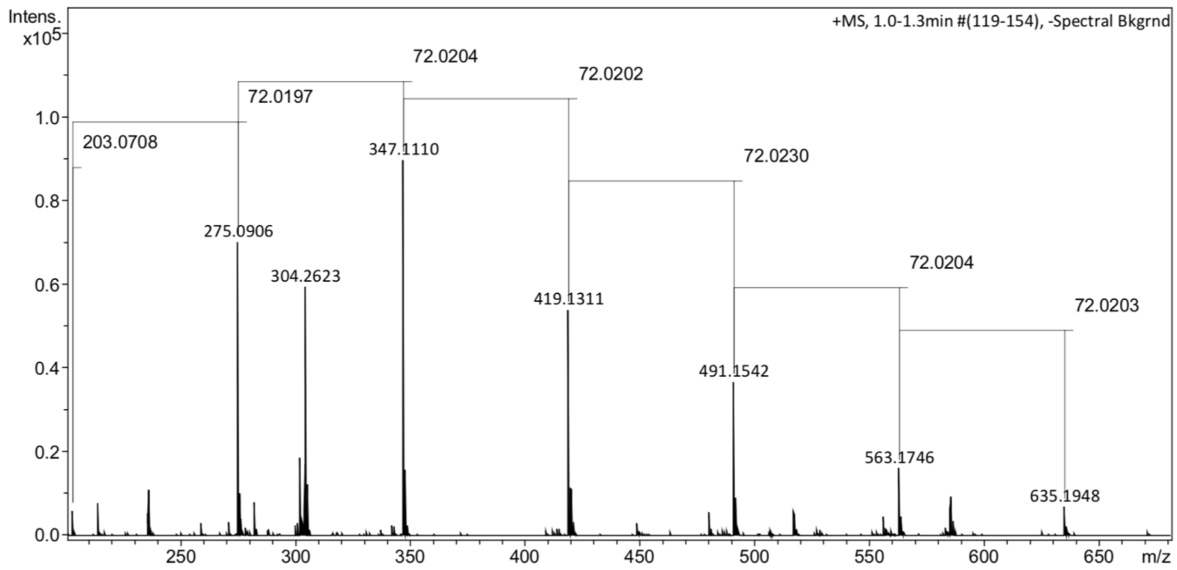

Figure S5. The mass spectrum of catalyst formulation ***f1***, showing lactyl oligomers of up to seven lactic acid repeat units. All are observed as the corresponding Na^+ adducts

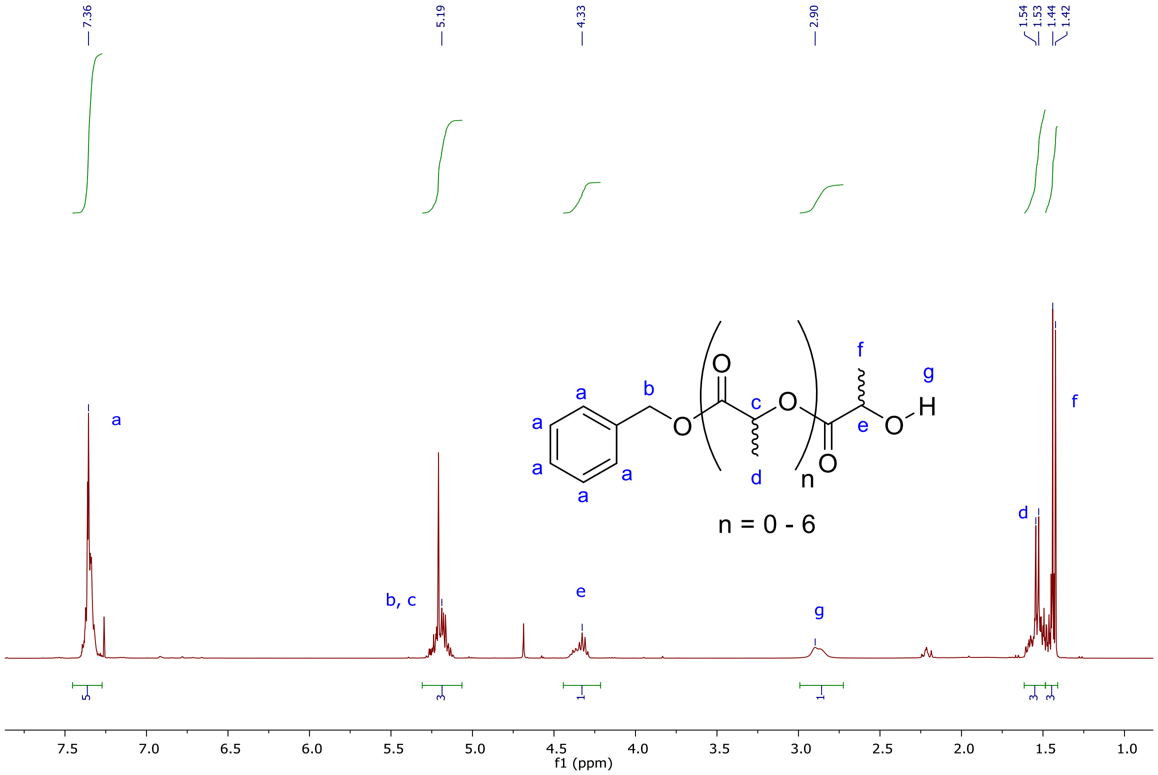

Figure S6. The ^{1}H NMR spectrum of catalyst formulation ***f1*** (400 MHz, $CDCl_3$, 298 K), consistent with the presence of benzyl ester ***e1*** and heavier lactyl oligomers. Signal at 7.26 ppm is residual $CHCl_3$.

^{1}H NMR (400 MHz, $CDCl_3$, 298 K δ_H, ppm); 7.30 (5H, *m*, Ar), 5.16 (3H, *m*, ArCH_2 and C(O)C*H*(Me)OC(O)), 4.28 (1H, *m*, C*H*(Me)OH), 2.85 (1H, *s*, O*H*), 1.48 (3H, *d*, *J*=7.1 Hz, CH_3COC(O)), 1.38 (3H, *d*, *J*=6.9 Hz, CH_3COH). **^{13}C{^{1}H} NMR** (101 MHz, $CDCl_3$, 298 K δ_c, ppm); 175.42 (C=O), 174.99 (C=O), 135.20 (*Ar*CH_2), 128.58 (Ar), 128.45 Ar), 128.16 (Ar), 128.09 (Ar), 69.30 (*C*H_2), 67.20 (C(O)*C*H(Me)OC(O)), 66.78 (*C*H(Me)OH),), 20.29 (*C*H_3), 16.70 (*C*H_3).

Assignation of resonances was compromised due to overlap of many methyl and methylene signals corresponding to various chiral oligomeric lactyl species. The appearance of complex multiplets in the methylene and methyl regions of the ^{1}H spectrum indicates racemic enchainment of L-lactic acid and D-lactic acid residues arising due to transesterification.

In addition to an increase in the concentration of **1** when compared to toluene, use of ***f1*** does not introduce any exogenous solvent to the polymerization reaction mixture. The solvent system of ***f1***, generated *in-situ*, serves as the external nucleophile (co-initiator) in the ROP of LA, thus forming part of the polymer chain. This enhances industrial relevance, and ensures ease of kinetic analysis. Additionally, **1** remains solvated in ***f1*** at ambient temperature for at least 18 months, increasing ease of handling and storage when compared to traditional solvent systems. Although the components of formulation ***f1*** are air- and moisture-stable, the catalyst formulation was handled under a dry argon atmosphere at all times. This was to prevent contamination with protic impurities (moisture), which may negatively affect molecular weight control, prior to catalytic use.

1.4.2 Preparation of *f2* and *f3*

Catalyst formulations ***f2*** and ***f3*** were prepared analogously to ***f1***. However, ***f2*** and ***f3*** contained 0.67 mol% and 0.5 mol% catalyst **1**, respectively, and required 35 minutes and 30 minutes of heating, respectively (Table S2).

Table S2. Molar composition of catalyst formulations ***f1***, ***f2***, ***f3***, and heating time required for preparation of each formulation

Formulation	[**1**], relative molar composition	[LA], relative molar composition	[BnOH], relative molar composition	Heating time, mins
f1	1	100	100	50
f2	1	150	150	35
f3	1	200	200	30

1.5 *In-situ* ATR-FT-IR-monitored polymerization of *rac*-LA or *L*-LA

1.5.1 General procedure

A three-necked, jacketed glass reactor was loaded with 20 g of *rac*- or *L*-LA as required. A mechanical stirrer and Bruker IN350-T ATR-FT-IR insertion probe were respectively sealed into two of the vessel's necks. The third (central) neck of the reaction vessel was connected to a Schlenk line (Figure S7).

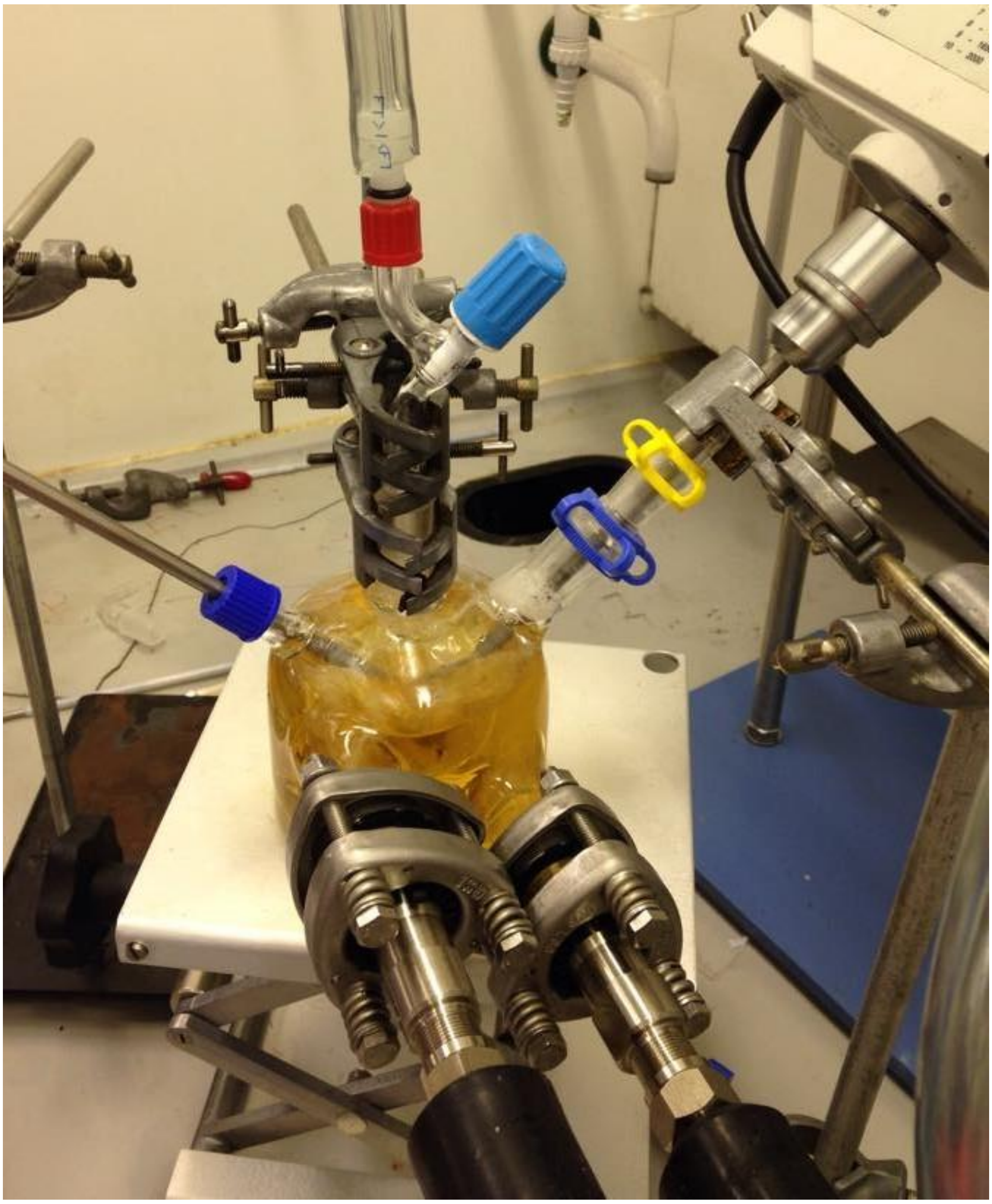

Figure S7. A photograph of the jacketed glass reactor used for ATR-FT-IR-monitored polymerization reactions. ATR-FT-IR insertion probe (left), Schlenk line attachment (center) and mechanical stirrer (right) are *in-situ*. Heat transfer fluid was passed through the outer jacket *via* insulated tubing (foreground)

The charged reactor was placed under dynamic vacuum (0.03 mbar) for sixty minutes, then refilled with dry argon at ambient temperature. The vessel was evacuated for five minutes, and refilled in the same manner a further two times, then sealed and heated, with stirring to 174 °C (unless otherwise stated), this temperature being determined by the operating capabilities of the experimental apparatus (a thermostat setting of 180 °C for the thermal fluid achieved a lactide melt temperature of 174 °C). Heating was *via* passage of a heat transfer fluid (silicone oil) in a closed circuit from a Huber Petite Fleur temperature control system, through the outer jacket of the reactor. Once the vessel reached 174 °C, it was briefly opened to ambient air by removal of the Schlenk line connection, and the catalyst formulation, ***f1***, was added by syringe, having been handled under dry argon using Schlenk techniques. Where solid catalyst **1** was used, it was tipped directly from a polystyrene weighing boat into the open reaction vessel. BnOH was added by syringe where required. The vessel was immediately sealed after addition of catalyst (and co-initiator, where required) and the reaction allowed to proceed.

Concurrently with addition of the catalyst, collection of real-time ATR-FT-IR data commenced. Quantitation of LA and PLA concentrations was achieved by integration of the ATR-FT-IR signals corresponding to the C-O-C stretching mode, at wavenumber regions 1203-1265 cm^{-1} and 1160-1200 cm^{-1}, respectively (Figure S8, Figure S9). Integration was carried out between points on the curve corresponding to the relevant wavenumbers (Figure S10). Further data processing and construction of semi-logarithmic plots was carried out using Microsoft Excel 2013.

The polymer product produced in the course of a given ATR-FT-IR-monitored experiment was immediately poured from the reactor on cessation of ATR-FT-IR data acquisition, and allowed to cool under ambient conditions without further purification. The final percentage conversion was determined by integration of the methine region of the product mixture's ^{1}H NMR spectrum, in $CDCl_3$. Resonances at δ_H = 5.0-5.07 ppm and δ_H = 5.08-5.25 ppm are characteristic of the monomer and polymer species, respectively. Analysis of molecular weight was carried out *via* Gel Permeation Chromatography with Triple Detection. Where applicable, the tacticity of the polymer product was assessed *via* ^{1}H NMR spectroscopy, with homonuclear decoupling of the polymer methine signal (see below).[4] Concentration data were converted from mol% to mol dm^{-3} where required based on a density of 1.3 g cm^{-3} for the molten reaction mixture at 174 °C, irrespective of conversion. This value was experimentally determined using as the density of pure molten LA at 174 °C.

For Variable Time Normalisation Analysis, VTNA,[5,6] LA concentration was plotted against a time axis that was normalized by multiplying by catalyst concentration (raised to the power 1), giving [LA] *versus* [Zr]t. Data from all experiments were plotted on the same axes, with superimposition of the resulting curves indicative of a first order rate dependence on catalyst formulation ***f1***, and therefore on catalyst **1** for an immortal ROP. For simplicity of VTNA, the concentration of LA was normalized such that at time t = 0 the value was exactly 100 in all cases.

Initial rate analysis was carried out by constructing semi-logarithmic initial rate plots, the ROP exhibiting pseudo-first order rate dependency with respect to the LA monomer. Using the concentration of the monomer to monitor reaction progress, $\ln([LA]_0/[LA]_t)$ was plotted against time, t.

Where indicated, the stereoselectivity of catalytic systems was calculated using both kinetic methods and polymer characterisation data. The former method used the relative rate constants, $k_{obs(L\text{-}LA)}$ and $k_{obs(rac\text{-}LA)}$, for the ROP of *L*-LA and *rac*-LA, respectively, using the method described by Nomura and co-workers.[7] Accordingly, polymer tacticity is given by: P_r=1-(0.5($k_{obs(L\text{-}LA)}/k_{obs(rac\text{-}LA)}$)), where P_r is the probability of heterotactic enchainment.[7] In the latter case, the tacticity of polymer samples was determined *via* homonuclear-decoupled ^{1}H NMR (decoupling of the lactyl methine proton signal from the adjacent methyl group). P_r was calculated using the equation $P_r=\sqrt{(2[sis])}$, where [*sis*] corresponds to the number of methine protons in the *sis* tetrad as a fraction of the total number of methine protons, according to Bernouillian statistics, as described by Coates.[4]

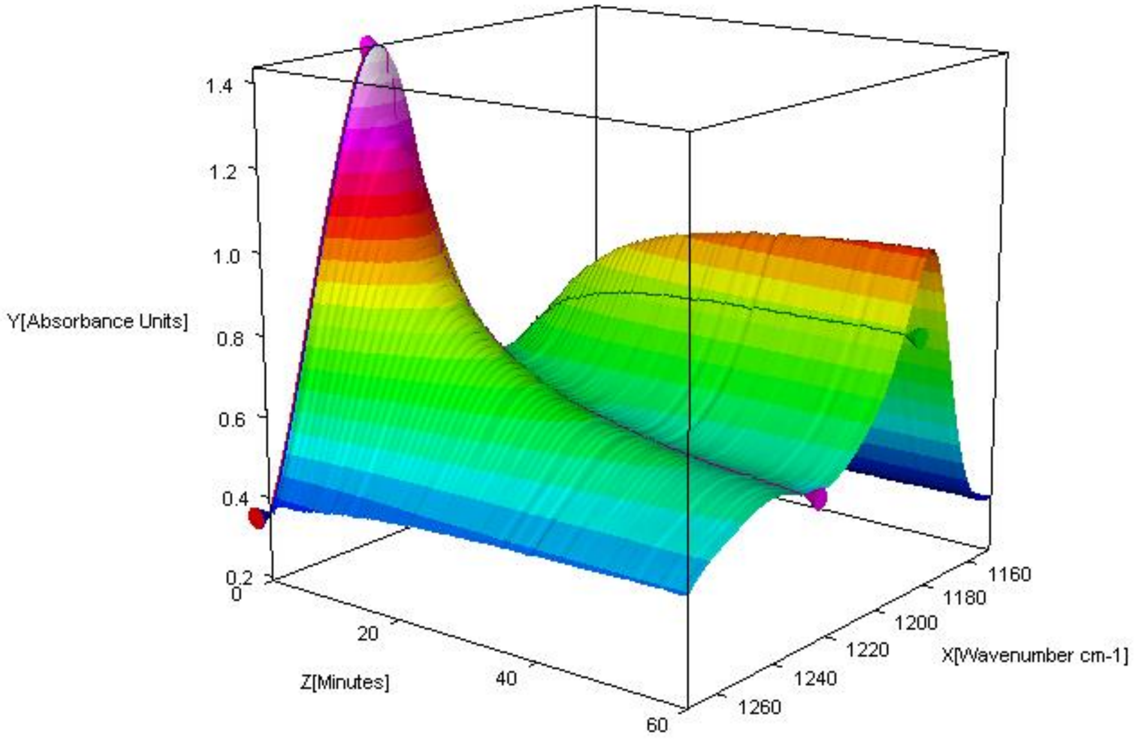

Figure S8. Example three-dimensional plot showing change of *rac*-LA and PLA ATR-FT-IR signals spectra corresponding to the solvent-free ROP of *rac*-LA in the presence of catalyst formulation ***f1***

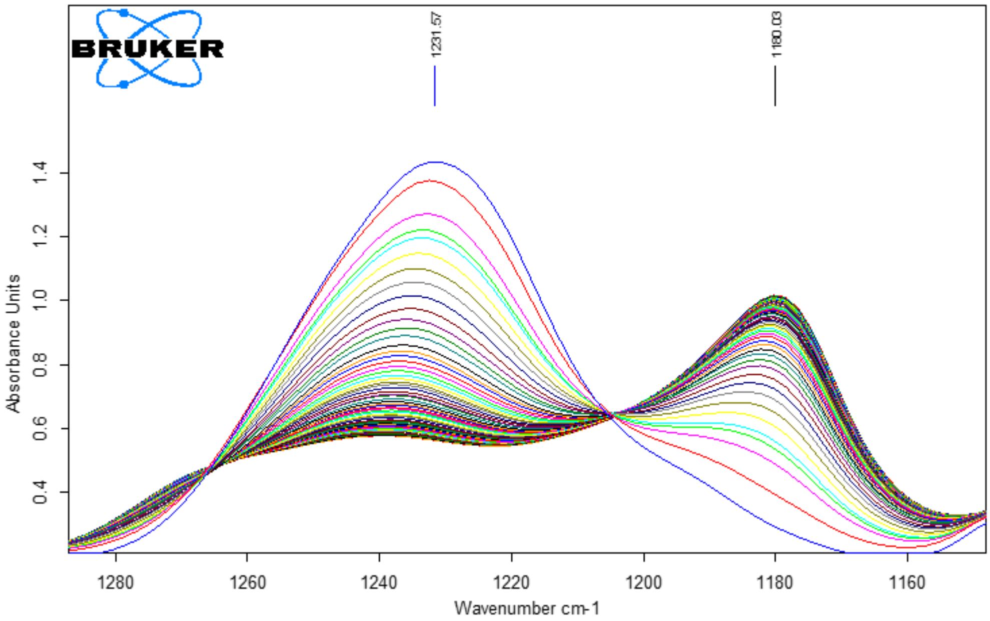

Figure S9. Two-Dimensional ATR-FT-IR spectra corresponding to the solvent-free ROP of *rac*-LA in the presence of catalyst formulation ***f1***; lactide and poly(lactic acid) signals corresponding to the C-O-C stretching mode appear at wavenumber regions 1203-1265 cm^{-1} and 1160-1200 cm^{-1}, respectively

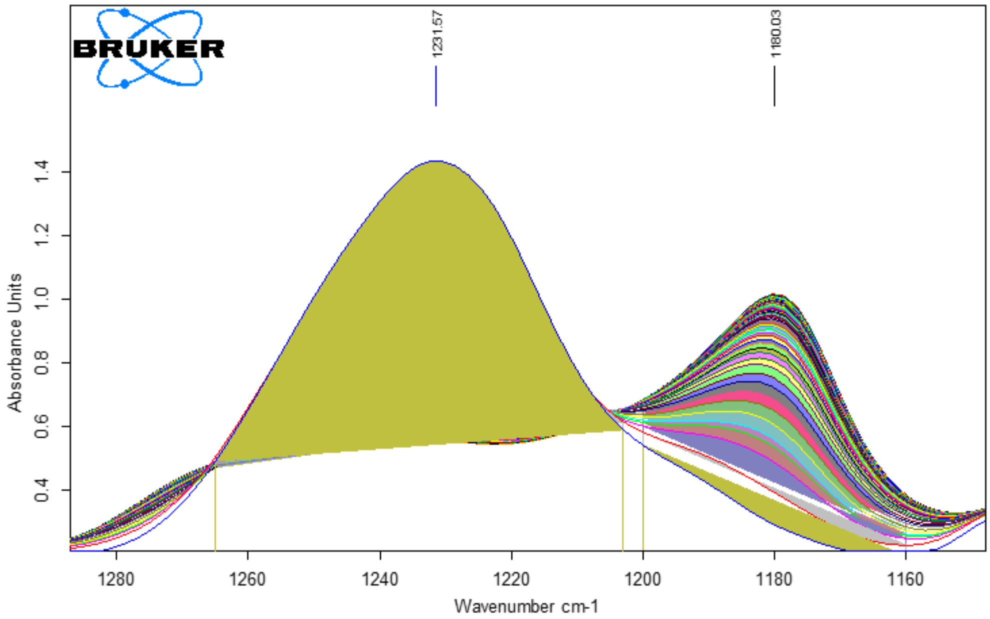

Figure S10. Two-Dimensional ATR-FT-IR spectra corresponding to the solvent-free ROP of *rac*-LA in the presence of catalyst formulation ***f1*** with visualised signal integration method; lactide and poly(lactic acid) signals corresponding to the C-O-C stretching mode appear at wavenumber regions 1203-1265 cm^{-1} and 1160-1200 cm^{-1}, respectively

1.5.2 Calibration of ATR-FT-IR reaction monitoring apparatus

The *in-situ* ATR-FT-IR reaction monitoring apparatus was calibrated using sample mixtures of PLA and *rac*-LA, of known molar composition, with respect to the number of lactyl groups (PLA:LA = 0:100, 20:80, 40:60, 60:40, 80:20, and 100:0, respectively). Following acquisition at 174°C of ATR-FT-IR spectra corresponding to each sample, a calibration curve was constructed, describing the relationship between the integrated area of the LA signal and the concentration of LA (Figure S11).

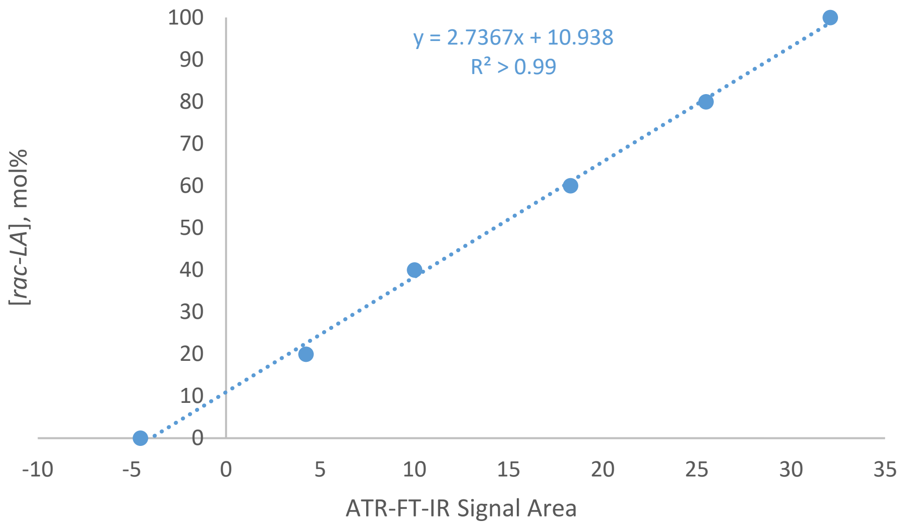

Figure S11. Calibration curve for determination of *rac*-LA concentration from integrated ATR-FT-IR signal area at 174 °C

Accordingly, the linear relationship between the integrated area of the *rac*-LA signal (1203-1265 cm^{-1}) and *rac*-LA concentration (mol%), at 174 °C is described by Equation S1, where *a* = integrated LA signal area.

$$[lactide] = 2.7367a + 10.938$$

Equation S1. Equation describing the relationship between integrated lactide ATR-FT-IR signal area and concentration

Calibration curves were constructed for several temperatures (174 °C, 164 °C, 154 °C, 144 °C). However, any temperature dependence of the ATR-FT-IR LA signal area was shown to be negligible (Figure S12, Figure S13). Thus, for simplicity, ATR-FT-IR data obtained at all temperatures (determination of $\Delta G^{\ddagger}$) was processed using the calibration curve corresponding to a reaction temperature of 174 °C. Additionally, the calibration data obtained using *rac*-LA was assumed to be valid for manipulation of kinetic data corresponding to the polymerization of *L*-LA.

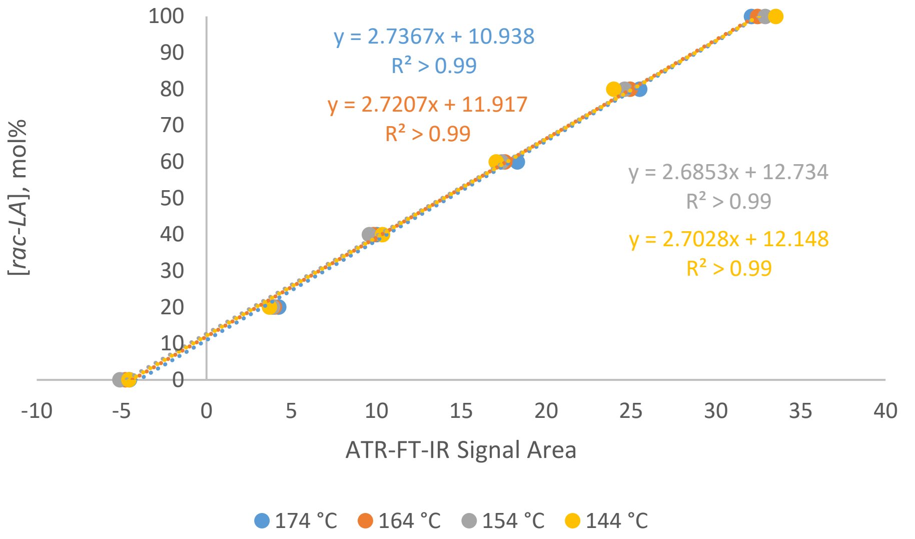

Figure S12. Calibration curves for determination of *rac*-LA concentration from integrated ATR-FT-IR signal area at various temperatures

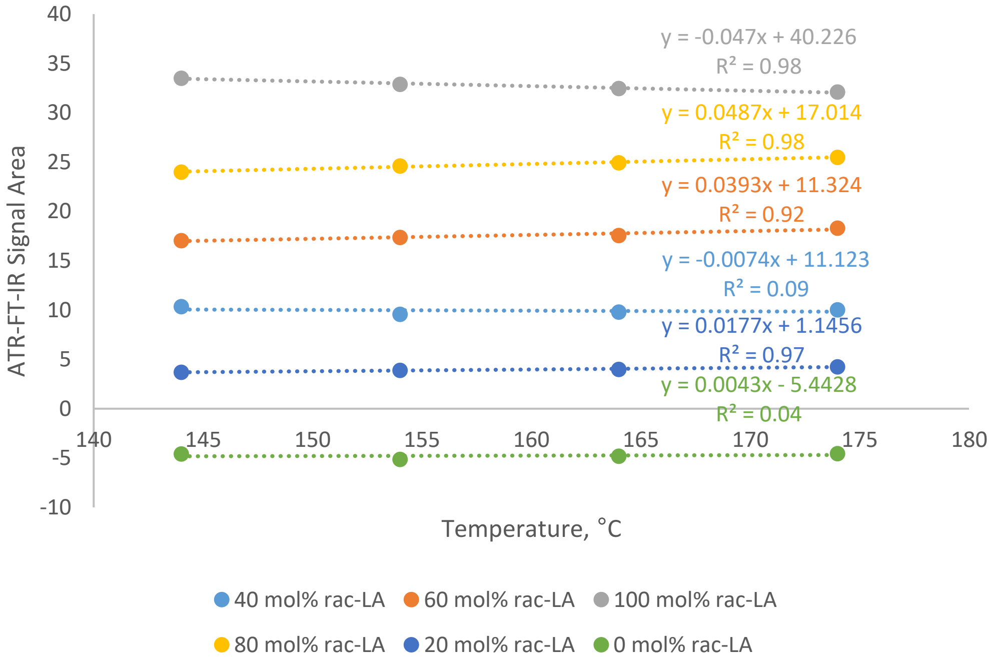

Figure S13. Variation of integrated *rac*-LA ATR-FT-IR signal area with temperature for *rac*-LA/ PLA mixtures of various composition

Calibration of the PLA signal (1160-1200 cm^{-1}) using the same method (Figure S14) showed that the relationship between the concentration of PLA and the integrated area of the corresponding ATR-FT-IR signal was described by the second order polynomial given in Equation S2, where b = integrated PLA signal area. Similarly, integrated signal area showed no significant temperature dependence (Figure 14, Figure 15).

$$[PLA] = 0.2267b^2 + 5.236b + 9.4644$$

Equation S2. Equation describing the relationship between integrated lactide ATR-FT-IR signal area and concentration

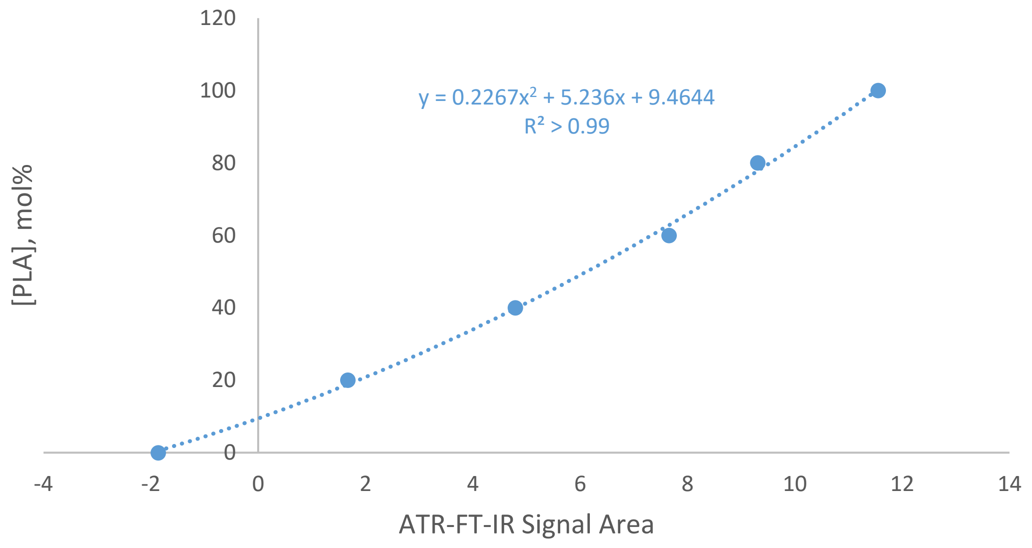

Figure S14. Calibration curve for determination of PLA concentration from integrated ATR-FT-IR signal area at 174 °C

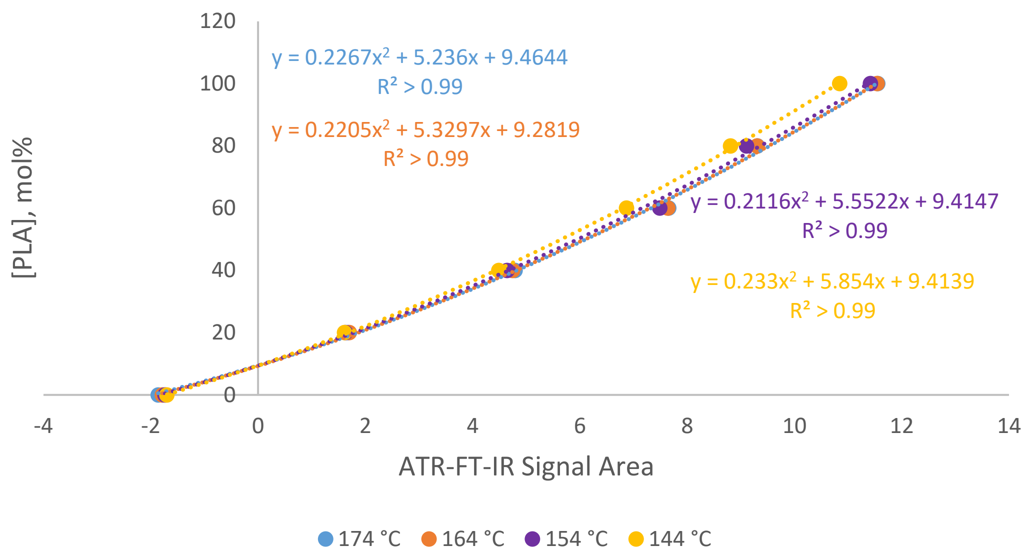

Figure S15. Calibration curves for determination of PLA concentration from integrated ATR-FT-IR signal area at various temperatures

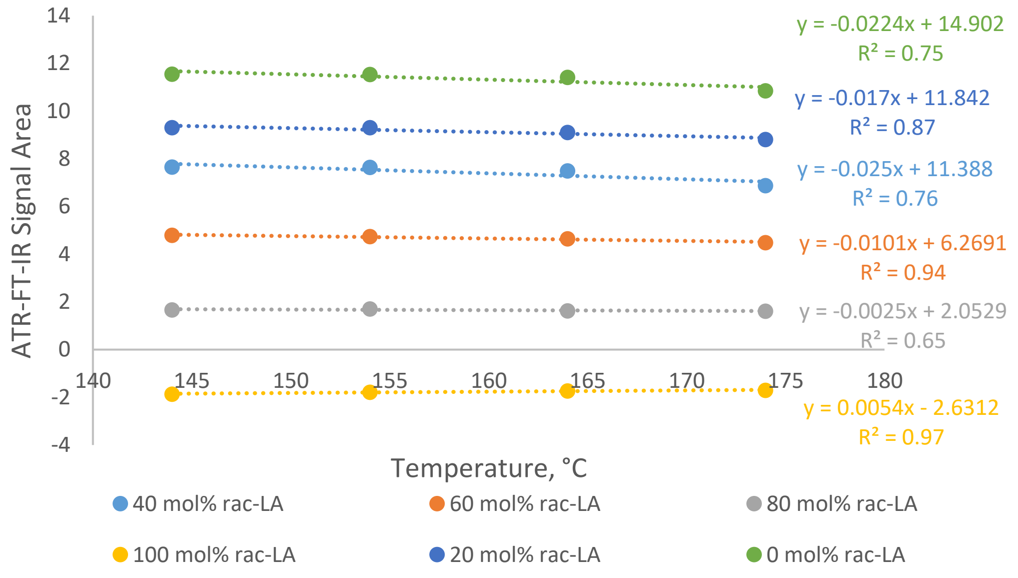

Figure S16. Variation of integrated PLA ATR-FT-IR signal area with temperature for *rac*-LA/ PLA mixtures of various composition

2 Kinetic studies of lactide polymerization

2.1 *In-situ* ATR-FT-IR-monitored polymerization of *rac*-LA or *L*-LA

2.1.1 Plots of lactide concentration *versus* time for various catalyst loadings

Table S3. Polymerization data for determination of order in catalyst, **1**, and propagation rate constant k_p for the ROP of *rac*-LA initiated by ***f1*** (reproduced from main paper)

Entry	Duration, mins	[a] [Zr], ppm	[Zr], mol%	[ROH], mol%	[b] Conversion, %	[c] M_n^{Theo}, g mol^{-1}	[d] M_n^{GPC}, g mol^{-1}	[d] $Đ_M$	[e] k_{obs}, min^{-1}
IR-14	183	40	6.50x10^{-3}	0.65	78	17500	7900	1.09	N/A
IR-15	160	61	9.90x10^{-3}	0.99	91	13500	15100	1.08	4.65x10^{-2}
IR-6	75	81	1.30x10^{-2}	1.30	91	10500	9250	1.08	6.72x10^{-2}
IR-16	105	110	1.77x10^{-2}	1.76	94	8000	7900	1.10	9.06x10^{-2}
IR-7	62	121	1.95x10^{-2}	1.95	93	7000	6700	1.07	9.86x10^{-2}
IR-17	60	158	2.53x10^{-2}	2.53	95	5500	12650	1.09	0.1229
[f]*IR-5*	315	0	0	0.25	4	N/A	N/A	N/A	2x10^{-4}

Conditions: 20 g of *rac*-LA, solvent-free, 174 °C, ***f1*** used. [a] Metal concentration, ppm by weight, calculated assuming $[Zr]_{f1}$ = 4.27 x 10^{-2} mol dm^{-3}. [b] Conversion determined *via* ^{1}H NMR spectroscopy, by integration of LA and PLA methine resonances. [c] M_n^{Theo} calculated from conversion and alcohol concentration, $\left\{\left(M_{r,LA} \times \frac{\%_{conv}}{100} \times \frac{[LA]}{[ROH.]}\right) + M_{r,BnOH} + M_{r,LA}\right\}$. [d] Determined *via* GPC analysis in THF using Triple Detection. [e] Rate constants determined via initial rate analysis. [f] Control reaction; no catalyst, 0.25 mol% BnOH.

For reactions IR-14, IR-15, IR-6, IR-16, IR-7, IR-17, (Table S3) the concentration of the monomer was plotted against time (Figure S17). The reaction time required to reach, for example, 50 % conversion, was much higher for reaction IR-14, than for any other reaction. It is therefore plausible that catalyst deactivation occurred to a much greater extent in that system, than in any of the other reactions, leading to deviation from a first order rate dependency with respect to the catalyst concentration.

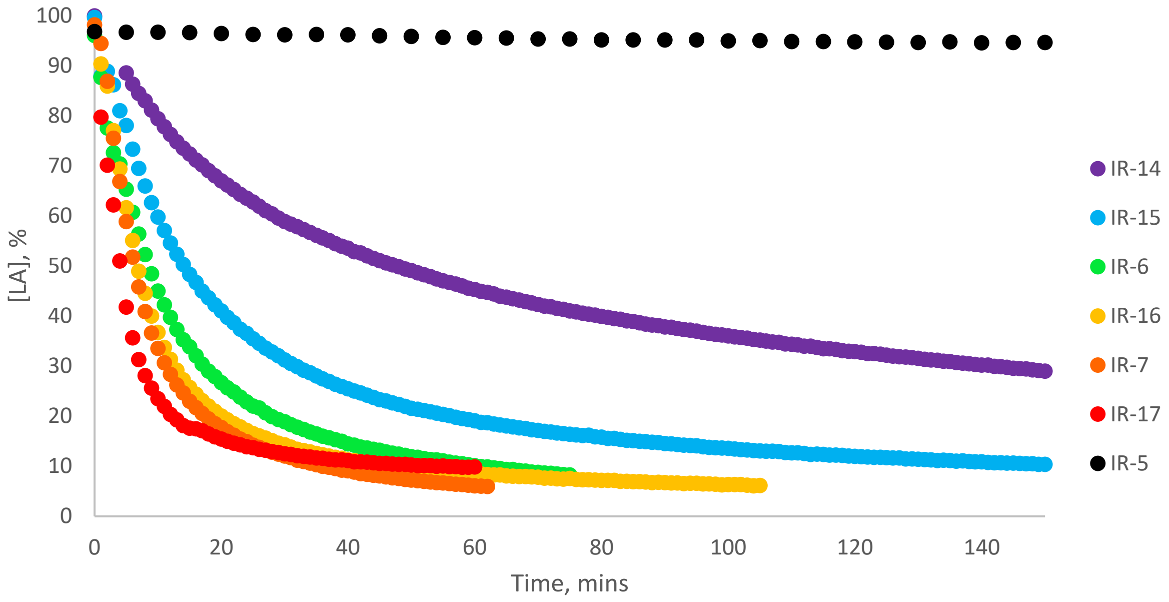

Figure S17. Plots of [*rac*-LA] versus time, for reactions IR-14, IR-15, IR-6, IR-16, IR-7, and IR-17, time-resolved data showing the variation in reaction times required to reach high conversion at different catalyst loadings. Labels refer to entry numbers in Table S3.

2.1.2 Plots of lactide concentration *versus* time for comparison of solid 1 and formulation f1

Comparison of the reaction kinetics afforded by solid catalyst **1** (with exogenous BnOH) and formulation ***f1***, respectively, were compared at two catalyst loadings. Whereas the reaction profiles corresponding to use of ***f1*** (IR-6, IR-7)described smooth curves, characteristic of *pseudo*-first order reaction kinetics, those produced using the solid catalyst were erratic and unpredictable. The latter reactions were, for this reason, repeated, with the unpredictable nature of the kinetics thus being confirmed.

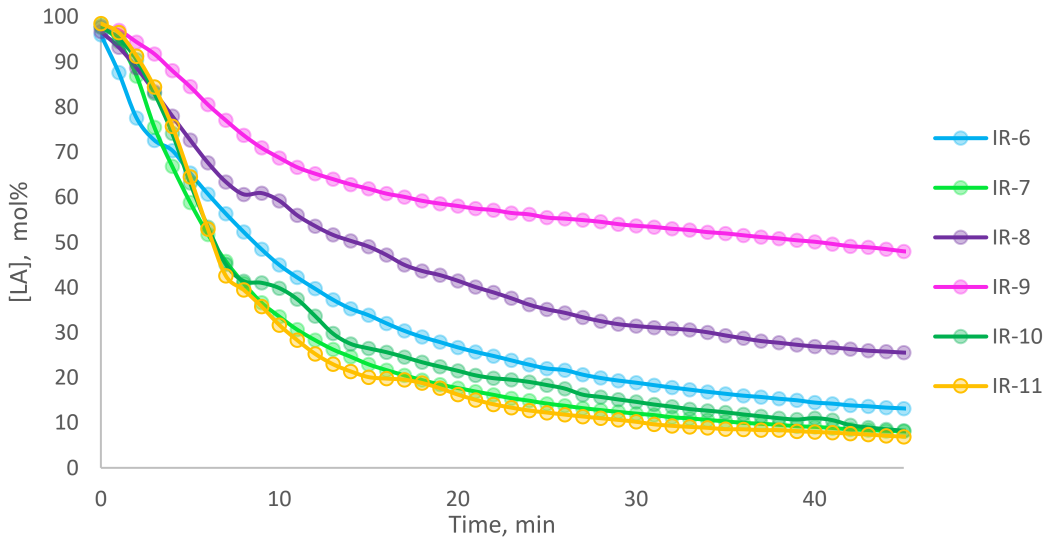

Figure S18. Plots of [*rac*-LA] versus time, for reactions IR-6, IR-7, IR-8, IR-9, IR-10, and IR-11, showing the erratic nature of the kinetics afforded by solid catalyst **1**, relative to the predictable reaction course produced by ***f1***. Labels refer to entry numbers in Table 3 of the main paper.

2.1.3 Variable Time Normalisation Analysis

Variable Time Normalisation Analysis (VTNA) was applied to the reaction monitoring data from reactions IR-14, IR-15, IR-6, IR-16, IR-7, IR-17.[5,6] The time axis on a plot of [LA] *versus* t was normalized by multiplication of values by [Zr] (Figure S18). In an ideal immortal ROP process, wherein the reaction rate exhibits a first order rate dependence on the catalyst concentration and a zero-order dependence on the co-initiator concentration, plots of [LA] against [Zr]t constructed using data from reactions with various catalyst loadings should be superimposed (irrespective of [ROH]). However, in this work, plots corresponding to catalyst loadings of $[Zr] < 1x10^{-3}$, deviated from the superimposition exhibited at higher [Zr], with the lowest [Zr] giving rise to the greatest deviation. This is attributed to catalyst deactivation occurring due to air ingress over the longer reaction times necessary for high conversion to be reached in the presence of very low [Zr]. Indeed, significant deviation is only discernible on the VTNA plot where conversion has reached approximately 20% in the case of IR-14, and 40% in the case of IR-15 ([LA] = 80% and [LA] = 60%, respectively), both corresponding to approximately a 10 minute reaction time, by which point all other reactions had reached high conversion (>60%), mitigating any significant catalyst deactivation effects in those cases. Moreover, the relatively low conversions at which deviation is observed, and the limitation of this phenomenon to IR-14 and IR-15, as well as the predictable nature of the initial rate analyses corresponding to IR-15, IR-6, IR-16, IR-7 and IR-17 (and the adherence of data from the stringently air-free *ex-situ* kinetic study to the proposed rate law) indicate that increased viscosity of the ROP mixture in IR-14 and IR-15 was not the likely cause of deviation from superimposition of VTNA plots.

2.1.4 Initial rate analysis

The propagation rate constant, k_p, for the solvent-free ROP of *rac*-LA at 174 ˚C in the presence of ***f1***, was obtained *via* initial rate analysis, informed by VTNA (above, Table S3, Figure S18) as described in the main paper. Semi-logarithmic plots were used to determine the observed rate constant k_p at each of several catalyst loadings (Figure S19), and these values plotted against the catalyst concentration (Figure 6 in main paper) to provide k_p.

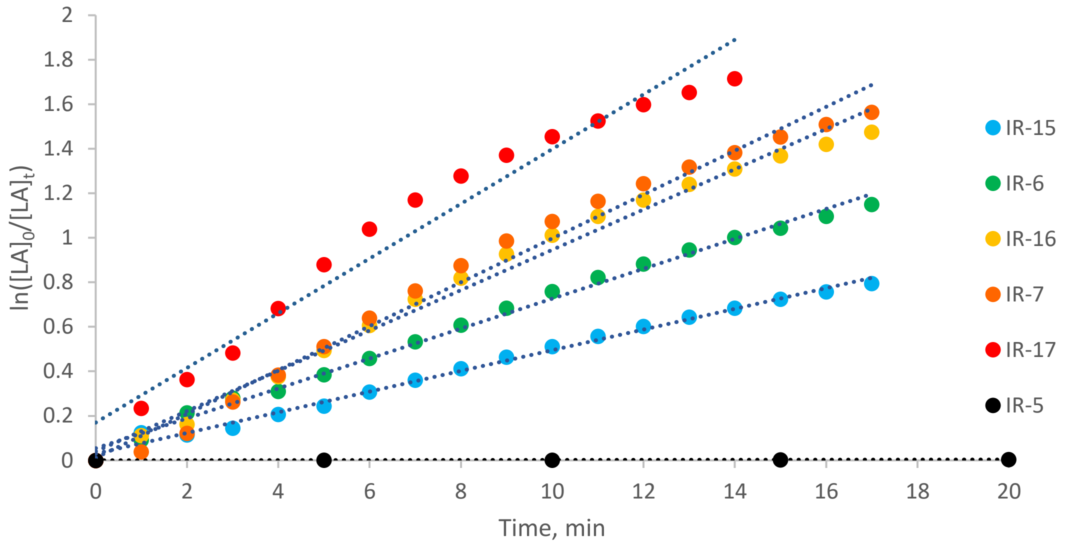

Figure S19. Semi-logarithmic initial-rate plots for the ROP of *rac*-LA in the presence of ***f1***. Labels refer to entry numbers in Table S3.

2.1.5 Addition of exogenous alcohol during polymerization

The effect of varying the molar [Zr]:[ROH] ratio on the rate of ROP was assessed by addition of exogenous BnOH to the ROP of *rac*-LA in the presence of ***f1***.

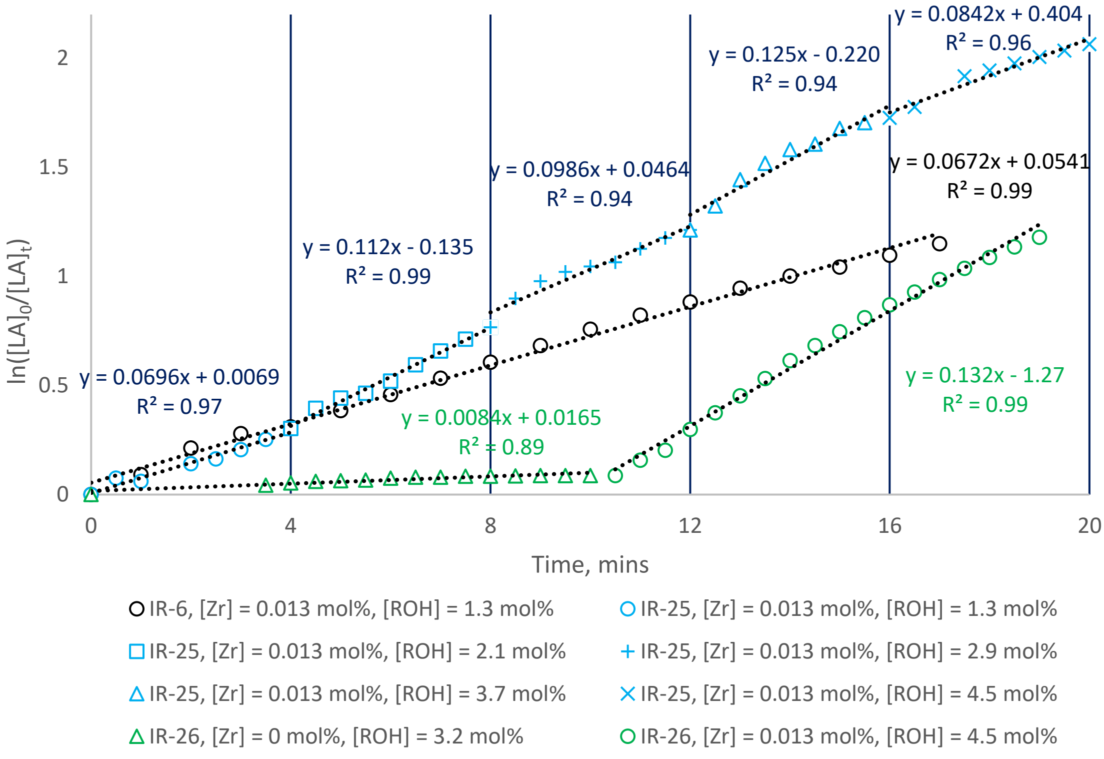

Figure S20. Semi-logarithmic plots corresponding to reactions carried out to investigate the effect of exogenous BnOH on the ROP of *rac*-LA in the presence of ***f1***. Vertical lines denote addition of BnOH. Labels refer to entries in Table S4.

Table S4. Polymerization data corresponding to reactions IR-6, IR-25, and IR-26, carried out to investigate the effect of exogenous BnOH on the ROP of *rac*-LA in the presence of ***f1***

Entry	Duration, mins		[c] [Zr], mol%	[ROH], mol%	[d] Conversion, %	[e] M_n^{Theo}, g mol^{-1}	[f] M_n^{GPC}, g mol^{-1}	[f] $Đ_M$	[g] k_{obs}, min^{-1}
IR-6	75		1.30x10^{-2}	1.30	92	10500	9250	1.08	6.72x10^{-2}
[a]*IR-25*	20	0-4	1.30x10^{-2}	1.30					6.9610^{-2}
		4-8	1.30x10^{-2}	2.10					0.112
		8-12	1.30x10^{-2}	2.90					9.86x10^{-2}
		12-16	1.30x10^{-2}	3.70					0.125
		16-20	1.30x10^{-2}	4.50	85	3000 / 5500	5650	1.18	8.42x10^{-2}
[b]*IR-26*	55	0-10	1.30x10^{-2}	3.20					8.4x10^{-3}
		10-55	1.30x10^{-2}	4.50	92	3000	3437	1.05	0.132

Conditions: 20 g of *rac*-LA, solvent-free, 174 °C, catalyst dosed as formulation ***f1***. [a] Aliquots of exogenous BnOH added after 4, 8, 12 and 16 minutes. [b] 3.2 mol% BnOH present from t = 0 min, then ***f1*** dosed after 10 min had elapsed ([Zr] = 1.30x10^{-2} mol%, [ROH] = 1.30 mol%). [c] ppm metal by weight. [d] Conversion determined *via* ^{1}H NMR spectroscopy, by integration of the monomer and polymer methine resonances. [e] M_n^{Theo} calculated from conversion and final alcohol concentration, for IR-6 $\left\{\left(M_{r,LA} \times \frac{\%_{conv}}{100} \times \frac{[LA]}{[ROH]}\right) + M_{r,BnOH} + M_{r,LA}\right\}$, for IR-25 and IR-26 $\left\{\left(M_{r,LA} \times \frac{\%_{conv}}{100} \times \frac{[LA]}{[ROH]}\right) + M_{r,BnOH} + \frac{1.3 \times M_{r,LA}}{4.5}\right\}$. First value of M_n^{Theo} for IR-25 assumes unlimited transesterification activity, yielding a single polymer distribution. Second value of M_n^{Theo} for IR-25 refers to heaviest distribution of expected pentamodal product, assuming negligible transesterification or chain scission activity. [f] Determined *via* GPC analysis in THF using Triple Detection. [g] Rate constants determined by initial rate analysis of *in-situ* ATR-FT-IR spectroscopic reaction monitoring data.

Four aliquots of 0.80 mol% BnOH (with respect to the monomer) were added at four-minute intervals to a polymerization initiated by ***f1*** ([Zr] = 1.3x10^{-2} mol%, [ROH] = 1.30 mol%; Table S4). The initial rate was in excellent agreement with that of IR-6, but a significant rate increase was observed after the first introduction of 0.80 mol% BnOH, with subsequent additions eliciting a much smaller effect, as would be expected at higher conversion. In a plot of [LA] *versus* time, a brief period of very fast conversion was visible immediately after each addition of BnOH, suggesting rapid consumption of the primary alcohol (Figure S21. Similarly, when the relative rate of LA consumption, $-d[LA]/dt$, was plotted against time, large maxima were present both after initial injection of ***f1***, and after each subsequent addition of BnOH (Figure S22). This is attributed both to the rapid initiation, relative to propagation, characteristic of an immortal regime, and to a proposed inverse relationship between chain length and propagation rate.

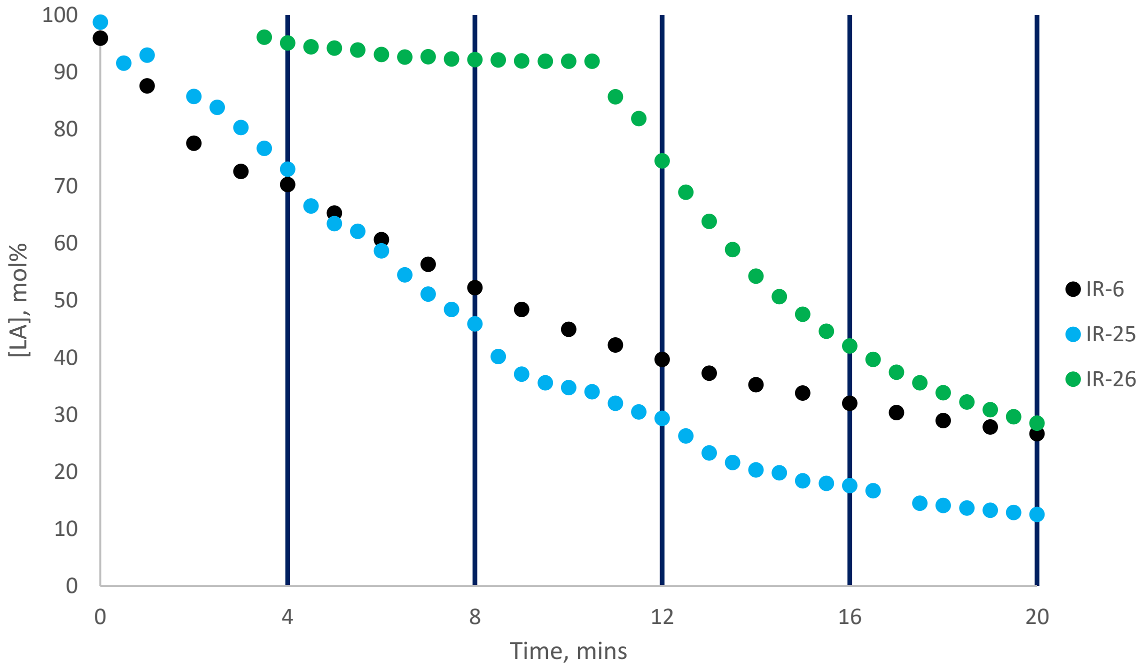

Figure S21. Plots of LA concentration against time for reactions IR-6, IR-25 and IR-26. Labels refer to entries in Table S4.

Each maximum in the plot of relative rate against time was immediately followed by a corresponding minimum, suggesting that injection of the exogenous alcohol at a location directly proximate to the ATR-FT-IR insertion probe, created a particularly large localised rate increase, which was followed by homogenisation of the mixture under mechanical stirring.

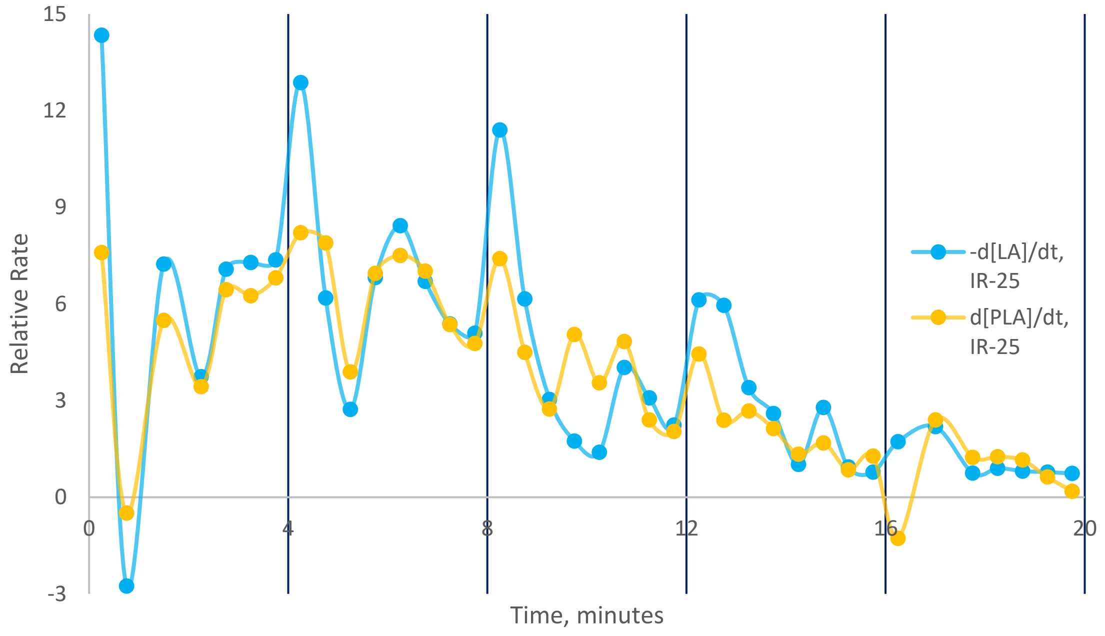

Figure S22. Plots of relative rate for reaction IR-25, the ROP of *rac*-LA in the presence of ***f1***, with stepwise addition of aliquots of exogenous BnOH at four-minute intervals (indicated by vertical lines). Two plots have been constructed, using calibrated data corresponding to the LA and PLA signals, respectively. Labels refer to entries in Table S4.

A plot of *d*[PLA]/*d*t also contained maxima corresponding to increased ring-opening activity following addition of the first three aliquots of BnOH. Consequently, a dilution effect due to BnOH injection in the vicinity of the ATR-FT-IR probe, followed by homogenisation, could be ruled out as the dominant effect. The [PLA]-derived maxima were smaller than those derived from [LA], likely due to the lactyl units adjacent to the benzyl or hydroxyl end groups, both generated in the initiation step, not appearing in the PLA region of the FT-IR spectrum, or to a minor dilution effect. The final addition of BnOH (at ~83% conversion) caused a minimum in the plot of *d*[PLA]/*d*t, commensurate with dilution becoming more significant than other factors at high conversion.

A further polymerization, IR-26, was carried out, in which ***f1*** ([Zr] = $1.3x10^{-2}$ mol%, [ROH] = 1.30 mol% ROH) and 3.2 mol% of exogenous BnOH were present from the beginning of the reaction (***f1*** added after 10 minutes of heating the monomer and BnOH at 174 ºC, during which negligible conversion occurred), the initial alcohol concentration therefore being equivalent to that in the final composition of IR-25 ([ROH] = 4.50 mol%, where [ROH] includes [BnOH]). In that case the rate was higher than when ***f1*** alone was used (IR-6), but did not significantly exceed that of IR-25 after addition of only 0.8 mol% of exogenous BnOH.

The increased rate was observed from the beginning of the reaction, and a first order rate dependence on the loading of ***f1*** used has been observed throughout the current work, allowing viscosity to be eliminated as the origin of the rate increase at higher alcohol concentrations. Similarly, the influence of differences in the respective steric and electronic properties of BnOH and polymer chains were ruled out on the basis that the rate discrepancy was clear throughout the reaction, from initiation to attainment of at least 70% conversion.

It is most plausible that the rate difference between IR-6 and the reactions to which exogenous BnOH was added (IR-25, IR-26) is due to the stoichiometry of co-initiator and catalyst species having a role in determining the rate below a [co-initiator]:[Zr] ratio of several hundred to one. This is somewhat reminiscent of phenomena observed by others for Sn(II)-based systems, and attributed to equilibrium formation of alkoxide species *in-situ*.[8,9] The magnitude of the rate increase observed on addition of 0.8 mol% of exogenous BnOH to IR-25, was approximately commensurate with a first-order rate dependence with respect to alcohol, whereas in the presence of 3.5 mol% of exogenous BnOH in IR-26, the rate relative to IR-6 indicated an order in alcohol of $<< 1$. This is consistent with there being a threshold ratio of [co-initiator]:[Zr] above which the reaction is zeroth-order in that species, with the data from IR-25 and IR-26 indicating that the ratio lies somewhere between [ROH]:[Zr] = 100:1 and [ROH]:[Zr] = 350:1, and likely toward the lower end of that range. The apparent relevance of this ratio in determining the rate of ROP is consistent with the calculated resting state of the catalyst being a binary hydrogen-bonded alcohol complex, and with the subsequent proposed LAAM mechanism. It is also compatible with the observed first order rate dependence on ***f1***, as irrespective of catalyst loading, use of this system always corresponds to a [ROH]:[Zr] molar ratio of 100:1.

The molecular weight of the main polymer product distribution of reaction IR-25, $M_n^{GPC} = 5650$ g mol^{-1}, was in agreement with the theoretical value for the heaviest product fraction, $M_n^{Theo} = 5500$ g mol^{-1}, calculated on the basis of five separate initiation events occurring, and the ROP adhering to ideal immortal ROP kinetics with negligible transesterification or chain scission activity. The percentage of the total monomer feed accounted for by each of the resulting polymer distributions was calculated from the percentage conversions reached at the time that each aliquot of BnOH was introduced (Figure S23). The lighter distributions were not detected *via* GPC indicating that when the PLA was purified by precipitation from methanol those product fractions remained in the solution phase, although a tail toward low molecular weight indicated that some low-weight chains were present.

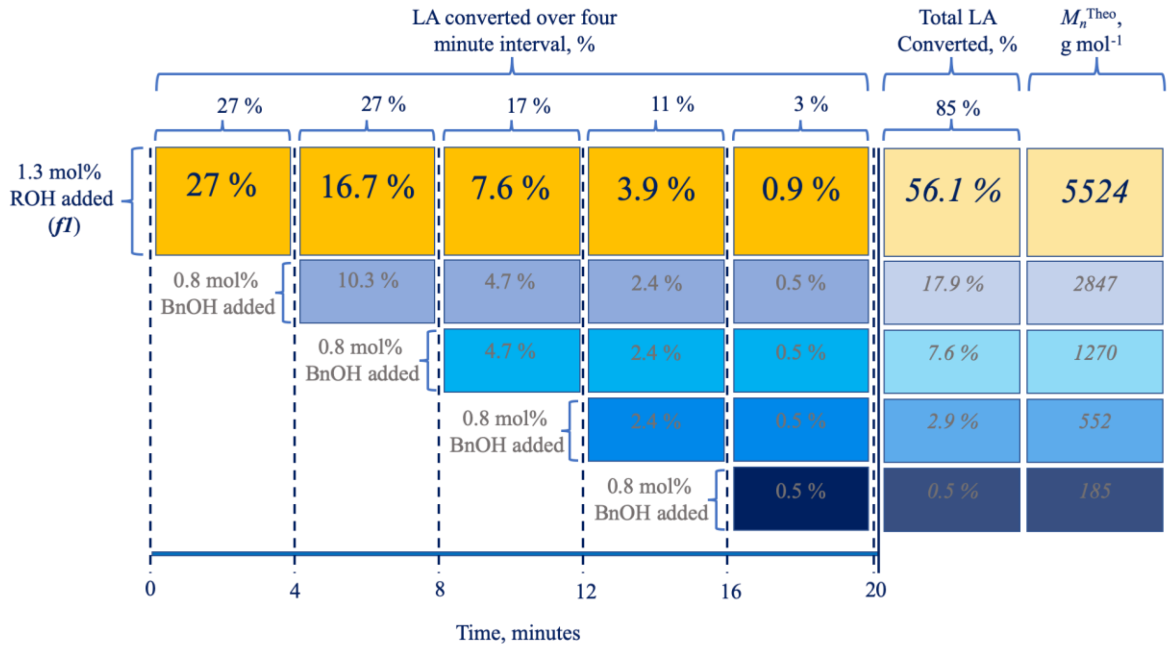

Figure S23. A visual representation of the chain growth anticipated to occur for the distribution of polymer chains corresponding to each initiation event in IR-25 (***f1***, followed by four aliquots of BnOH) in each four-minute interval, expressed as a percentage of the monomer feed, and the resulting theoretical molecular weight of each distribution, assuming all chains propagate at an equal rate at any given time

Although the exact nature of the relationship between alcohol concentration and ROP rate has not been established, the high, fixed ratio of [Zr]:[ROH] present in each of the formulations ***f1***, ***f2*** and ***f3***, respectively, already lends itself to the facile preparation of low-molecular weight PLA, should this be required. There is, accordingly, no foreseeable need to use an exogenous co-initiator alongside any of these formulations in an industrial setting. Moreover, a first order relationship between the quantity of a given formulation used and the rate has been clearly established, providing sufficient information for design of industrial processes. Further investigation of the role of the co-initiator was therefore deemed unnecessary in the current work.

2.2 Determination of k_{obs} *via ex-situ* kinetic analysis using parallel reactions

2.2.1 General procedure

In the glove box, 10 J Young's ampoules were each loaded with 1g *rac*-LA or *L*-LA, and 10 μL catalyst formulation ***f1*** ([Zr] = 3.1x10^{-3} mol%, [ROH] = 0.31 mol%). The ampoules were sealed and removed from the glovebox, and heated with vigorous stirring in an oil bath to 174 °C (control reaction B-1 was not heated). After the required reaction time, each ampoule was removed from the oil bath, cooled to ambient temperature and opened to air. The polymer was then dissolved in minimum (~5 ml) dichloromethane and a small aliquot of the resulting solution removed and evaporated to dryness for ^{1}H NMR analysis to determine conversion. The remaining polymer was isolated by precipitation from stirred methanol (~100 ml), which was then separated by decantation, and the polymer dried overnight under dynamic vacuum at 60 °C. Percentage conversion was plotted against

reaction time (each data point thus corresponding to a separate reaction) to produce a reaction profile (Figure S24). Polymer samples corresponding to reactions of conversion >70 % (reaction times ≥ 60 minutes; B-6, B-7, B-8, B-9, B-10) were purified by precipitation from methanol, and dried under dynamic vacuum at 60 °C for 16 hours, prior to analysis by GPC.

The observed rate constant, k_{obs} = 1.58x10^{-2} min^{-1}, 2.633x10^{-4} s^{-1}, determined *via ex-situ* analysis of reactions B-1 – B-10 was exactly in agreement with the value predicted using *in-situ* ATR-FT-IR reaction monitoring data (Table S5, Figure S24, Figure S25, and Figure 5 in main paper).

Table S5. Polymerization data for *ex-situ* kinetic study of ROP of lactide catalyzed by ***f1***

Entry	Duration, mins	[a] Conversion, %	[b] M_n^{Theo}, g mol^{-1}	[c] M_n^{GPC}, g mol^{-1}	[c] $Đ_M$	[d] TOF, h^{-1}
B-1	0	0	0	N/A	N/A	N/A
B-2	10	13	6500	N/A	N/A	25000
B-3	20	25	12000	N/A	N/A	24000
B-4	30	38	18000	N/A	N/A	25000
B-5	40	52	24500	N/A	N/A	25000
B-6	60	70	33000	26900	1.07	N/A
B-7	90	80	37500	43800	1.13	N/A
B-8	120	85	39500	40850	1.16	N/A
B-9	150	87	40500	47900	1.23	N/A
B-10	240	90	42000	93900	1.28	N/A

Conditions: 1 g of *rac*-LA, solvent-free, 174 °C, [Zr] = 3.1x10^{-3} mol%, [ROH] = 0.31 mol%; 19.3 ppm Zr by weight. Catalyst dosed as formulation ***f1***. [a] Conversion determined *via* ^{1}H NMR spectroscopy, by integration of the monomer and polymer methine resonances. [b] M_n^{Theo} calculated from conversion and alcohol concentration, $\left\{\left(M_{r,LA} \times \frac{\%_{conv}}{100} \times \frac{[LA]}{[ROH.]}\right) + M_{r,BnOH} + M_{r,LA}\right\}$. [c] Determined *via* GPC analysis in THF using Triple Detection. [d] TOF calculated from percentage conversion, determined via ^{1}H NMR spectroscopy, $\left\{TOF = \frac{[LA]_0 \times (1-[LA]_{t=n\,min}) \times \left(\frac{60}{n}\right)}{[Catalyst]}\right\}$

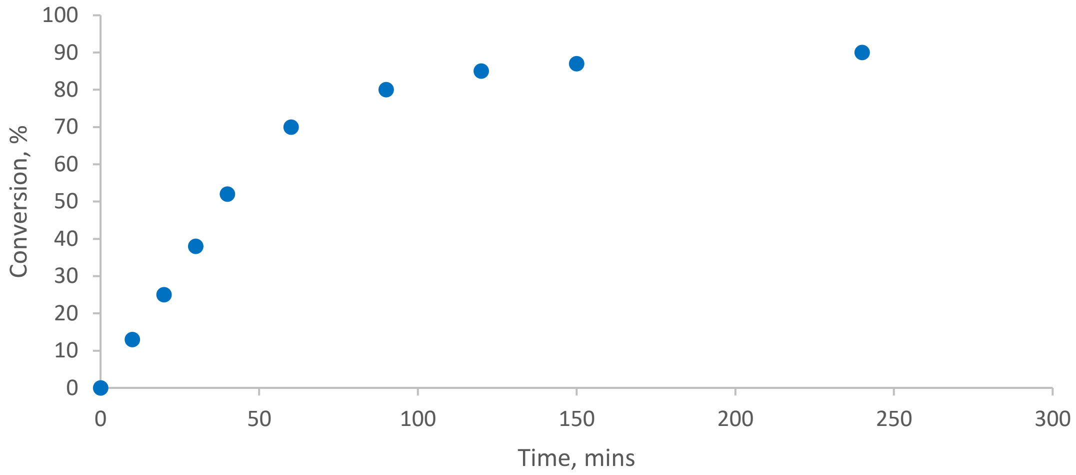

Figure S24. Reaction profile (conversion against time) for *ex-situ*-monitored 1 g-scale ROP of *rac*-LA catalyzed by ***f1*** (Table S5, Entries B-1 – B-10).

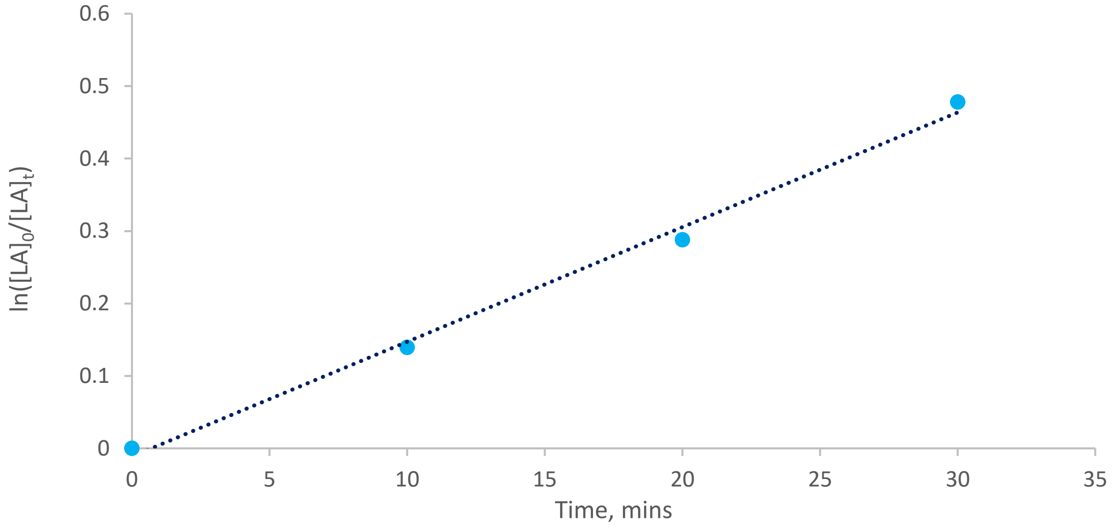

Figure S25. Semi-logarithmic initial rate plot for *ex-situ*-monitored 1 g-scale ROP of *rac*-LA catalyzed by ***f1*** (Table S5, Entries B-1 – B-4)

2.3 Kinetic isotope study

2.3.1 General procedure

Analysis of heteroselectivity suggests that, as expected, the active species is the same for the ROP of LA in the presence of **1** and ***f1***, respectively. Accordingly, the kinetic isotope study was undertaken using *deutero*- and *protio*-ethanol, which is readily obtained from commercial suppliers, whereas deuterated benzyl alcohol, BnOD is not. As preparation of a formulation such as ***f1*** is not possible with ethanol, the catalyst, **1**, and 100 equivalents (to ensure consistency with ***f1***) of the co-initiator, EtOH or EtOD, were added as a solution in toluene ([**1**] = 3.2 mmol dm^{-3}, [EtOH] = 320 mmol dm^{-3}). This method limited the catalyst loading that could be used, due to the low solubility of **1** in

toluene relative to in the oligomer-based solvent phase of ***f1***. However, it has been shown in the *ex-situ* kinetic study of the ROP of *rac*-LA catalyzed by ***f1***, that on a 1 g scale, in a sealed J Young's ampoule, kinetics representative of the rate law can be observed in the presence of $3.1x10^{-3}$ mol% **1**. Therefore, we used $3.1x10^{-3}$ mol% **1** for the kinetic isotope study, thus ensuring that the volume of toluene added to the polymerization (65 µl *per* 1 g of LA) was sufficiently small for the reaction to still be considered to occur under a solvent-free regime. The kinetic isotope study was carried out at a lower temperature, 150 °C, in order to reduce the relative reaction rates, and aid identification of any kinetic isotope effect. The more laborious parallel reactions method was used for this study, with all conversion values obtained via ^{1}H NMR spectroscopy, rather than *in-situ* ATR-FT-IR reaction monitoring. This was because the presence of toluene in the reaction mixture was anticipated to invalidate the calibration curves constructed for the ATR-FT-IR spectrometer. Furthermore, carrying out the reactions in sealed J Young's ampoules ensured none of the low-boiling ethanol co-initiator was lost *via* evaporation on exposure to the high-temperature reaction conditions, and that catalyst deactivation due to air ingress was prevented.

Process: At ambient temperature, in the glovebox, a solution was prepared of 30 mg **1** and 187 µl EtOH in 10 ml toluene. 65 µl of the resulting solution was added to each of seven J Young's ampoules, each containing 1 g *rac*-LA. The sealed ampoules were each then heated, with stirring, in an oil bath to 150 °C. The reactions (H-1 to H-7) were removed from the oil bath at time intervals of 10, 20, 30, 45, 60, 75, and 90 minutes, respectively (Table S6). Conversion in each case was determined *via* ^{1}H NMR spectroscopy. A second solution was prepared, containing 30 mg **1** and 187 µl deuterated ethanol EtOD in 10 ml toluene. A further seven polymerization reactions (D-1 to D-7) were carried out using the second solution (i.e. with deuterated alcohol present). For each series of seven parallel reactions, a semi-logarithmic initial rate plot was constructed for determination of k_{obs} (Figure S26). The crude products were analysed by GPC, with data appropriately adjusted to account for conversion.

The rate constant k_{obs} for the *protio* system at 150 °C ($1.66x10^{-2}$ min^{-1}) was very similar to observed that for the *ex-situ* kinetic study with $3.1x10^{-3}$ mol% **1** dosed as ***f1***, carried out at 174 °C ($1.58x10^{-2}$ min^{-1}). The occurrence of similar rates, despite a large temperature difference, can be attributed to catalyst deactivation due to the prolonged heating in the presence of a high alcohol concentration, required during preparation of ***f1*** (the use of a different alcohol is not significant after the non-rate-determining initiation step). However, as previously discussed, delivery of **1** as a saturated solution in toluene is not a viable method for producing PLA on an industrially relevant timescale without introducing unacceptably large volumes of toluene to the polymerization vessel.

Table S6. Polymerization data for kinetic isotope study of the ROP of *rac*-LA in the presence of solid **1** and exogenous ethanol

Entry	Duration, min	[c] Conversion, %	[d] M_n^{Theo}, g mol^{-1}	[e] M_n^{GPC}, g mol^{-1}	[e] $Đ_M$	[f] TOF, h^{-1}
[a] *H-1*	10	3.5	1673	-	-	7000
[a] *H-2*	20	13	6090	10150	1.00	13000
[a] *H-3*	30	20	9345	14250	1.04	13000
[a] *H-4*	45	40	18643	16450	1.05	17000
[a] *H-5*	60	52	24222	31900	1.02	17000
[a] *H-6*	75	64	29801	36050	1.04	17000
[a] *H-7*	90	79	36775	60150	1.10	17000
[b] *D-1*	10	2.7	1302	-	-	5000
[b] *D-2*	20	10	4696	8600	1.01	10000
[b] *D-3*	30	22	10275	14700	1.00	14000
[b] *D-4*	45	36	16784	21050	1.01	15000
[b] *D-5*	60	41	19109	26200	1.02	13000
[b] *D-6*	75	55	25618	39300	1.04	14000
[b] *D-7*	90	67	31197	43650	1.09	14000

Conditions: 1 g of *rac*-LA, solvent-free, 150 °C, [Zr] = 3.1x10^{-3} mol%, [ROH] = 0.31 mol%; 19.3 ppm Zr. Catalyst dosed as a 3.24x10^{-3} mol dm^{-3} solution in toluene. [a] *protio*-ethanol co-initiator. [b] *Deutero*-ethanol co-initiator. [c] Conversion determined *via* ^{1}H NMR spectroscopy, by integration of the monomer and polymer methine resonances. [d] M_n^{Theo} calculated from conversion and alcohol concentration, $\left\{\left(M_{r,LA} \times \frac{\%_{conv}}{100} \times \frac{[LA]}{[EtOH.]}\right) + M_{r,EtOH}\right\}$, wherein $M_{r,\mathrm{EtO1H}}$ = 46.069, and $M_{r,\mathrm{EtO2D}}$ = 47.000. Not approximated to nearest 500 g mol^{-1}, due to defined co-initiator (no formulation). [e] Determined *via* GPC analysis in THF using Triple Detection. [f] TOF calculated from percentage conversion, determined via ^{1}H NMR spectroscopy, $\left\{TOF = \frac{[LA]_0 \times (1-[LA]_{t=n\,min}) \times \left(\frac{60}{n}\right)}{[Catalyst]}\right\}$

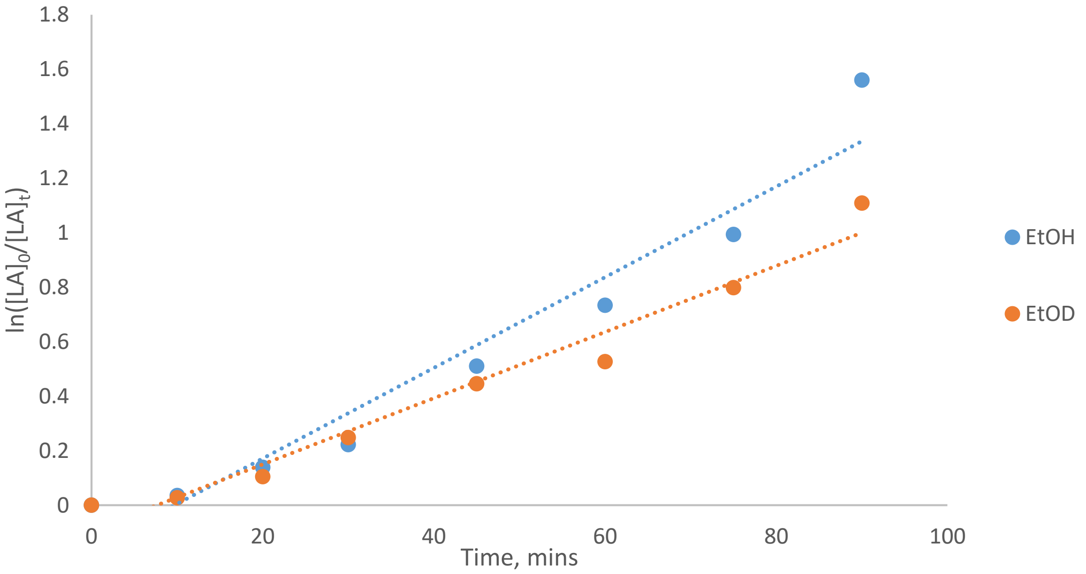

Figure S26. Semi-logarithmic plots for the ROP of *rac*-LA catalyzed by **1** in the presence of exogenous *deutero*- and *protio*-ethanol. Data for all constituent reactions is in Table S6.

2.4 Experimental determination of $\Delta G^{\ddagger}$ for the rate-determining step of propagation in the ROP of *rac*-LA initiated by *f1*

Determination of $\Delta G^{\ddagger}$ for the ROP of *rac*-LA at 174 ˚C was carried out *via* construction of an Eyring plot, using rate constants, k_{obs}, obtained through initial rate analysis of four solvent-free polymerizations, undertaken at temperatures between 144 ˚C and 174 ˚C (Table S7, Figure S27, Figure S28). The value obtained, $\Delta G^{\ddagger}$ = +32.5 kcal mol^{-1}, was satisfactorily consistent with the calculated value for the initiation step of the proposed mechanism ($\Delta G^{\ddagger}$ = +39.6 kcal mol^{-1}). The experimental value of $\Delta G^{\ddagger}$ confirms the validity of the other kinetic studies undertaken in the current work, whilst the computational studies have enabled elucidation of an accessible mechanistic pathway consistent with all experimental observations, and allowed the other apparent possibilities to be eliminated.

Due to the greater entropy associated with the presence of a growing polymer chain during propagation, than with the single BnOH molecule present during the modelled initiation event, it is assumed that the $\Delta G^{\ddagger}$ value calculated for initiation will exceed that of the more readily measured propagation event (which will exhibit some variation for each sequential chain growth event), in closer agreement with the experimental value. Due to the large size of the catalytic system it was not feasible to model propagation as well as initiation, especially given the expected mechanistic similarity of the two processes. Whilst the ROP of *L*-LA was considered *in-silico*, the experimental value was obtained using *rac*-LA, to ensure consistency with the wider kinetic study. Nonetheless, given the optical inactivity of **1**, and the stoichiometric nature of the modelled system (only a single monomer molecule was present in the system modelled), it is not anticipated that the calculated value of $\Delta G^{\ddagger}$ of initiation would differ significantly for *D*-LA. Moreover, in propagation, $\Delta G^{\ddagger}$ for heterotactic enchainment will be slightly lower in the presence of **1** than $\Delta G^{\ddagger}$ for isotactic enchainment (corresponding to heteroselectivity described by P_r = 0.67 for the ROP of *rac*-LA in the presence of **1**, and isotactic enchainment being necessary in the presence of an enantiopure *L*-LA feed). Accordingly, an experimentally obtained value of $\Delta G^{\ddagger}$ for propagation in the ROP of *L*-LA would be anticipated to exhibit closer agreement with the calculated value.

Table S7. Polymerization data for the ROP of *rac*-LA catalyzed by ***f1*** *at* several temperatures for the determination of ΔG‡ (reproduced from main paper)

Entry	Temperature, ° C	Duration, min	[a]Conversion, %	[b]M_n^{Theo}, g mol^{-1}	[c]M_n^{GPC}, g mol^{-1}	[c]$Đ_M$	[d]k_{obs}, min^{-1}
IR-6	174	75	91	10500	9250	1.08	6.72x10^{-2}
IR-18	166	60	88	10000	9050	1.03	6.02x10^{-2}
IR-19	159	120	88	10000	7500	1.04	4.17x10^{-2}
IR-20	144	240	84	9500	5100	1.02	1.89x10^{-2}

Conditions: 20 g *rac*-LA, solvent-free, ***f1*** used. [Zr] = 1.3x10^{-2} mol%, [ROH] = 1.3 mol%; 15.6 ppm Zr). [a] Conversion determined *via* ^{1}H NMR spectroscopy, by integration of the monomer and polymer methine resonances. [b] M_n^{Theo} calculated from conversion and alcohol concentration, $\left\{\left(M_{r,LA} \times \frac{\%_{conv}}{100} \times \frac{[LA]}{[ROH.]}\right) + M_{r,BnOH} + M_{r,LA}\right\}$. [c] Determined *via* GPC analysis in THF using Triple Detection. [d] Rate constants determined by initial rate analysis *via in-situ* ATR-FT-IR spectroscopic reaction monitoring.

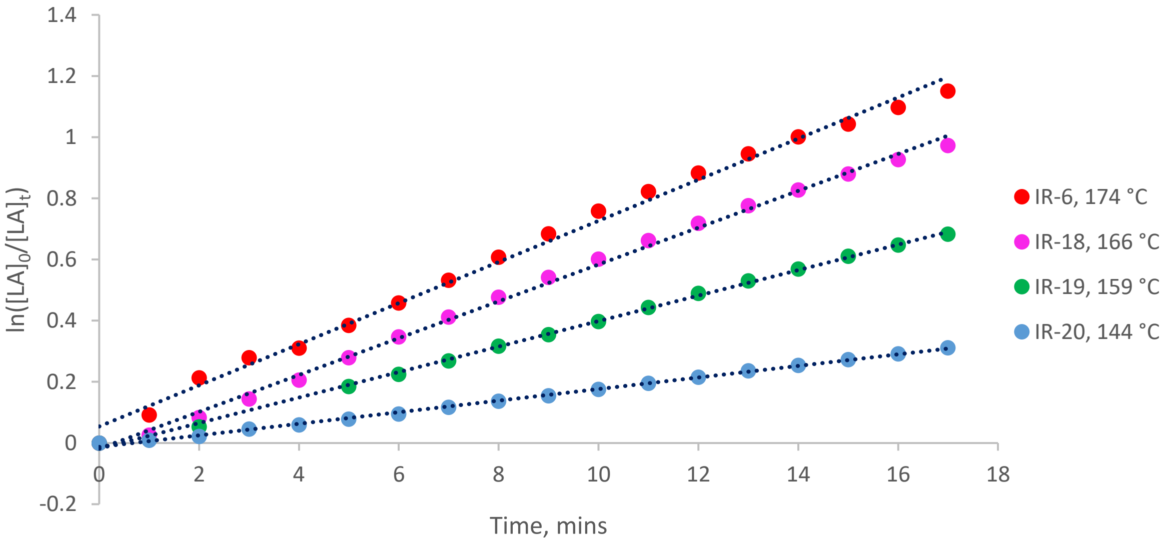

Figure S27. Initial rate plots for the ROP of rac-LA in the presence of f1 constructed using data gathered at temperatures between 144 ˚C and 174 ˚C. Labels refer to entry numbers in Table S7.

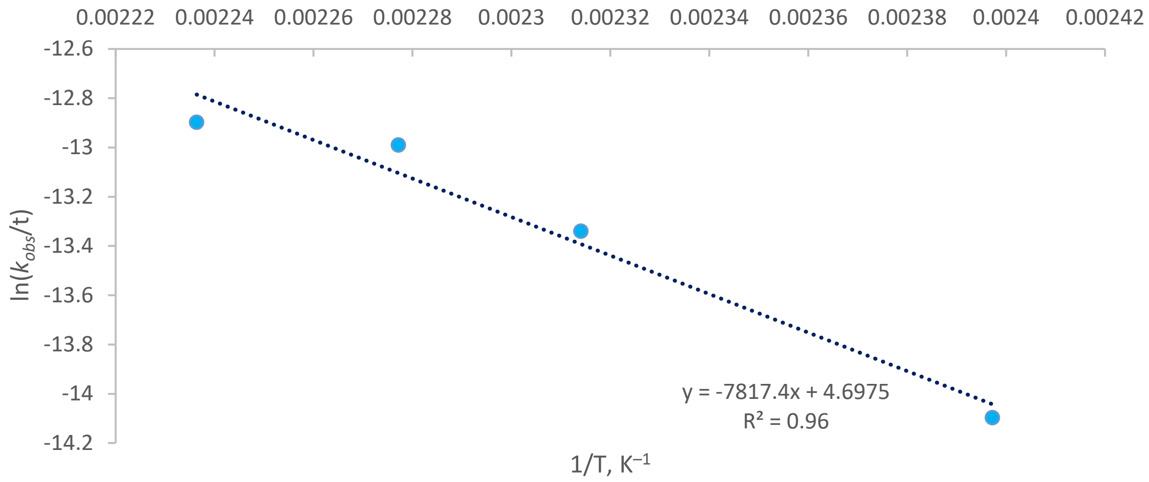

Figure S28. Eyring plot for the determination of $\Delta G^{\ddagger}$ for the ROP of *rac*-LA in the presence of ***f1***

3 Polymerization of *L*-LA in the presence of *f1*, *f2* and *f3* under industrially relevant conditions

3.1 General procedure

For a 500 gram-scale reaction, a jacketed stainless-steel reactor was loaded with 510 g of polymer-grade *L*-LA, and placed under a dry nitrogen atmosphere. The reactor was then heated to 130 °C using a Huber temperature control system, by passage of a thermal fluid through the reactor's outer jacket. An internal thermocouple was used to maintain the desired process temperature. When the contents of the reactor reached 130 °C, mechanical stirring was commenced, and a 10 g sample of the molten monomer drawn for free acid analysis. The free acid concentration of the monomer melt prior to introduction of the catalyst was typically around 3 – 4 meq (specific values not disclosed). The reactor was then opened, and the required catalyst formulation (unless stated otherwise) injected *via* micro syringe. The reactor was then resealed and heated to 180 °C. Aliquots of the polymerization mixture were withdrawn at various time intervals, and immediately cooled on ice. Conversion was determined *via* ^{1}H NMR spectroscopy (by integration of the monomer and polymer methine signals), and molecular weight data was obtained *via* GPC. Due to the insolubility of the high-molecular weight P*L*LA in THF, crude samples were typically diluted to 0.2 mg ml^{-1}, and molecular weight data was determined using a refractive index detector, calibrated against polystyrene standards, and with application of a Mark-Houwink factor of 0.58. The bulk PLA from polymerization reaction IE-3 was successfully processed and formed into crystalline pellets.

All polymerizations using ***f1***, ***f2***, or ***f3*** under industrially relevant conditions yielded well-controlled immortal polymerization kinetics, with excellent correlation between conversion and molecular weight, and effective suppression of side reactions (Table S8, Figure S29, Tables S9 – S13, Figures S30 – S39). $Sn(Oct)_2$ afforded a high reaction rate, but on reaching equilibrium (>90% conversion) the polymer molecular weight, M_n^{GPC}, began to decrease, and $Đ_M$ increased dramatically, indicating extensive degradation, presumably resulting from chain scission and transesterification

processes (Table S14, Figures S40, S41). In consistency with other formulation-initiated polymerizations reported in the current work, and reflecting the many variables associated with catalyst formulation prior to use, including co-initiation by an ill-defined oligomeric mixture, use of several different catalyst formulations, and of an unpurified LA feed, theoretical molecular weights, M_n^{Theo} have been reported to the nearest 500 g mol^{-1} only.

In none of the experiments described was any indication of depolymerization activity by **1** observed under the ROP conditions. PLA from reaction IE-3 underwent several processing steps, including multiple thermal processes, to yield crystalline resin pellets. The resulting pellets were then stored under air for 12 months, after which GPC analysis revealed only a modest reduction in M_n relative to that of the crude product, analysed prior to processing, from 37 700 g mol^{-1} to 29 500 g mol^{-1}. $Đ_M$ increased slightly, from 1.30 to 1.43. Such stability toward depolymerization and chain scission (hydrolysis) during processing and storage, regardless of the continued presence of Zr, appears sufficient for many short- and medium-term applications, without the need for stabilization.

Table S8. Polymerization data for the ROP of *L*-LA in the presence of catalyst formulations ***f1***, ***f2***, and ***f3***, under industrial conditions (reproduced from main paper)

Entry	Formulation	Duration, mins	[e] [Metal], ppm	[Metal], mol%	[ROH], mol%	[f] Conversion, %	[g] M_n^{Theo}, g mol^{-1}	[h] M_n^{GPC}, g mol^{-1}	[h] $Đ_M$	[i] k_{obs}, min^{-1}
[a,j] *IE-1*	***f1***	210	18 + 9	2.9×10^{-3} + 1.5×10^{-3}	0.29 + 0.15	71	N/A	[k] 26900, 9700	[k] 1.17	4.2×10^{-3}
[b] *IE-2*	***f3***	120	50	8.1×10^{-3}	1.62	94	8500	8750	1.18	3.16×10^{-2}
[b] *IE-3*	***f3***	1350	9	1.5×10^{-3}	0.29	91	45500	37700	1.30	3.4×10^{-3}
[b] *IE-4*	***f2***	240	12	1.9×10^{-3}	0.29	78	39000	32200	1.27	7.8×10^{-3}
[c] *IE-5*	***f2***	390	8	1.3×10^{-3}	0.19	70	53500	41350	1.36	3.5×10^{-3}
[d] *IE-6*	[l] ***Sn(Oct)₂ + Co-In.***	120	44	5.3×10^{-3}	[i] 0.29	93	46000	41800	1.94	4.01×10^{-2}

Conditions: *L*-LA, solvent-free, 180 °C. [a] 2000 g of monomer. [b] 500 g of monomer. [c] 600 g of monomer. [d] 750 g of monomer. [e] Metal concentration, ppm by weight, calculated assuming $[Zr]_{f1} = 4.270 \times 10^{-2}$ mol dm^{-3}, $[Zr]_{f2} = 2.850 \times 10^{-2}$ mol dm^{-3}, $[Zr]_{f3} = 2.135 \times 10^{-2}$ mol dm^{-3}. [f] Conversion determined *via* ^{1}H NMR spectroscopy, by integration of the monomer and polymer methine resonances. [g] M_n^{Theo} calculated from conversion and alcohol concentration, $\left\{\left(M_{r,LA} \times \frac{\%_{conv}}{100} \times \frac{[LA]}{[ROH]}\right) + M_{r,BnOH} + M_{r,LA}\right\}$, except in the case of reaction IE-6, where the M_r of the co-initiator has not been considered. Approximated to the nearest 500 g mol^{-1}, reflecting a reasonable degree of precision where ***f1*** and un-purified (technical) LA have been used. [h] Determined *via* GPC analysis in THF using a refractive index detector, calibrated against polystyrene standards. A Mark-Houwink factor of 0.58 has been applied. [i] Rate constants determined by initial rate analysis, using conversion values determined by ^{1}H NMR analysis of aliquots taken from the reactor during polymerization. [j] Additional catalyst formulation ***f1*** was added after 2 hours. [k] Molecular weight distribution bimodal. Only highest molecular weight peak used to determine $Đ_M$. [l] Initiator, "*Co-In*", not disclosed due to commercial sensitivity.

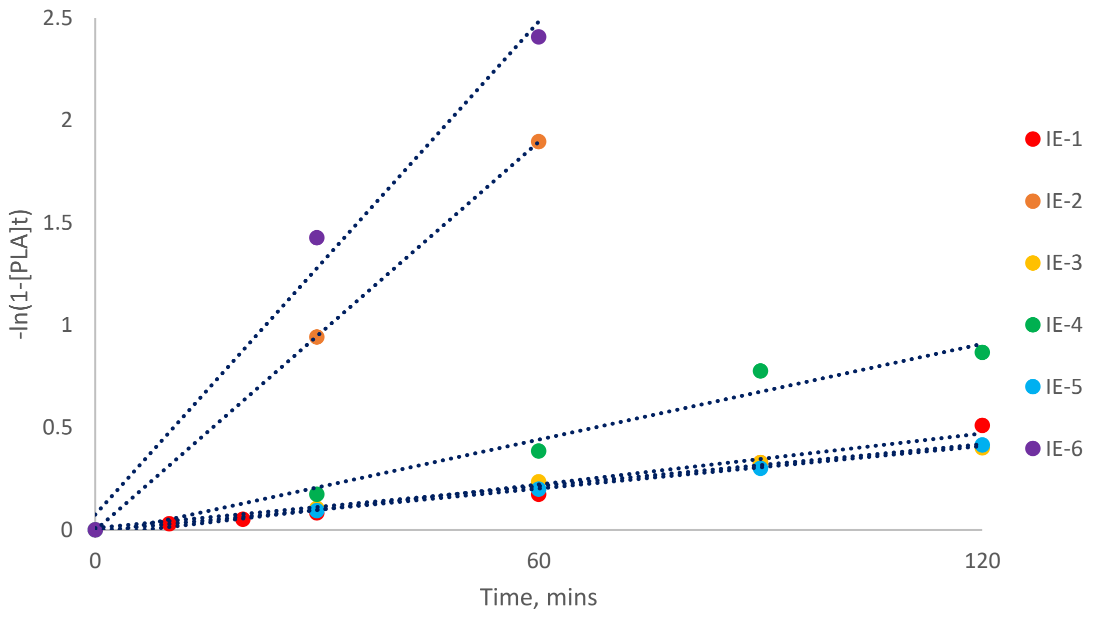

Figure S29. Semi-logarithmic plots for the ROP of *L*-LA catalyzed by formulations ***f1***, ***f2***, ***f3***, and $Sn(Oct)_2$ under industrially relevant conditions. Labels refer to entry numbers in Table S8.

Additional formulation ***f1*** was dosed into polymerization IE-1 after 2 hours reaction time had elapsed, and the polymerization continued for 1.5 hours thereafter. Accordingly, the GPC data obtained from the 120 minute sample exhibits a bimodal polymer molecular weight distribution, not seen in that of the 90 minute sample. Indicative of extremely effective suppression of transesterification activity, the M_n^{GPC} and $Đ_M$ values corresponding to the secondary peak are 9300 g mol^{-1} and 1.02, respectively.

Time-resolved M_n^{GPC} and $Đ_M$ data for all large-scale polymerizations (IE-1 – IE-6) are presented on the following pages.

Table S9. Conversion and molecular weight data for samples taken during polymerization reaction IE-1

Time, mins	[a] *Conversion, %*	[b] M_n^{GPC}, *g mol*$^{-1}$	[b] $Đ_M$
0	0	0	0
10	3	600	1.03
20	5	1000	1.13
30	8	1700	1.18
60	16	5350	1.13
90	28	10100	1.06
120	40	14750	1.04
	*Additional **f1** added after 120 minutes*		
210	71	26900/9700	1.15/1.03

Conditions: *L*-LA, solvent-free, 180 °C, 2000 g of monomer. 2.9×10^{-3} mol% Zr, and 0.29 mol% ROH introduced as ***f1***. A further 1.5×10^{-3} mol% Zr and 0.15 mol% ROH were added as ***f1*** after 120 minutes. [a] Conversion determined *via* ^{1}H NMR spectroscopy, by integration of the monomer and polymer methine resonances. [b] Determined *via* GPC analysis in THF using a refractive index detector, calibrated against polystyrene standards. A Mark-Houwink factor of 0.58 has been applied. A bimodal distribution was observed in the GPC, and only the higher-weight peak was analysed.

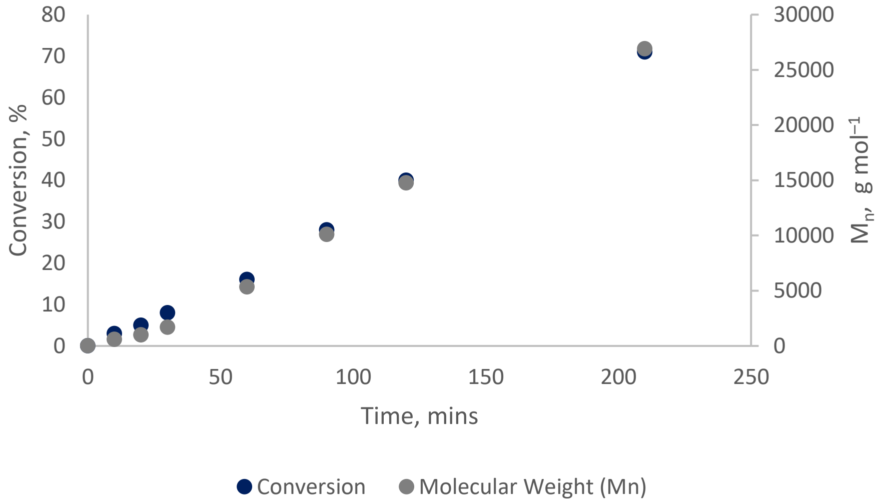

Figure S30. Plots of conversion and number average molecular weight, M_n^{GPC}, against time for reaction IE-1

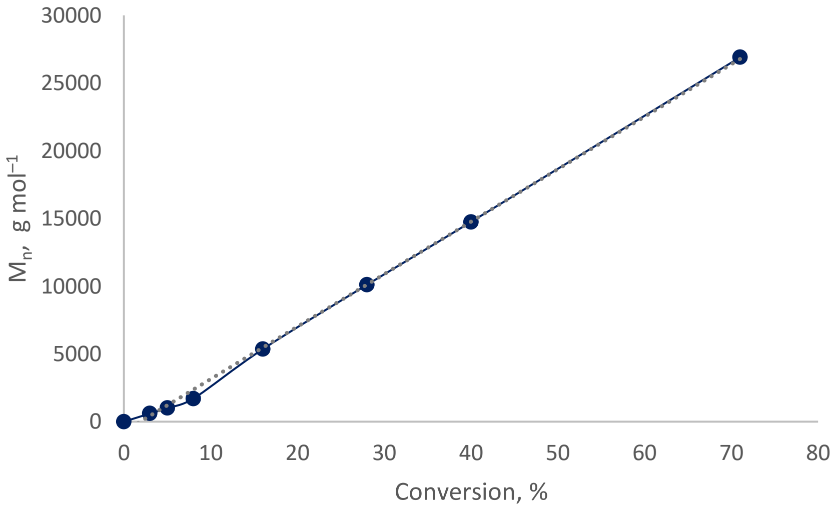

Figure S31. A plot of number average molecular weight, M_n^{GPC}, against conversion for reaction IE-1

Table S10. Conversion and molecular weight data for samples taken during polymerization reaction IE-2

Time, mins	[a] *Conversion, %*	[b] M_n^{GPC}*, g mol*$^{-1}$	[b] $Đ_M$
0	0	0	0
30	61	4850	1.07
60	85	7350	1.09
90	92	8650	1.11
120	94	8750	1.18

Conditions: *L*-LA, solvent-free, 180 °C, 500 g of monomer. 8.1x10^{-3} mol% Zr, and 1.62 mol% ROH introduced as catalyst formulation ***f3***. [a] Conversion determined *via* ^{1}H NMR spectroscopy, by integration of the monomer and polymer methine resonances. [b] Determined *via* GPC analysis in THF using a refractive index detector, calibrated against polystyrene standards. A Mark-Houwink factor of 0.58 has been applied.

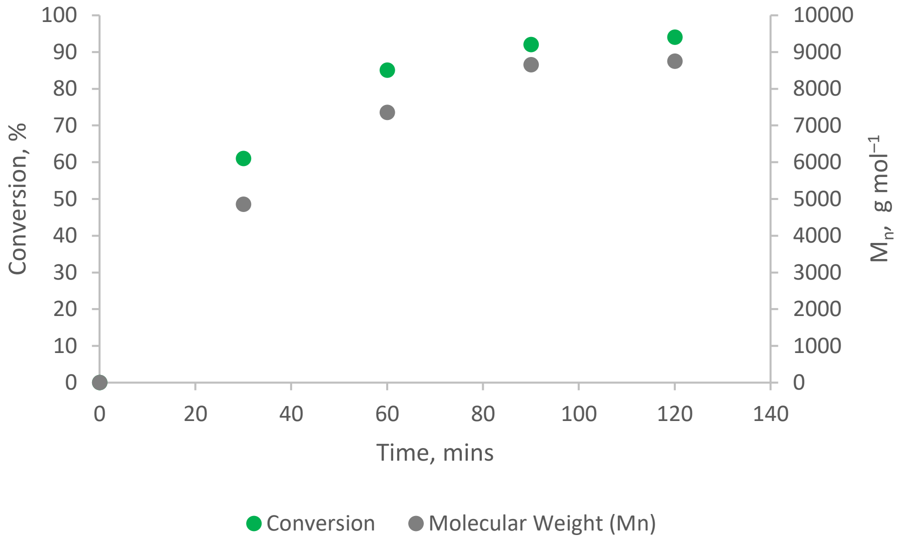

Figure S32. Plots of conversion and number average molecular weight, M_n^{GPC}, against time for reaction IE-2

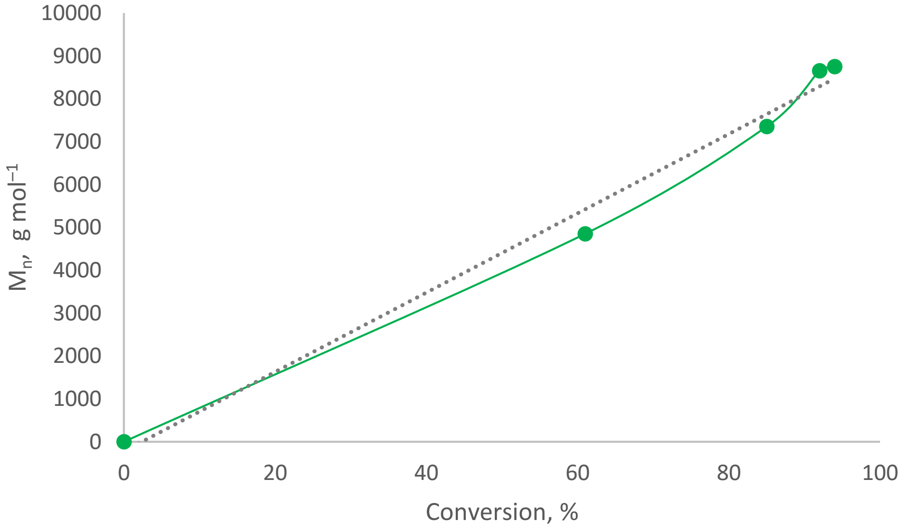

Figure S33. A plot of number average molecular weight, M_n^{GPC}, against conversion for reaction IE-2

Table S11. Conversion and molecular weight data for samples taken during polymerization reaction IE-3

Time, mins	[a] *Conversion, %*	[b] M_n^{GPC}*, g mol*$^{-1}$	[b] $Đ_M$
0	0	0	0
30	10	2800	1.17
60	21	6850	1.06
90	28	9950	1.07
120	33	12600	1.08
180	45	16750	1.13
240	51	20000	1.16
300	60	24300	1.12
420	71	27100	1.18
1350	91	37700	1.3

Conditions: *L*-LA, solvent-free, 180 °C, 500 g of monomer. 1.5x10^{-3} mol% Zr, and 0.29 mol% ROH introduced as catalyst formulation ***f3***. [a] Conversion determined *via* ^{1}H NMR spectroscopy, by integration of the monomer and polymer methine resonances. [b] Determined *via* GPC analysis in THF using a refractive index detector, calibrated against polystyrene standards. A Mark-Houwink factor of 0.58 has been applied.

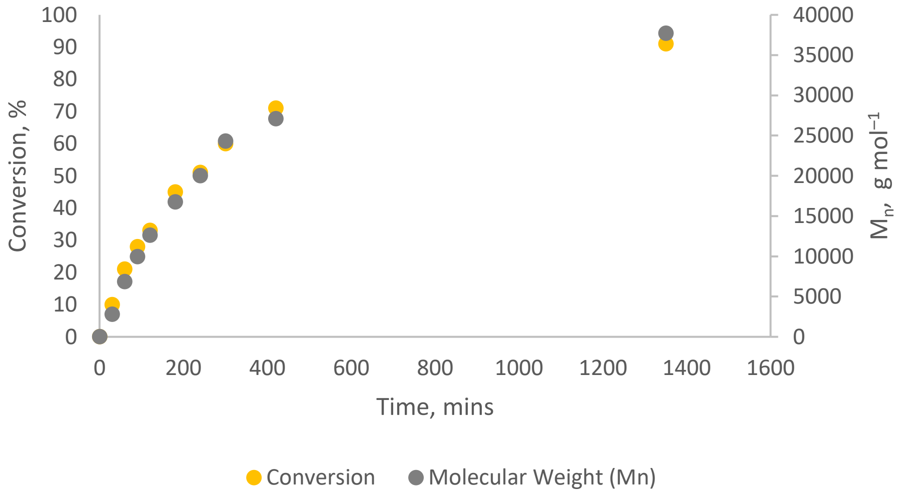

Figure S34. Plots of conversion and number average molecular weight, M_n^{GPC}, against time for reaction IE-3

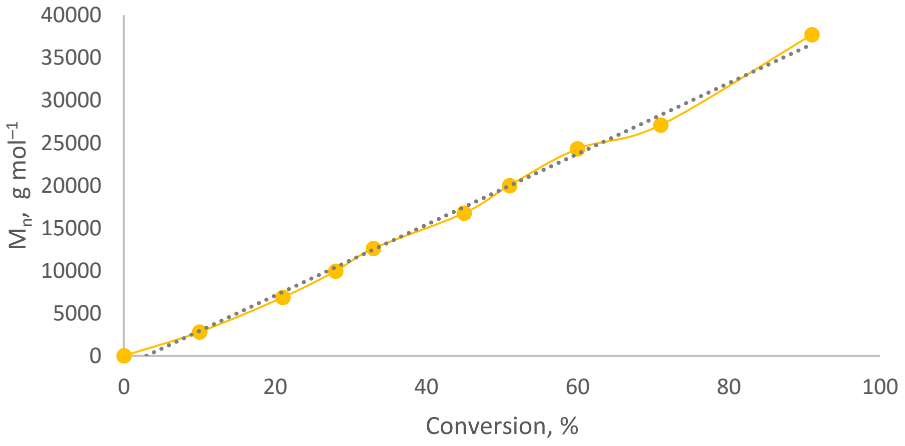

Figure S35. A plot of number average molecular weight, M_n^{GPC}, against conversion for reaction IE-3

Table S12. Conversion and molecular weight data for samples taken during polymerization reaction IE-4

Time, mins	[a] *Conversion, %*	[b] M_n^{GPC}, *g mol*$^{-1}$	[b] $Đ_M$
0	0	0	0
30	16	4600	1.18
60	32	11650	1.05
90	54	18700	1.13
120	58	25300	1.21
180	74	31250	1.22
240	78	32200	1.27

Conditions: *L*-LA, solvent-free, 180 °C, 500 g of monomer. 1.9x10^{-3} mol% Zr, and 0.29 mol% ROH introduced as catalyst formulation ***f2***. [a] Conversion determined *via* ^{1}H NMR spectroscopy, by integration of the monomer and polymer methine resonances. [b] Determined *via* GPC analysis in THF using a refractive index detector, calibrated against polystyrene standards. A Mark-Houwink factor of 0.58 has been applied.

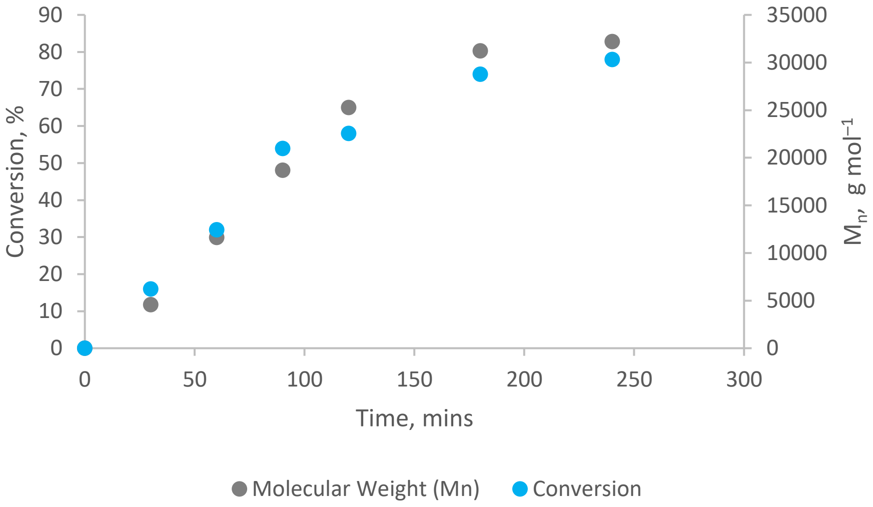

Figure S36. Plots of conversion and number average molecular weight, M_n^{GPC}, against time for reaction IE-4

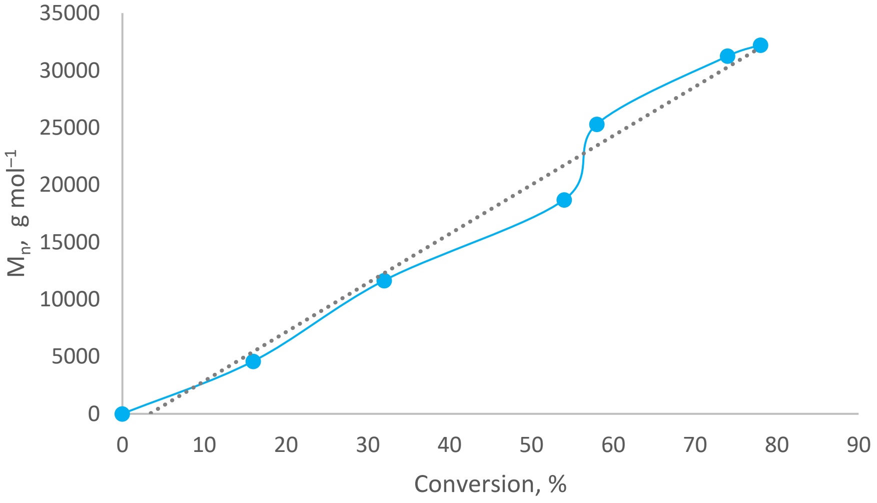

Figure S37. A plot of number average molecular weight, M_n^{GPC}, against conversion for reaction IE-4

Table S13. Conversion and molecular weight data for samples taken during polymerization reaction IE-5

Time, mins	[a] *Conversion, %*	[b] M_n^{GPC}, *g mol*$^{-1}$	[b] $Đ_M$
0	0	0	0
30	9	3300	1.24
60	18	9100	1.13
90	26	14550	1.09
120	34	20900	1.14
180	48	24150	1.33
240	57	30150	1.26
300	62	35150	1.25
360	68	42100	1.31
390	70	41350	1.36

Conditions: *L*-LA, solvent-free, 180 °C, 600 g of monomer. 1.3x10^{-3} mol% Zr, and 0.29 mol% ROH introduced as catalyst formulation ***f2***. [a] Conversion determined *via* ^{1}H NMR spectroscopy, by integration of the monomer and polymer methine resonances. [b] Determined *via* GPC analysis in THF using a refractive index detector, calibrated against polystyrene standards. A Mark-Houwink factor of 0.58 has been applied.

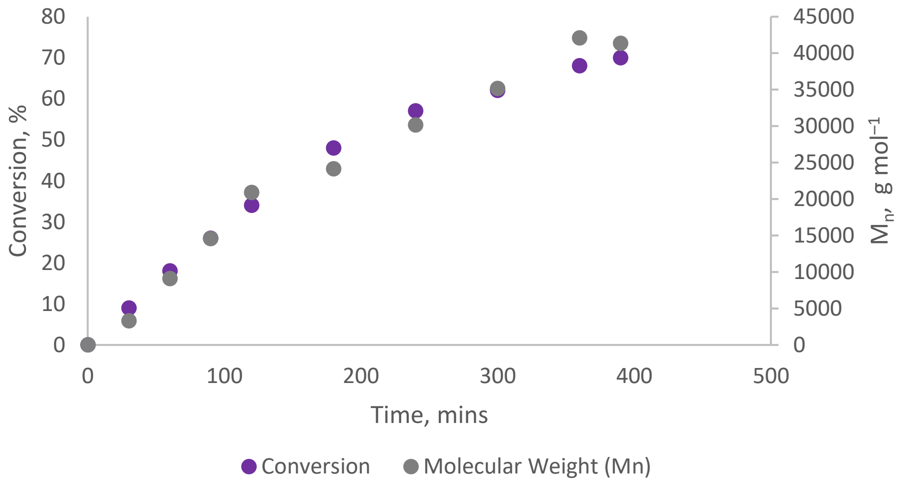

Figure S38. Plots of conversion and number average molecular weight, M_n^{GPC}, against time for reaction IE-5

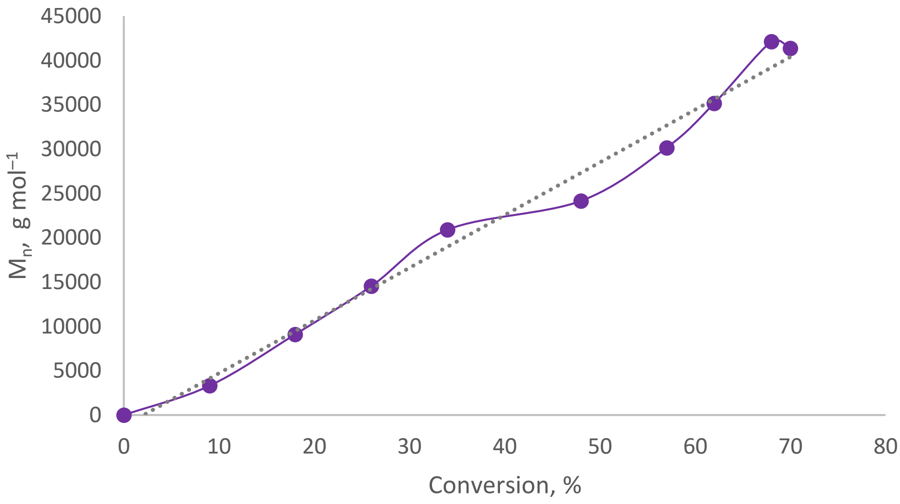

Figure S39. A plot of number average molecular weight, M_n^{GPC}, against conversion for reaction IE-5

Table S14. Conversion and molecular weight data for samples taken during polymerization reaction IE-6

Time, mins	[a] *Conversion, %*	[b] M_n^{GPC}, *g mol*$^{-1}$	[b] $Đ_M$
0	0	0	0
30	76	40350	1.12
60	91	50800	1.41
90	94	47400	1.62
120	93	41800	1.94

Conditions: *L*-LA, solvent-free, 180 °C, 750 g of monomer. 5.3x10^{-3} mol% $Sn(Oct)_2$, and 0.29 mol% initiator. [a] Conversion determined *via* ^{1}H NMR spectroscopy, by integration of the monomer and polymer methine resonances. [b] Determined *via* GPC analysis in THF using a refractive index detector, calibrated against polystyrene standards. A Mark-Houwink factor of 0.58 has been applied.

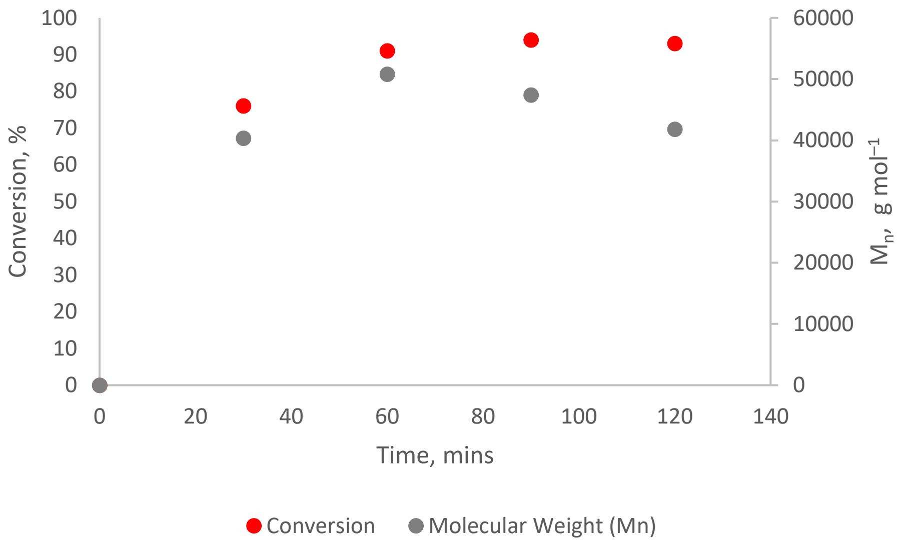

Figure S40. Plots of conversion and number average molecular weight, M_n^{GPC}, against time for reaction IE-6

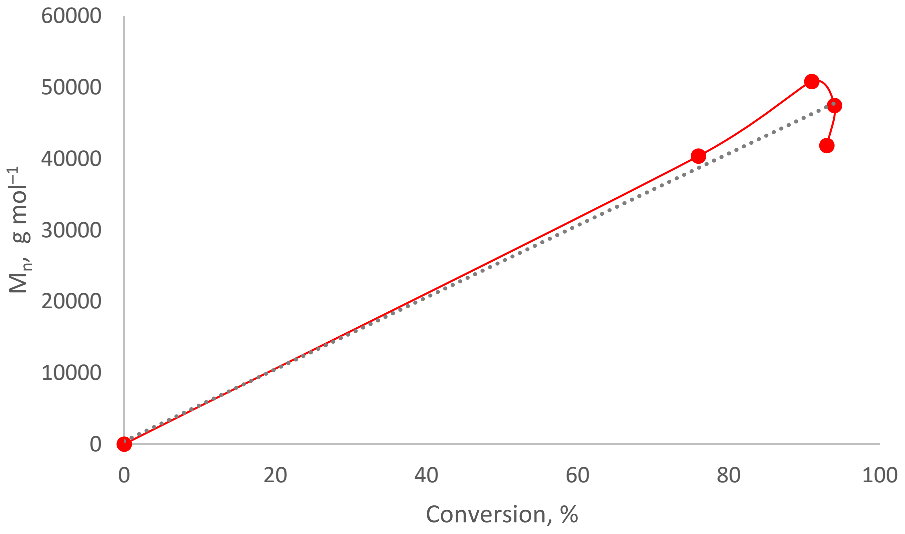

Figure S41. A plot of number average molecular weight, M_n^{GPC}, against conversion for reaction IE-6. The curve links datapoints chronologically, see Table S14.

4 Selected Gel Permeation Chromatograms

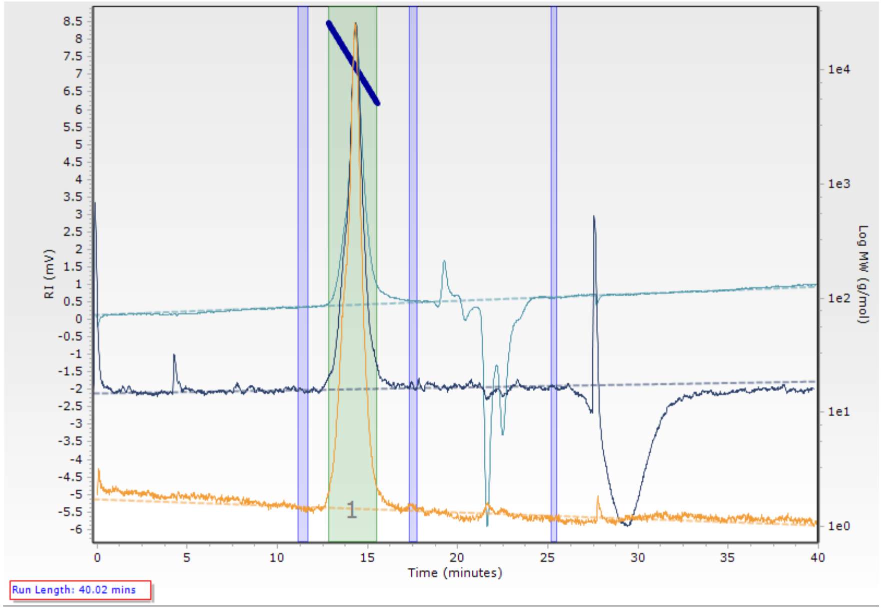

Figure S42. GPC trace for the polymer product of reaction IR-6; [Zr] = 1.30x10^{-2} mol%, [ROH] = 1.30 mol%, dosed as formulation ***f1***.

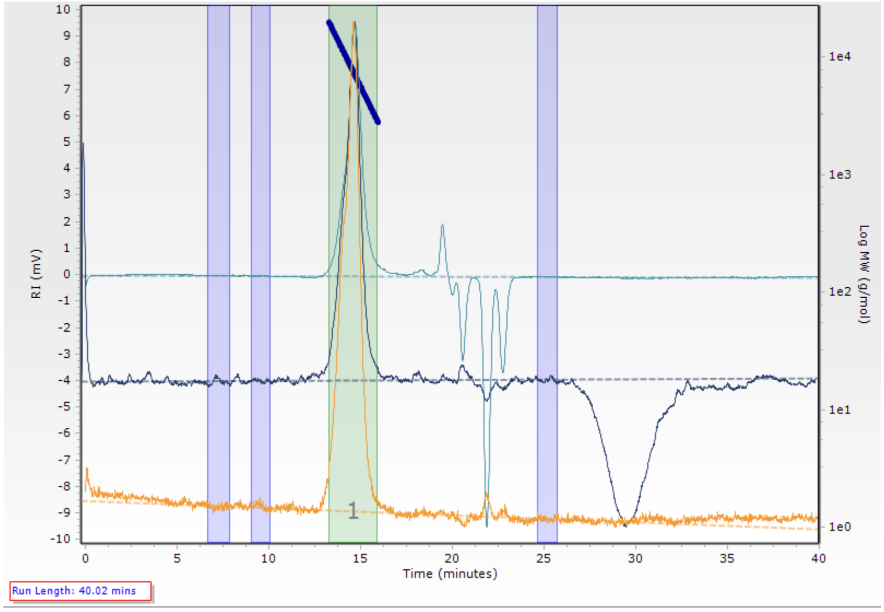

Figure S43. GPC trace for the polymer product of reaction IR-10; [Zr] = 1.95x10^{-2} mol%, [ROH] = 1.95 mol%, dosed as solid **1** with exogenous BnOH.

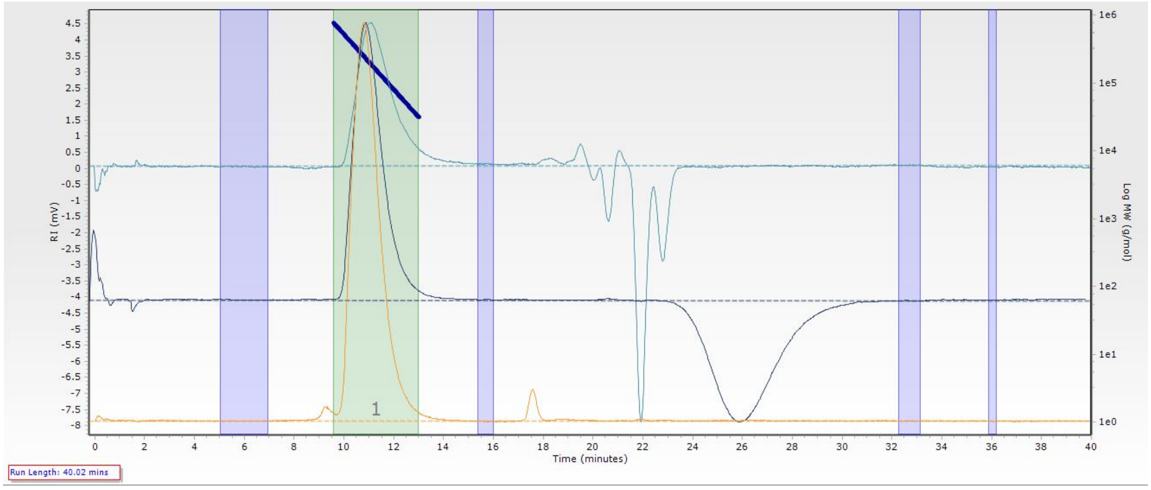

Figure S44. GPC trace for the polymer product of reaction L-2; [Zr] = 2.60x10^{-2} mol%, [BnOH] = 0.13 mol%, dosed as separate species, solid **1** and exogenous BnOH.

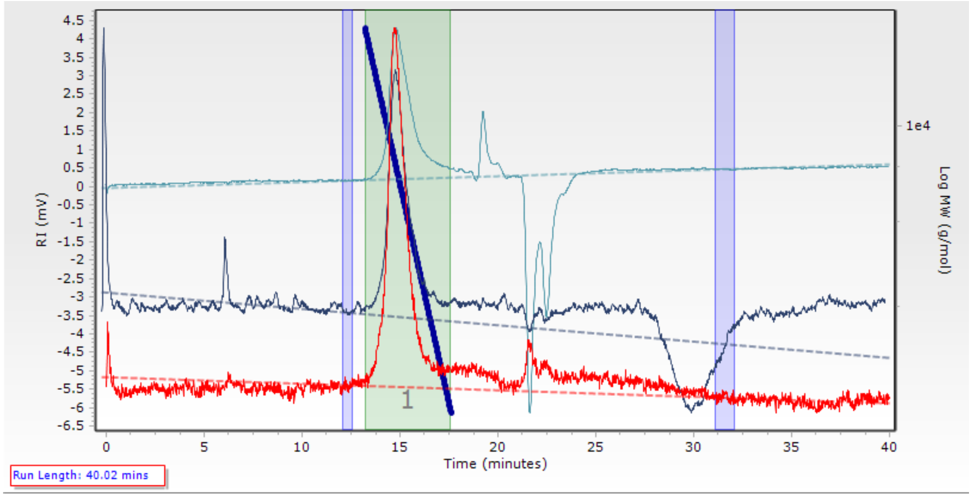

Figure S45. GPC trace for the polymer product of reaction IR-25; [Zr] = 1.3x10^{-2} mol%, [ROH] = 1.30 mol% dosed as formulation ***f1***, further 0.8 mol% aliquots of exogenous BnOH added at 4, 8, 12 and 16 minutes. The corresponding tail to low molecular weight is visible.

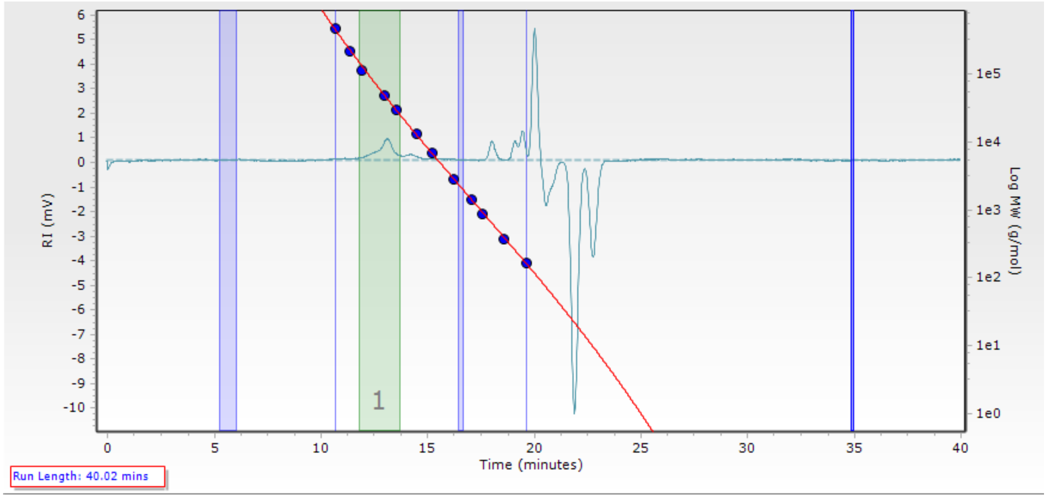

Figure S46. GPC trace for the polymer product of reaction IE-1, showing a bimodal molecular weight distribution, corresponding to two separate alcohol (and catalyst) addition events. [Zr] = 2.9x10^{-3} mol% + 1.5x10^{-3} mol%, [ROH] = 0.29 mol% + 0.15 mol%, dosed as ***f1***

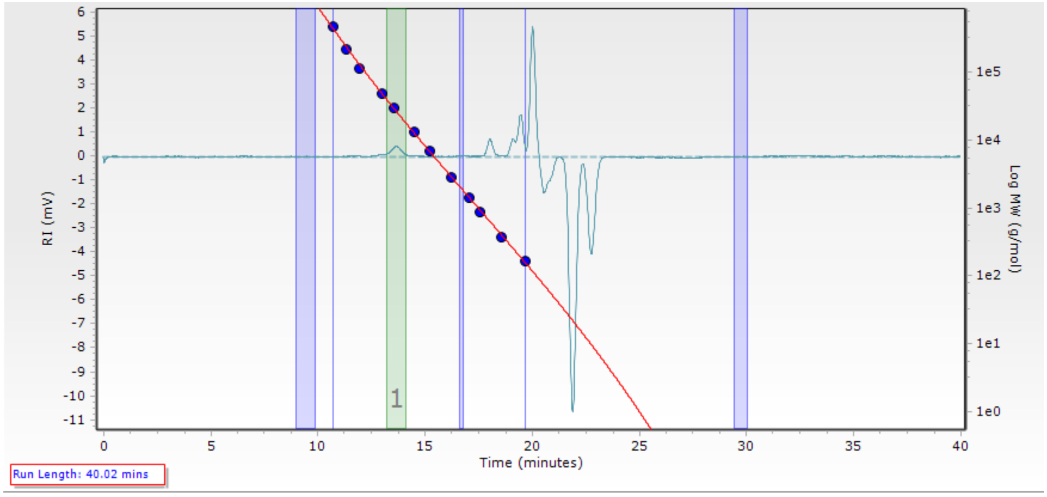

Figure S47. GPC trace for the sample of the reaction mixture drawn from reaction IE-1 after 90 minutes, showing a monomodal molecular weight distribution, prior to the second alcohol addition event. [Zr] = 2.9x10^{-3} mol% [ROH] = 0.29 mol%, dosed as ***f1***

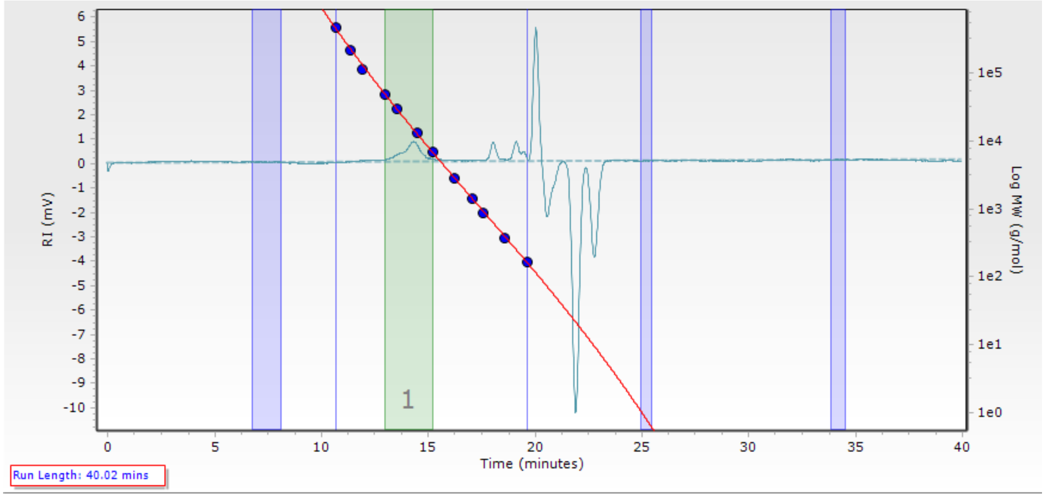

Figure S48. GPC trace for the polymer product of reaction IE-2. [Zr] = 8.1x10^{-3} mol%, [ROH] = 1.62 mol%, dosed as ***f3***

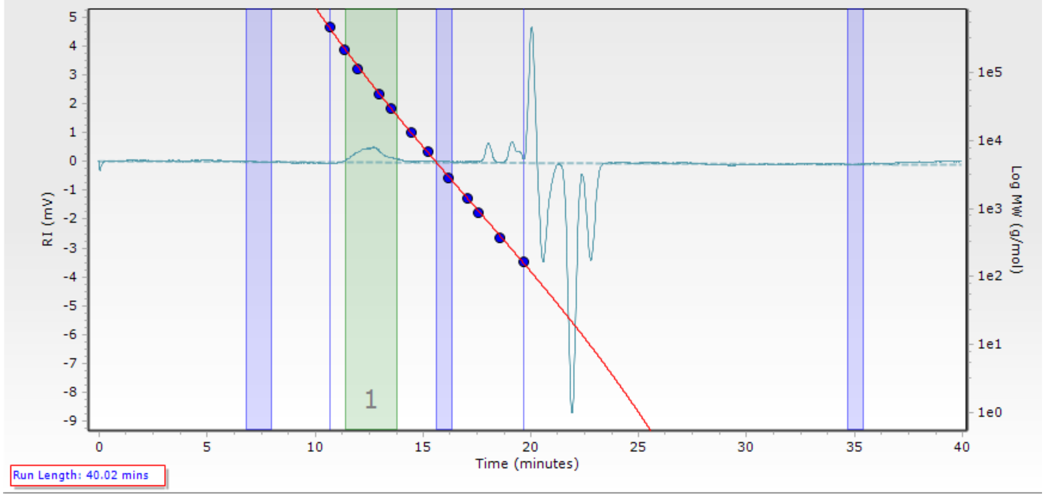

Figure S49. GPC trace for the polymer product of reaction IE-3. [Zr] = 1.5x10^{-3} mol%, [ROH] = 0.29 mol%, dosed as ***f3***

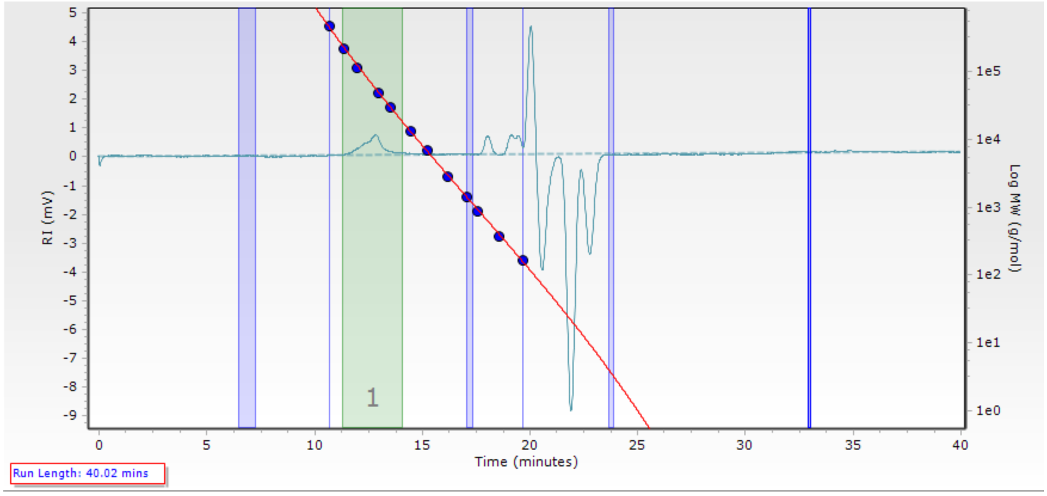

Figure S50. GPC trace for the polymer product of reaction IE-4. [Zr] = 1.9x10^{-3} mol%, [ROH] = 0.29 mol%, dosed as ***f2***

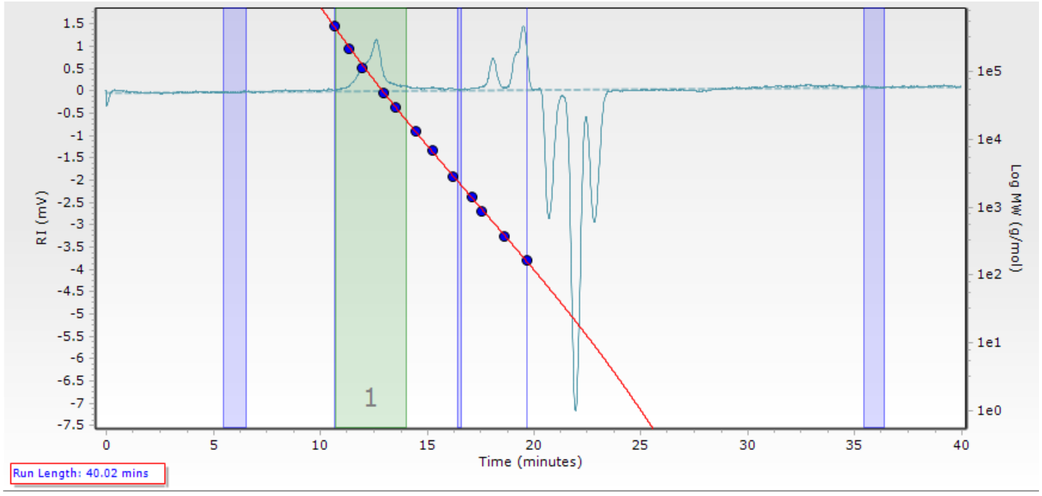

Figure S51. GPC trace for the polymer product of reaction IE-5. [Zr] = 1.3x10^{-3} mol%, [ROH] = 0.29 mol%, dosed as ***f2***

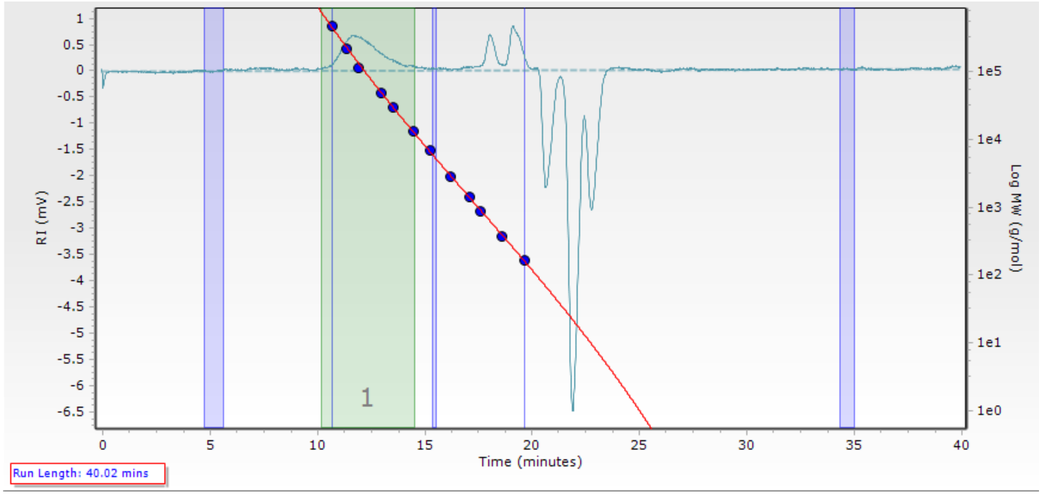

Figure S52. GPC trace for the polymer product of reaction IE-6, catalyzed by $Sn(Oct)_2$. [Sn] = 5.3x10^{-3} mol%, [Initiator] = 0.29 mol%

5 ROP of L-LA under conditions anticipated to promote side-reactions

Reactions L-1 and L-2 were undertaken to assess the resistance of **1** towards promotion of epimerization, causing deleterious racemisation of the polymer stereocenters. *L*-LA was polymerized in the presence of a high concentration of **1**, fewer equivalents of BnOH with respect to the catalyst, relative to ***f1***, and for an extended reaction time, in an effort to maximise proliferation of any undesirable side-reactions (see main paper). Under these testing conditions, no detectable epimerization occurred, entirely isotactic P*L*LA therefore being recovered in high yield. Conditions and polymer characterisation data are provided in Table S15.

Table S15. Polymerization data for the ROP of *L*-LA in the presence of solid **1** and BnOH

Entry	[c] Conversion, %	[d] M_n^{Theo}, g mol^{-1}	[e] M_n^{GPC}, g mol^{-1}	[e] $Đ_M$	[f] P_r
[a]*L-1*	96	11000	14200	1.49	~0
[b]*L-2*	96	106500	103400	1.56	~0

Conditions: 5 g of *L*-LA, solvent-free, 180 °C, 2 hours, solid **1** and BnOH used. [a] [Zr] = 2.60x10^{-2} mol%, [BnOH] = 1.3 mol% (160 ppm Zr by weight). [b] [Zr] = 2.60x10^{-2} mol%, [BnOH] = 0.13 mol% (160 ppm Zr by weight). [c] Conversion determined *via* ^{1}H NMR spectroscopy, by integration of monomer and polymer methine resonances. [d] M_n^{Theo} calculated from conversion and alcohol concentration, $\left\{\left(M_{r,LA} \times \frac{\%_{conv}}{100} \times \frac{[LA]}{[ROH.]}\right) + M_{r,BnOH}\right\}$. [e] Determined *via* GPC analysis in THF using Triple Detection. [f] P_r calculated *via* polymer microstructure analysis (^{1}H{^{1}H} NMR); $P_r = \sqrt{(2[sis])}$.[4]

5.1 ^{1}H NMR spectra of P*L*LA samples

Samples of P*L*LA were analysed *via* ^{1}H NMR spectroscopy. In the cases of reactions L-1 and L-2, homonuclear decoupling refers to broadband decoupling of the methine signal of the polymer backbone, δ_H = 5.16 ppm from the adjacent methyl group. In the current work, this treatment produced a large singlet, corresponding to the *iii* tetrad (Figure S53, Figure S54). No significant signals corresponding to other tetrads were observed, consistent with highly isotactic PLA, and negligible epimerization activity.[4]

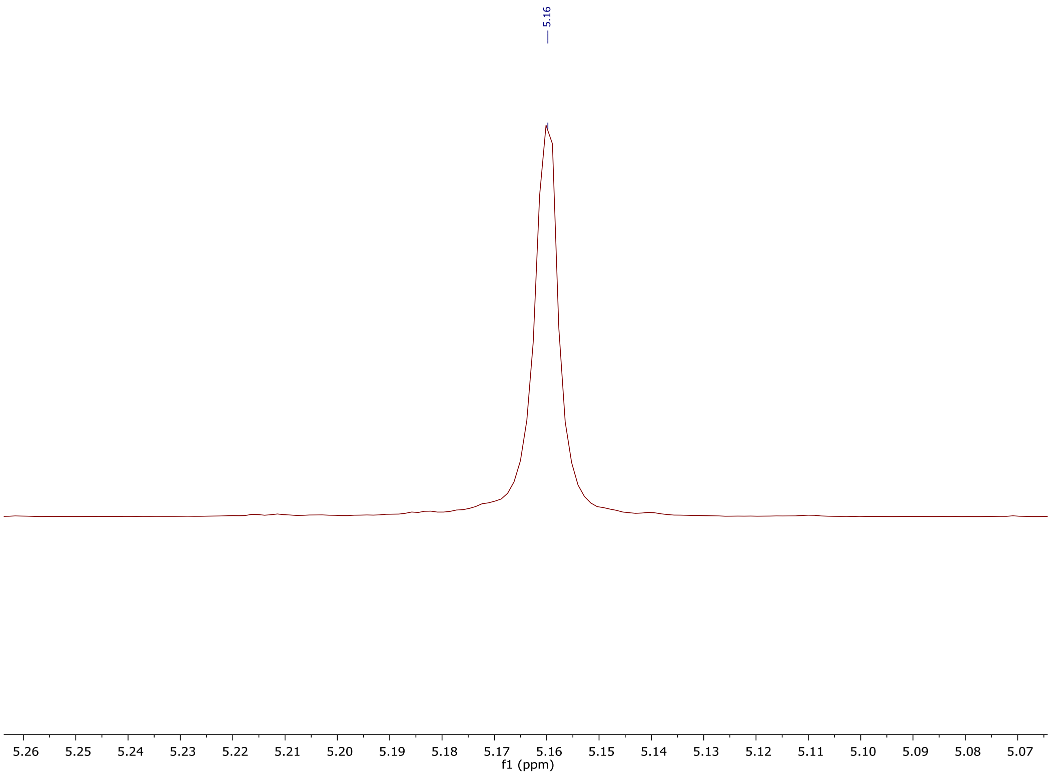

Figure S53. Homonuclear decoupled ^{1}H NMR signal corresponding to the methine protons of the polymer backbone of the P*L*LA product of polymerization L-1

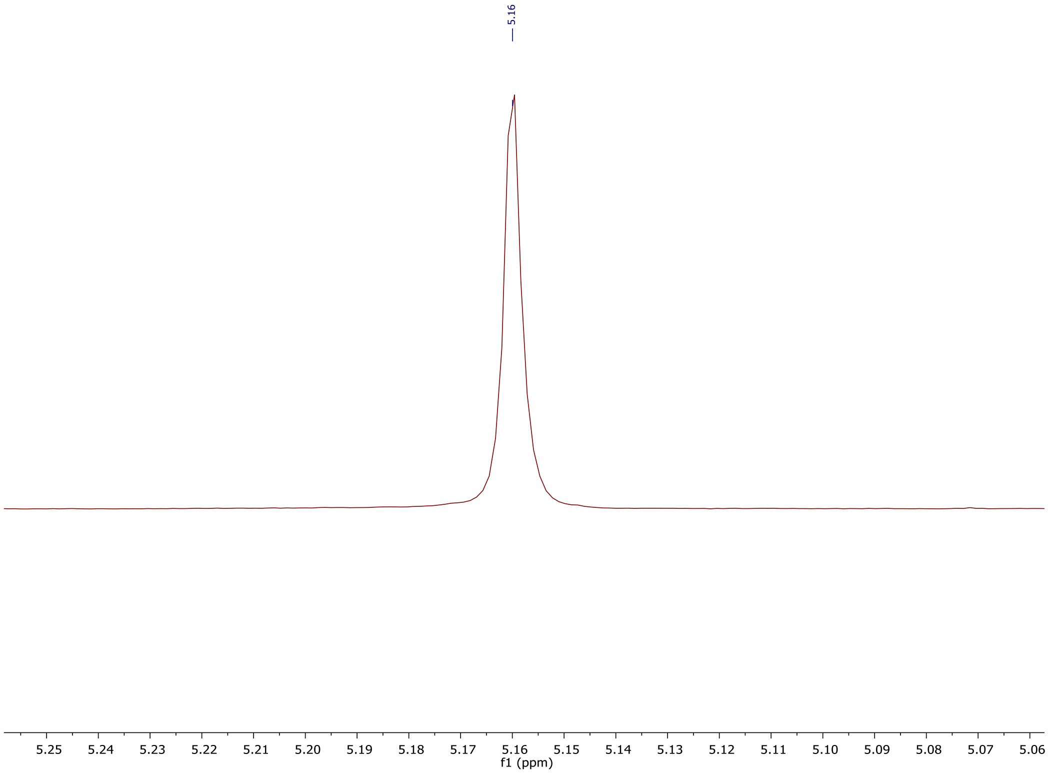

Figure S54. Homonuclear decoupled ^{1}H NMR signal corresponding to the methine protons of the polymer backbone of the P*L*LA product of polymerization L-2

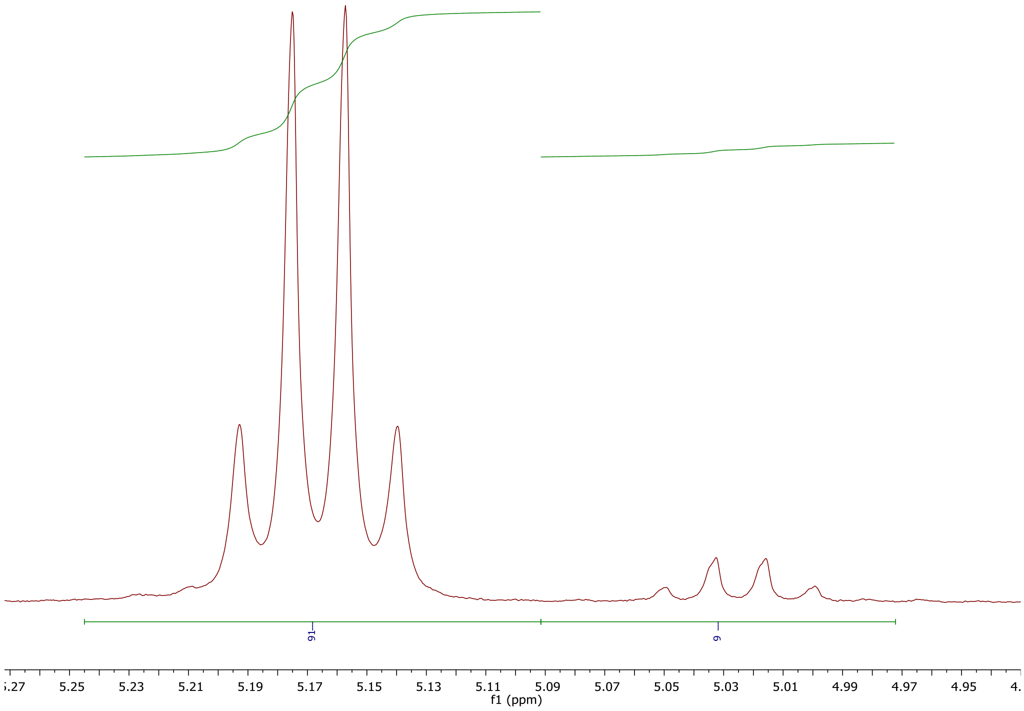

Figure S55. The methine region of the ^{1}H NMR spectrum of the PLA product of reaction IE-3 (without decoupling). The well-defined quartet at δ_H = 5.17 ppm corresponds to isotactic P*L*LA, with no evidence of epimerization after a 22.5-hour reaction time under industrially relevant conditions.

5.2 Differential Scanning Calorimetry of the crystallised product of reaction IE-3

Thermal analysis (DSC) of the crystalline pellets produced from the product of reaction IE-3 revealed the presence of a melting event with maximum negative heat flow occurring at 173.25˚C, consistent with literature values for isotactic P*L*LA of comparable molecular weight, and confirming the absence of epimerization.[10]

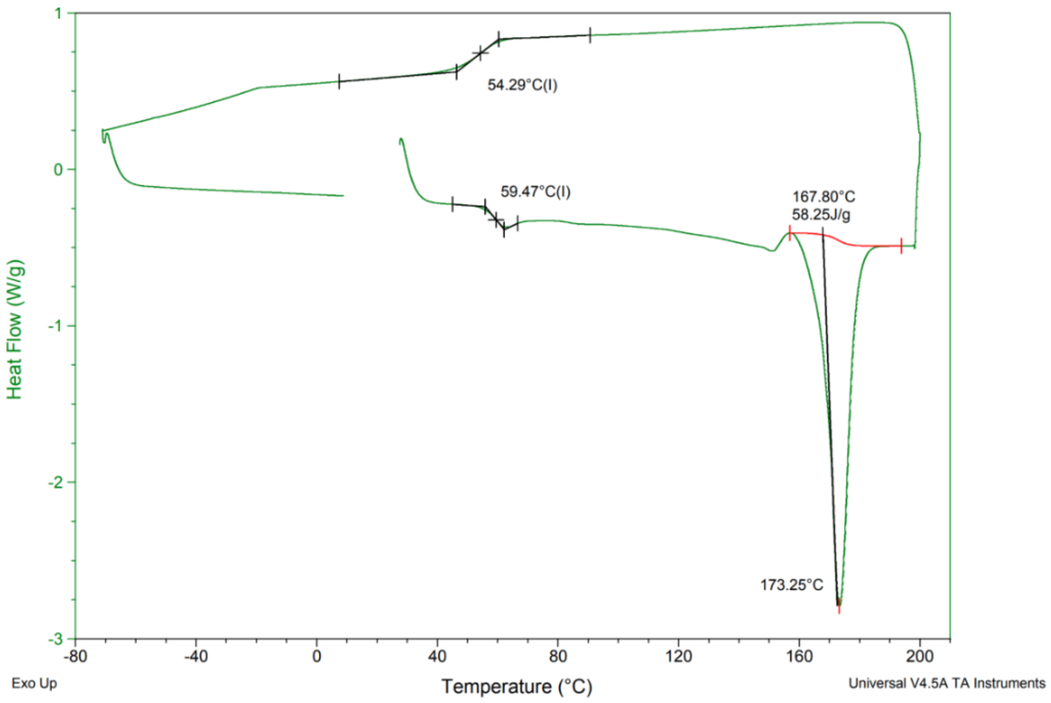

Figure S56. DSC data corresponding to the crystalline P*L*LA product of reaction IE-3.

6 Structural data for complex 2, Zr(HL$^{Me/tBu}$)$_2$

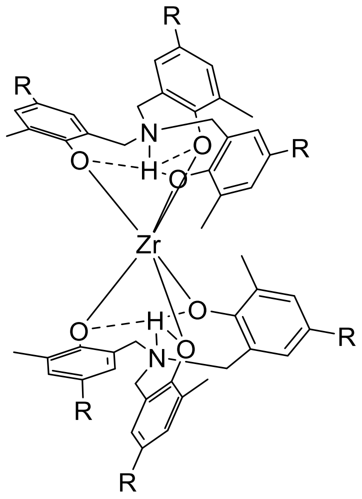

Zr(HL^{Me})$_2$, **1** : R=Me

Zr(HL$^{Me/tBu}$)$_2$, **2**: R=^{t}Bu

Figure S57. Molecular structures of isostructural complexes **1** and **2**. Complex **2** is discussed in the Supporting Information only.

6.1 General Procedure

Complex **2** (Figure S57), prepared in the current work to assess the effect of *tert*-butyl functionalisation of the ligand framework on the solubility of the catalyst in non-polar media, has previously been reported by Tasker and co-workers.[2] Although **2** was not applied to the ROP of lactides, a solid state structure for this species was obtained for the first time in the current work, and is therefore presented here (Figure S58).

Consistent with the greater thermal stability of **2** than **1** (see above), the Zr-O bond lengths of **2** were found to be shorter than those of **1** (2.0576(14) Å, 2.0494(13) Å, 2.0490(14) Å, versus 2.064(2) Å, 2.058(2) Å, 2.057(2) Å).[3]

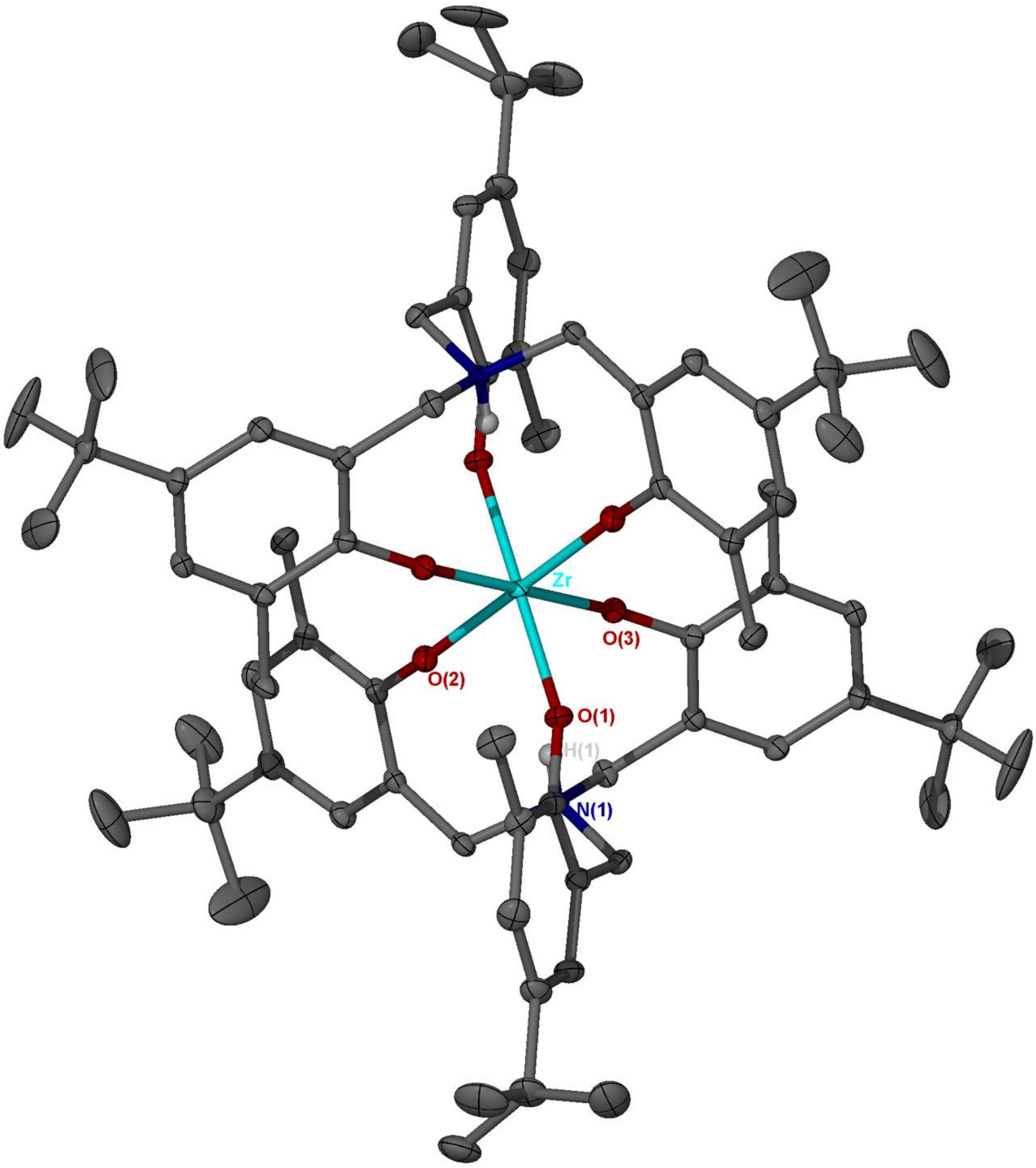

Figure S58. The solid-state structure of complex **2**, Zr($HL^{Me/tBu}$)$_2$. All ellipsoids are shown at the 30 % probability level and H atoms have been omitted, except for those bonded to the N atoms. Selected bond lengths (Å): Zr(1)-O(1) 2.0576(14), Zr(1)-O(2) 2.0490(14), Zr(1)-O(3) 2.0494(13).

6.2 Crystallographic parameters for complex 2, Zr($HL^{Me/tBu}$)$_2$

Crystal data for **2**: /, M = 1639.41 g/mol, colorless cube, 0.150 x 0.100 x 0.050 mm, space group P-1. Triclinic, a = 14.1647(15) Å, b = 14.3981(11) Å, c = 14.4928(13) Å, α = 83.050(7) °, β = 62.297(10)°, γ = 66.040(10)° V = 2383.0(5) Å^3, Z = 1, D_c = 1.142 g/cm^3, F_{000} = 882, SuperNova, Dual, EosS2, Cu Kα radiation, λ = 1.54184 Å, T = 150.01(10)K, 2θ_{max} = 147.1º, 18576 reflections collected, 9413 unique (R_{int} = 0.0339). Final $GooF$ = 1.041, R_1 = 0.0426, wR_2 = 0.1133, R indices based on 9001 reflections with I > 2σ(I) (refinement on F^2), 802 parameters, 388 restraints, μ = 1.350 mm^{-1}.

CCDC Deposition Number: 2206271

7 Computational analyses

We reproduce here the section on computational analysis presented in the main text, with addition of schemes corresponding to all evaluated scenarios, and other useful discussion.

Computational methods, specifically density functional theory (DFT), were applied to the mechanistic analysis of the ROP of LA in the presence of **1** and BnOH, due to the catalyst's apparent structural incompatibility with standard coordination-insertion or activated monomer mechanisms, and the difficulty of experimentally delineating a mechanistic pathway under the conditions of use.

Due to the size of the catalytic system (involving up to 159 atoms in the initiation step), a layered basis set protocol (6-311+g(d) for N and O atoms, 6-311++g(d,p) for H from NH and OH groups, 6-31g(d) for all C and H atoms, except CH_3 groups on aromatic rings (STO-3G)), involving the Stuttgart/Dresden effective core potential (SDD ECP) and associated basis set for the zirconium center, was used.

Calculations were initially carried out using PBE0 functional augmented with Grimme's empirical dispersion correction D3.[11] to PBE0 functional is a hybrid functional, which mixes the Perdew–Burke-Ernzerhof (PBE) exchange energy and Hartree-Fock exchange energy (25%), along with the full PBE correlation energy.[12] It had been repeatedly found to generate good geometries for transition metal complexes, and performed well for Zr-mediated reaction barriers in benchmark studies (with Mean Unsigned Deviations (MUD) less than 2 kcal/mol).[13] To best approximate solvation in molten LA and PLA, the solvent phase was modelled as ethyl acetate, using a self-consistent reaction-cavity continuum solvation model (cpcm).[14,15] All calculations were undertaken using a temperature of 453.15 K. The transition state for the rate determining step was subsequently recalculated at 447.15 K, to ensure parity with the experimental conditions, and no significant difference in the activation barriers was observed.

All geometries were fully optimized without any symmetry or geometry constrains. The nature of all the stationary points as minima or transition states was verified by calculations of the vibrational frequency spectrum. All transition states were characterized by precisely one imaginary mode corresponding to the intended reaction.

Full coordinates for all the stationary points, together with computed free Gibbs energy and vibrational frequency data, are available via the corresponding Gaussian 09 output files, stored in the digital repository: DOI: 10.6084/m9.figshare.21078343.

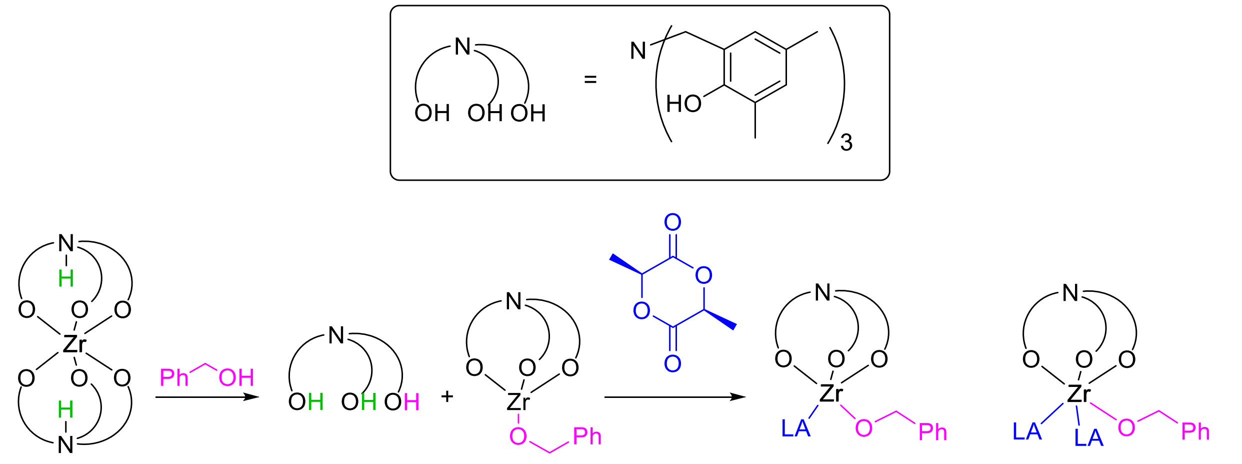

1 **II**

ΔG = 0.0 kcal mol^{-1} ΔG = +45.8 kcal mol^{-1} ΔG = +50.7 kcal mol^{-1} ΔG = +67.8 kcal mol^{-1}

Scheme S2. Energetically inaccessible ligand-dissociation and zirconium benzyl alkoxide formation

Initiation of a coordination-insertion mechanism would require protonation of one amine tris(phenolate) ligand on treatment with BnOH, to yield a heteroleptic Zr alkoxide complex [L^{Me}ZrOBn], **II** (Scheme S2). Formation of this species was calculated to be very unfavorable; ΔG = +45.8 kcal mol^{-1}. The same species was also modelled with one and two equivalents of *L*-LA coordinated to the metal center. However, formation of these adducts was not more favorable; ΔG = +50.7 kcal mol^{-1} and ΔG = 67.8 kcal mol^{-1}, respectively. Thermogravimetric analysis has shown that under inert conditions, **1** is stable to 225 °C. Additionally, when a 1 mol% solution of **1** in BnOH, was heated to 200 °C for 30 minutes, only partial decomposition was observed. The persistence of some of the catalyst under such conditions is not compatible with the rapid initiation of an immortal ROP process involving decomposition *via* ligand loss. That pathway was disregarded.

Further computational work therefore considered two mechanistic scenarios in which both ligands remained *in-situ* at the metal center:

1. Formation of a benzyl alkoxide complex, **IV**, by protonation of one phenolate ‘arm’, followed by a coordination-insertion mechanism (Pathway A, Scheme S3).
2. An activated monomer mechanism wherein the Lewis acidic metal center of **1** activates the monomer carbonyl moiety.

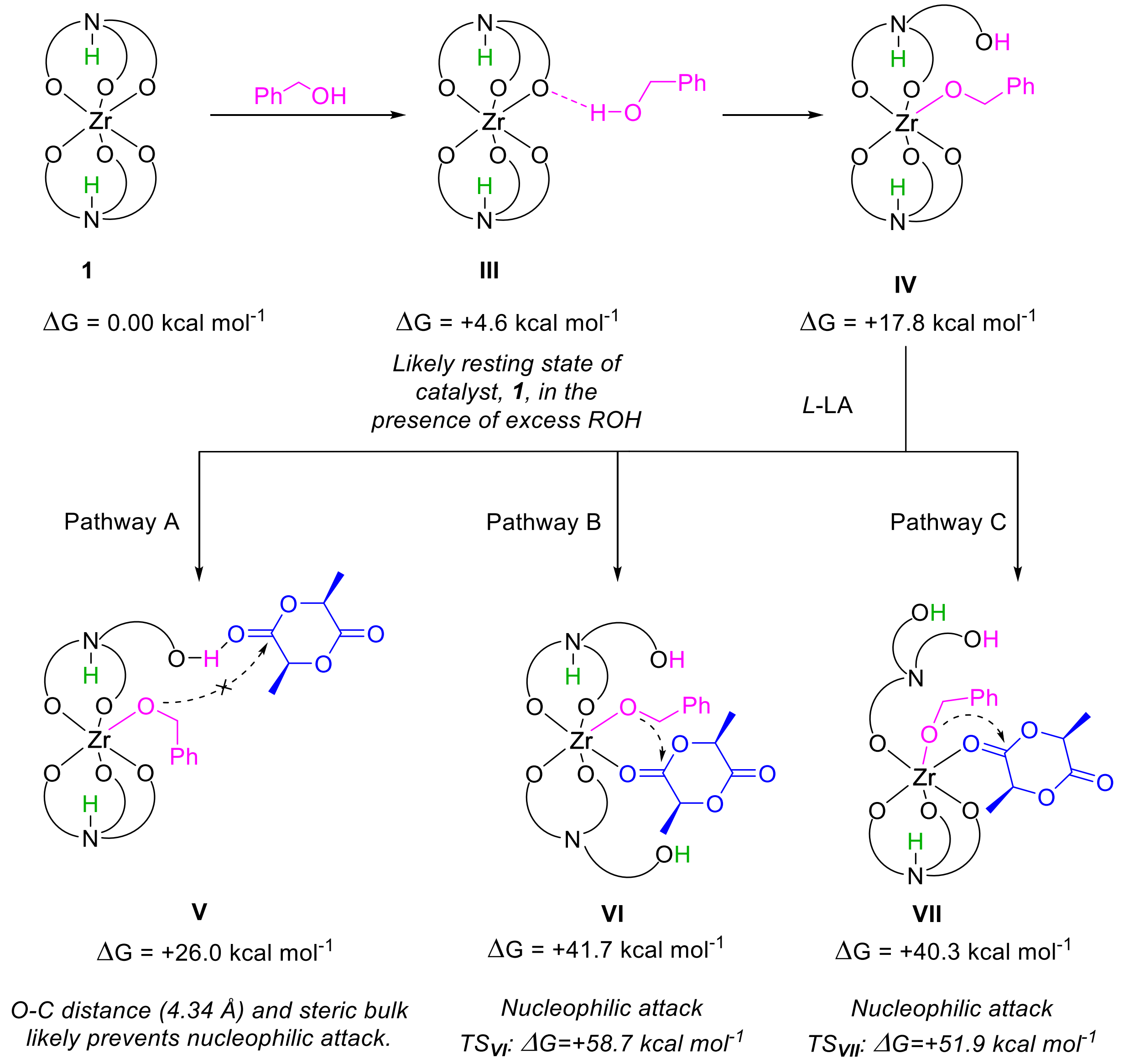

Scheme S3. Ligand arm-dissociation to form alkoxide complex, 4, and various subsequent mechanistic scenarios

The formation of a benzyl alkoxide, **IV**, as described in the first scenario, was considered to proceed first by the alcohol hydrogen bonding to the phenolate, followed by proton transfer and Zr-alkoxide bond formation (Scheme S3). Formation of the hydrogen bonded binary complex, **III**, is only slightly unfavorable; ΔG = +4.6 kcal mol^{-1}. This is expected to be the resting state of the catalyst in the presence of excess alcohol, such as under the experimental conditions we describe. The subsequent alkoxide complex, **IV**, is disfavored but accessible; ΔG = +17.8 kcal mol^{-1}. From **IV**, several possibilities were explored. Activation of the monomer through hydrogen bonding to the protonated ligand arm, proceeding via intermediate **V** (Pathway A, Scheme S3) was considered unfeasible due to the excessive distance between nucleophile and monomer, enforced by steric congestion. Activation of the monomer *via* such a hydrogen bonding interaction was also considered unlikely.

Another possibility, described by the second scenario, was dissociation of an arm of the second tripodal ligand (Pathway B, Scheme S3), permitting coordination of LA to the vacant site to form intermediate **VI**. Dissociation in that case would entail transfer of the corresponding zwitterionic NH^+ proton to the phenolate arm. This was highly unfavorable. For formation of the *L*-LA complex, ΔG = +45.1 kcal mol^{-1}, and the barrier to nucleophilic attack at the monomer by the alkoxide was determined to be $\Delta G^‡$ = +58.7 kcal mol^{-1}. These values are considered insurmountable under the current conditions. Finally, dissociation of a second phenolate arm from the first ligand (Pathway C, Scheme S3), *via* transfer of the respective NH^+ proton, and coordination of LA at the vacant site, was considered. Formation of that LA complex, **7**, was considered highly unfavorable, and the barrier to nucleophilic attack by the alkoxide was again considered insurmountable; ΔG = +40.3 kcal mol^{-1} and $\Delta G^‡$ = +51.9 kcal mol^{-1}, respectively.

It was also necessary to consider a classical Lewis-acid catalyzed activated monomer mechanism, without dissociation of a phenolate arm. As expected, DFT modelling showed that due to the coordinative saturation and extensive steric congestion about the Zr center of **1**, interaction with the monomer to form binary complex **VIII**, (Scheme S4) was entirely obstructed.

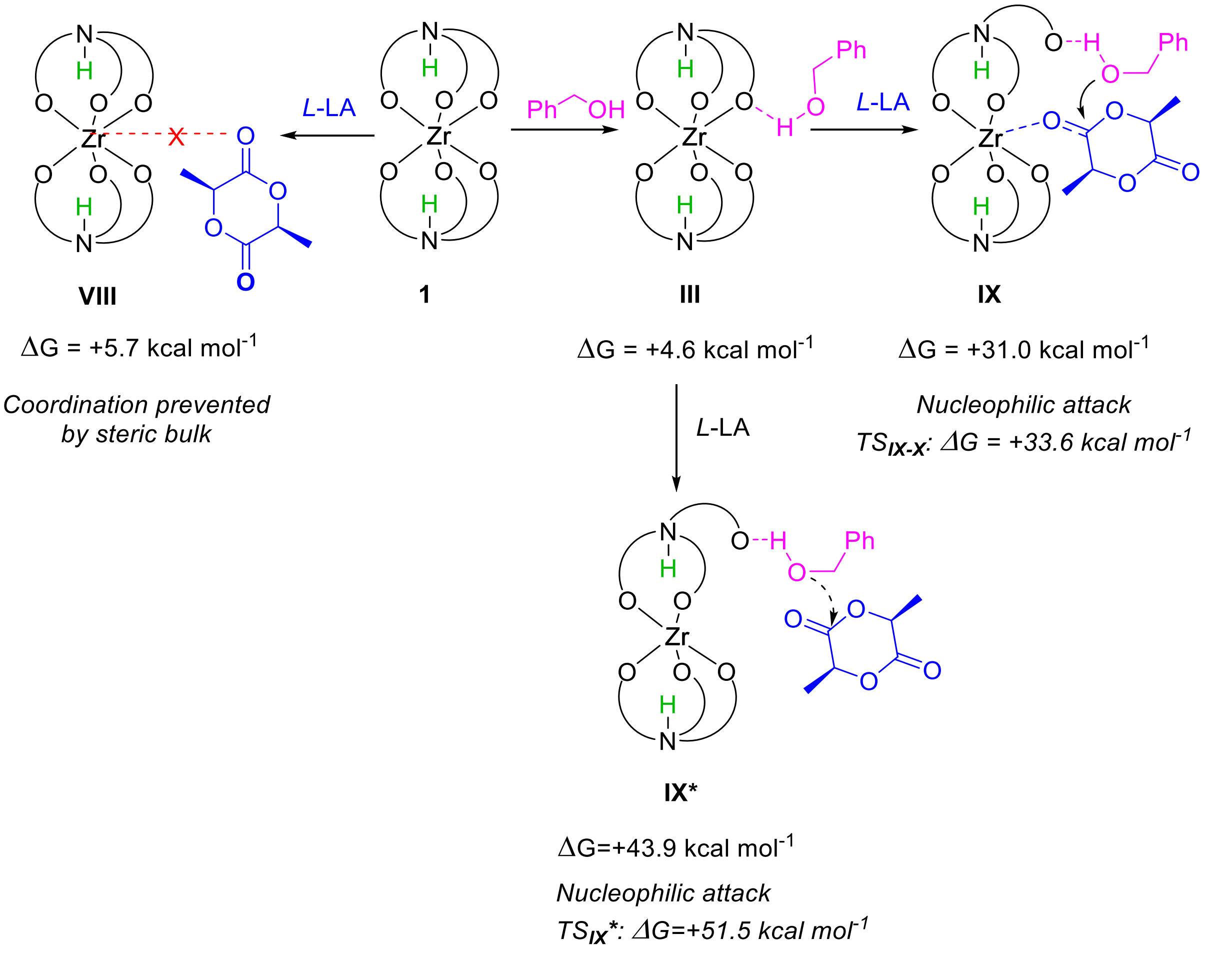

Scheme S4. Evaluated mechanistic scenarios for activated monomer-type mechanistic pathways

From the likely resting state of the catalyst, a binary complex of **1** and BnOH, **III**, dissociation of a ligand arm may occur in the presence of LA without formation of a Zr-benzyl alkoxide complex. Stabilization of the anionic phenolate group may occur by hydrogen bonding to BnOH, which is partially deprotonated (extension of O-H bond from 0.96103 Å in BnOH alone to 1.00281 Å), with concurrent occupation of the vacant metal coordination site by a LA molecule, to form intermediate **IX** (Scheme 4). The carbonyl group of the monomer is therefore activated (extension of C=O bond from 1.19855 Å in *L*-LA alone to 1.22206 Å). Whilst this is less favorable than formation of the Zr-benzyl alkoxide species, **IV** (Scheme S3), it is considered accessible; $\Delta G = +31.0$ kcal mol^{-1}. Furthermore, the high concentration of LA under solvent-free polymerization conditions is likely to favor this pathway.

The simultaneous activation of alcohol and monomer species facilitates nucleophilic attack at the monomer carbonyl group, with a transition state energy of $\Delta G^{\ddagger} = +33.6$ kcal mol^{-1}. This is considered accessible under the polymerization conditions, and is lower than for all alternative pathways. This activity corresponds to a ligand assisted activated monomer mechanism, reminiscent of that reported by Carpentier and co-workers, which has not been previously reported for an industrially relevant system.[16]

When the system was modelled without the metal-LA interaction (**IX***, Scheme S4), nucleophilic attack by the activated alcohol was very inaccessible, with a transition state energy of $\Delta G^{\ddagger} = +51.5$ kcal mol^{-1}. Dissociation of the ligand arm from the metal center, and hydrogen bonding to the alcohol, without occupation of the vacant metal coordination site, is also highly unfavorable; $\Delta G = +43.9$ kcal mol^{-1}.

Following nucleophilic attack on the activated LA molecule (**IX**, Scheme S4), the resulting quaternary species, **X** (Scheme S5), ($\Delta G = +22.3$ kcal mol^{-1}) can undergo ring opening to yield a free alcohol chain hydrogen bonded to the dissociated ligand arm to give structure **XI**, *via* transition state $TS_{X\text{-}XI}$; $\Delta G^{\ddagger} = +39.6$ kcal mol^{-1}. This was calculated to be much more favorable than ring-opening with retention of the Zr-O bond to yield a metal alkoxide chain, to give structure **XII** through rotation of the monomer to give intermediate **X***, then *via* transition state $TS_{X\text{-}XII}$; $\Delta G^{\ddagger} = +67.6$ kcal mol^{-1}. Intermediate **XI** is additionally stabilized by coordination of the benzyl lactate carbonyl moiety to the Zr center, reminiscent of intermediate **IX**.

Finally, coordination of the ligand arm back to the metal center is favorable, yielding a binary complex of **1** and the growing chain (alcohol), **XIII**, ($\Delta G = +6.7$ kcal mol^{-1}), analogous to intermediate **III**. This is anticipated to be the resting state of the catalyst during propagation, and is clearly relevant to the subsequent addition of further equivalents of the monomer to the

growing chain. Decomposition of the binary complex to yield the free alcohol chain and catalyst **1** has been calculated to be thermodynamically neutral; ΔG = 0.2 kcal mol^{-1}.

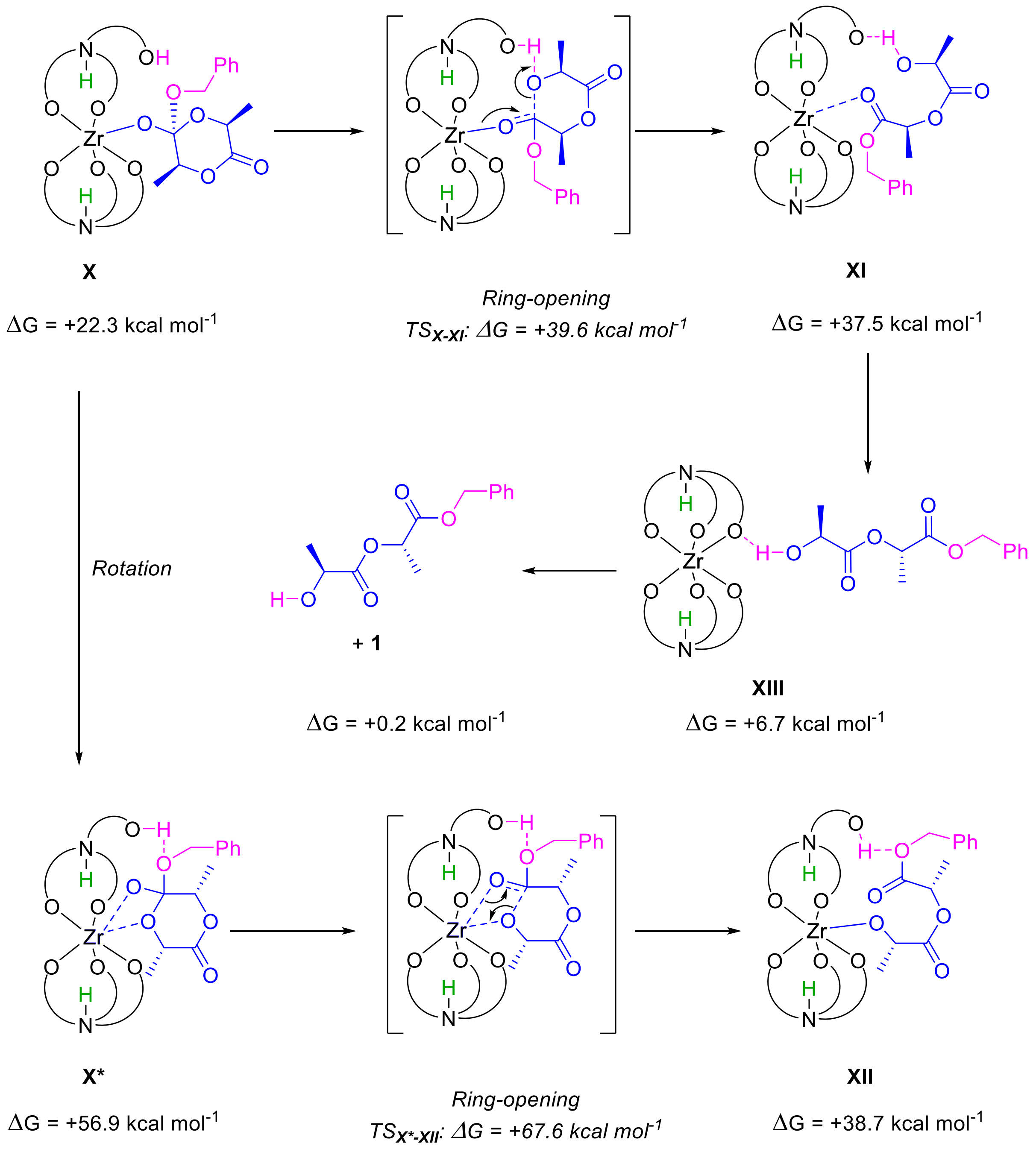

Scheme S5. Energetically inaccessible ligand-dissociation and zirconium benzyl alkoxide formation

Such slight endergonicity in the modelling of LA ROP has been previously observed by Gibson and Rzepa,[17,18] among others.[19] As only the initiation step was modelled, the limiting free energy of the ROP process has not necessarily been accurately represented. Indeed, with an increased number of

monomer units, the entropy of the system is anticipated to increase and the free enthalpy of polymerization is therefore expected to become more favorable as polymerization progresses, in agreement with the experimental value of ΔG‡. Accordingly the Gibbs Free Energy profile for the initiation step, using the PBE0-D3 protocol is shown in Figure S59, and propagation is expected to be mechanistically analogous, commensurate with **1** being a catalyst, rather than a pre-catalyst. Because the mechanistic pathway has been delineated based on comparison of calculated activation barriers, all transition states were re-optimized and computed using different functionals (ωB97XD and M06-D3) for comparison (Table S16, Figure S60). Those calculations further support the proposed LAAM ROP mechanism.

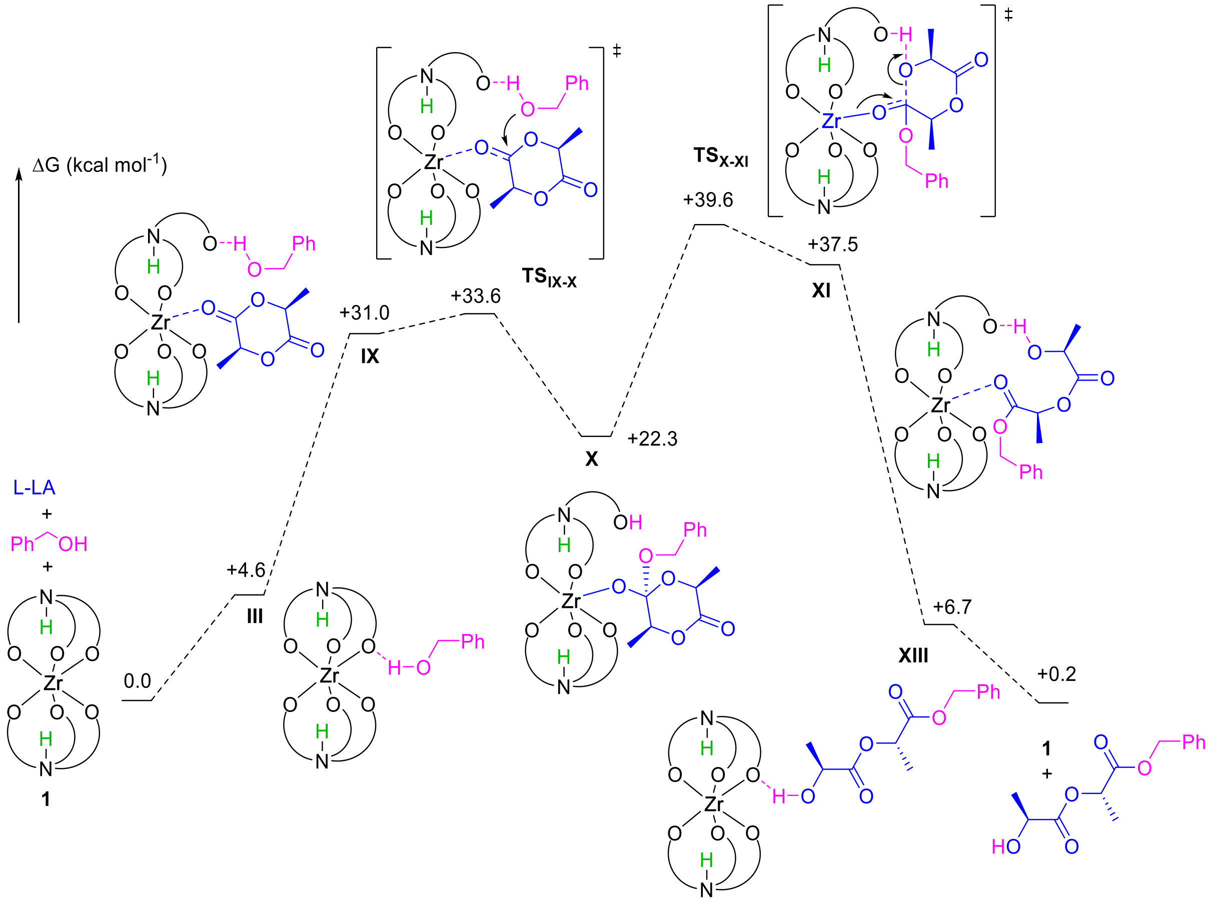

Figure S59. Energetic profile for the favored mechanism of ROP calculated using the PBE0-D3 protocol

Table S16. Transition state energies for the favored mechanism calculated with PBE0-D3, ωB97XD and M06-D3 protocols for comparison

Protocol	*PBE0-D3*	*ωB97XD*	*M06-D3*
Transition State (kcal mol⁻¹)			
TS_{VI}	58.7	63.9	60.9
TS_{VII}	51.9	54.7	52.1
TS_{IX*}	51.5	58.2	61.2
TS_{IX-X}	**33.6**	**41.0**	**32.2**
TS_{X-XI}	**39.6**	**42.0**	**38.1**
TS_{X*-XII}	67.6	70.7	69.4

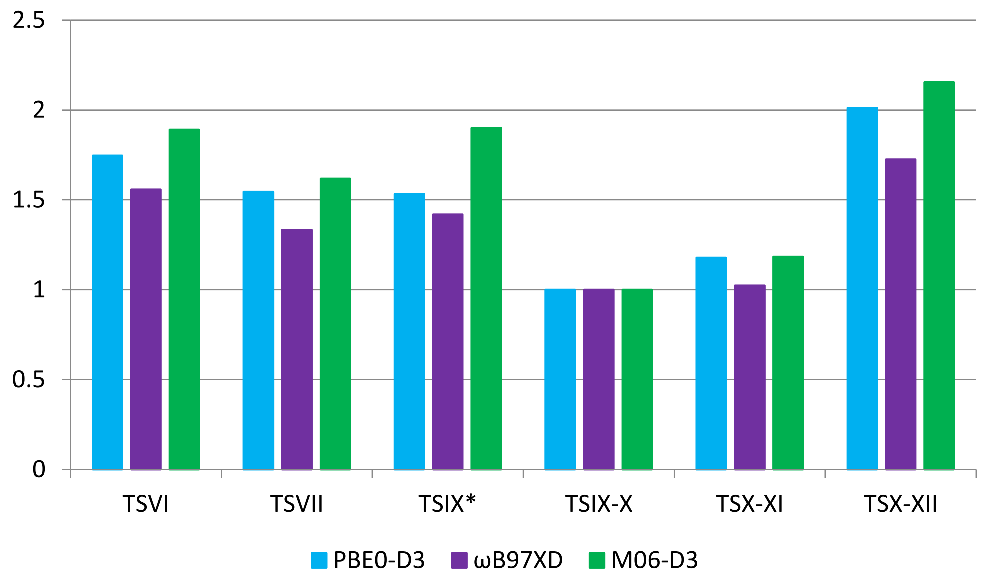

Figure S60. Normalized free Gibbs energies of the transition states optimized and computed using various functionals, relative to TS_{IX-X}

All energetic barriers calculated at 180 °C were considered high compared to values found in the literature for ROP processes. However, transition state theory modelling indicated that mechanistic events with values below 40 kcal mol^{-1} were accessible under the current conditions. Furthermore, the experimentally-determined $\Delta G^{\ddagger}$ value for the ROP of LA in the presence of **1**, $\Delta G^{\ddagger}$ = 32.5 kcal mol^{-1}, was similarly high. Finally, all transition states were re-optimized and computed at 25 °C (Table S17). The lowest-energy transition states and the favorability of the LAAM mechanism were retained at that temperature. The limiting activation barrier for the LAAM pathway at 25 °C was ΔG = +24.5 kcal mol^{-1}, consistent with literature values for other ROP processes at the same temperature.

Table S17. Transition state energies for the favored mechanism calculated with PBE0-D3 at 180 °C and 25 °C

Protocol	*PBE0-D3 180 °C*	*PBE0-D3 25 °C*
Transition State (kcal mol^{-1})		
TS_{VI}	58.7	44.8
TS_{VII}	51.9	36.1
TS_{IX*}	51.5	37.5
TS_{IX-X}	**33.6**	**19.5**
TS_{X-XI}	**39.6**	**24.5**
TS_{X*-XII}	67.6	51.1

Table S18. Computed Gibbs Free Energies at the PBE0-D3/cpcm=ethylacetate/453.15K level of theory for various mechanisms possible for the ring-opening of *L*-lactide catalyzed by **1** and initiated by benzyl alcohol (*vide supra* for layered basis set details).

	Structure	G (Hartree)	ΔG (kcalmol-1)
Starting materials	**1**	-2694.213762	
	Benzyl alcohol	-346.324725	
	L-Lactide (LA)	-533.808656	
Ligand-dissociation and zirconium alkoxide complex formation	$L^{Me}H_3$	-1324.600048	
	**II**	-1715.865517	
	**1** + BnOH	-3040.538487	0.0 (reference)
	**II** + $L^{Me}H_3$	-3040.465565	+45.8
	**II(LA)**	-2249.666361	
	**1** + BnOH +LA	-3574.347143	0.0 (reference)
	**II(LA)** + $L^{Me}H_3$	-3574.266409	+50.7
	**II(LA)₂**	-2249.666361	
	**1** + BnOH +2LA	-3574.347143	0.0 (reference)
	**II(LA)₂** + $L^{Me}H_3$	-3574.266409	+67.8
Ligand arm(s) dissociation to form alkoxide	**1** + BnOH	-3040.538487	0.0 (reference)
	**III**	-3040.531229	+4.6
	**IV**	-3040.510107	+17.8
	**1** + BnOH +LA	-3574.347143	0.0 (reference)
Pathway A	**V**	-3574.305756	+26.0
Pathway B	**VI**	-3574.280616	+41.7
	**TS$_{VI}$**	-3574.253601	+58.7
Pathway C	**VII**	-3574.282875	+40.3
	**TS$_{VII}$**	-3574.264473	+51.9
Direct coordination of LA to **1**	**1** + LA	-3228.022418	0.0 (reference)
	**VIII** (no coordination of LA to Zr)	-3228.013329	+5.7
LAAM mechanism	**1** + BnOH +LA	-3574.347143	0.0 (reference)
	**IX**	-3574.297654	+31.0
	**TS$_{IX-X}$**	-3574.293624	+33.6
	**IX***	-3574.277222	+43.9
	**TS$_{IX*}$**	-3574.265127	+51.5
	**X**	-3574.311622	+22.3
	**TS$_{X-XI}$**	-3574.284033	+39.6
	**XI**	-3574.287426	+37.5
	**X***	-3574.256391	+56.9
	**TS$_{X*-XII}$**	-3574.239480	+67.6
	**XII**	-3574.285446	+38.7
	**XIII**	-3574.336389	+6.7
	BnO(LA)H (LA opened by BnOH)	-880.132988	
	**1** + BnO(LA)H	-3574.346750	+0.2
Influence of selected functional on TS barriers			
ωB97XD	**1**	-2696.422776	
	Benzyl alcohol	-346.610174	
	L-Lactide (LA)	-534.222474	
	**1** + BnOH +LA	-3577.255424	0.0 (reference)
	**TS$_{VI}$**	-3577.153666	+63.9
	**TS$_{VII}$**	-3577.168175	+54.7
	**TS$_{IX*}$**	-3577.162709	+58.2
	**TS$_{IX-X}$**	-3577.190135	+41.0
	**TS$_{X-XI}$**	-3577.188441	+42.0
	**TS$_{X*-XII}$**	-3577.14275	+70.7
M06-D3	**1**	-2695.091448	
	Benzyl alcohol	-346.471012	
	L-Lactide (LA)	-534.082000	
	**1** + BnOH +LA	-3575.64446	
	**TS$_{VI}$**	-3575.547465	+60.9

	**TS**$_{\mathbf{VII}}$	-3575.561379	+52.1
	**TS**$_{\mathbf{IX*}}$	-3575.546917	+61.2
	**TS**$_{\mathbf{IX\text{-}X}}$	-3575.59317	+32.2
	**TS**$_{\mathbf{X\text{-}XI}}$	-3575.58377	+38.1
	**TS**$_{\mathbf{X*\text{-}XII}}$	-3575.533893	+69.4
PBE0-D3 at T = 298.15K	**1**	-2694.113323	
	Benzyl alcohol	-346.301958	
	L-Lactide (LA)	-533.783229	
	**1** + BnOH +LA	-3574.19851	0.0 (reference)
	**TS**$_{\mathbf{VI}}$	-3574.12718	+44.8
	**TS**$_{\mathbf{VII}}$	-3574.141048	+36.1
	**TS**$_{\mathbf{IX*}}$	-3574.138811	+37.5
	**TS**$_{\mathbf{IX\text{-}X}}$	-3574.167449	+19.5
	**TS**$_{\mathbf{X\text{-}XI}}$	-3574.159416	+24.5
	**TS**$_{\mathbf{X*\text{-}XII}}$	-3574.117076	+51.1

References

(1) Groysman, S.; Segal, S.; Shamis, M.; Goldberg, I.; Kol, M.; Goldschmidt, Z.; Hayut-Salant, E. Tantalum(v) Complexes of an Amine Triphenolate Ligand: A Dramatic Difference in Reactivity between the Two Labile Positions. *J. Chem. Soc. Dalt. Trans.* **2002**, No. 18, 3425–3426. https://doi.org/10.1039/b206759e.

(2) Chartres, J. D.; Dahir, A.; Tasker, P. A.; White, F. J. Zirconium Complexes of Amino(Polyphenolic) Ligands and Their Hydrolytic Stability. *Inorg. Chem. Commun.* **2007**, *10* (10), 1154–1158. https://doi.org/10.1016/j.inoche.2007.06.004.

(3) Davidson, M. G.; Doherty, C. L.; Johnson, A. L.; Mahon, M. F. Isolation and Characterisation of Transition and Main Group Metal Complexes Supported by Hydrogen-Bonded Zwitterionic Polyphenolic Ligands. *Chem. Commun.* **2003**, No. 15, 1832. https://doi.org/10.1039/b303618a.

(4) Cheng, M.; Attygalle, A. B.; Lobkovsky, E. B.; Coates, G. W. Single-Site Catalysts for Ring-Opening Polymerization: Synthesis of Heterotactic Poly(Lactic Acid) from Rac-Lactide [9]. *J. Am. Chem. Soc.* **1999**, *121* (49), 11583–11584. https://doi.org/10.1021/ja992678o.

(5) Burés, J. A Simple Graphical Method to Determine the Order in Catalyst. *Angew. Chemie - Int. Ed.* **2016**, *55* (6), 2028–2031. https://doi.org/10.1002/anie.201508983.

(6) Burés, J. Variable Time Normalization Analysis: General Graphical Elucidation of Reaction Orders from Concentration Profiles. *Angew. Chemie - Int. Ed.* **2016**, *55* (52), 16084–16087. https://doi.org/10.1002/anie.201609757.

(7) Nomura, N.; Ishii, R.; Yamamoto, Y.; Kondo, T. Stereoselective Ring-Opening Polymerization of a Racemic Lactide by Using Achiral Salen- and Homosalen-Aluminum Complexes. *Chem. - A Eur. J.* **2007**, *13* (16), 4433–4451. https://doi.org/10.1002/chem.200601308.

(8) Wang, L.; Poirier, V.; Ghiotto, F.; Bochmann, M.; Cannon, R. D.; Carpentier, J. F.; Sarazin, Y. Kinetic Analysis of the Immortal Ring-Opening Polymerization of Cyclic Esters: A Case Study with Tin(II) Catalysts. *Macromolecules* **2014**, *47* (8), 2574–2584. https://doi.org/10.1021/ma500124k.

(9) Kowalski, A.; Duda, A.; Penczek, S. Kinetics and Mechanism of Cyclic Esters Polymerization Initiated with Tin(II) Octoate. 3. Polymerization of L,L-Dilactide. *Macromolecules* **2000**, *33* (20), 7359–7370. https://doi.org/10.1021/ma000125o.

(10) Shao, J.; Xiang, S.; Bian, X.; Sun, J.; Li, G.; Chen, X. Remarkable Melting Behavior of PLA Stereocomplex in Linear PLLA/ PDLA Blends. **2015**. https://doi.org/10.1021/ie504484b.

(11) Grimme, S.; Antony, J.; Ehrlich, S.; Krieg, H. A Consistent and Accurate Ab Initio Parametrization of Density Functional Dispersion Correction (DFT-D) for the 94 Elements H-Pu. *J. Chem. Phys.* **2010**, *132* (15), 154104. https://doi.org/10.1063/1.3382344.

(12) Adamo, C.; Barone, V. Toward Reliable Density Functional Methods without Adjustable Parameters: The PBE0 Model. *J. Chem. Phys.* **1999**, *110* (13), 6158. https://doi.org/10.1063/1.478522.

(13) Sun, Y.; Chen, H. Performance of Density Functionals for Activation Energies of Zr-Mediated Reactions. *J. Chem. Theory Comput.* **2013**, *9* (11), 4735–4743. https://doi.org/10.1021/CT400432X/SUPPL_FILE/CT400432X_SI_001.PDF.

(14) Scalmani, G.; Frisch, M. J. Continuous Surface Charge Polarizable Continuum Models of Solvation. I. General Formalism. *J. Chem. Phys.* **2010**, *132* (11), 114110. https://doi.org/10.1063/1.3359469.

(15) York, D. M. A Smooth Solvation Potential Based on the Conductor-like Screening Model. *J. Phys. Chem. A* **1999**, *103* (50), 11040–11044. https://doi.org/10.1021/JP992097L.

(16) Liu, B.; Roisnel, T.; Maron, L.; Carpentier, J. F.; Sarazin, Y. Discrete Divalent Rare-Earth Cationic ROP Catalysts: Ligand-Dependent Redox Behavior and Discrepancies with Alkaline-Earth Analogues in a Ligand-Assisted Activated Monomer Mechanism. *Chem. - A Eur. J.* **2013**, *19* (12), 3986–3994. https://doi.org/10.1002/chem.201204340.

(17) Andrew P. Dove; Vernon C. Gibson, *; Edward L. Marshall; Henry S. Rzepa; Andrew J. P. White, A.; Williams, D. J. Synthetic, Structural, Mechanistic, and Computational Studies on Single-Site β-Diketiminate Tin(II) Initiators for the Polymerization of Rac-Lactide. *J. Am. Chem. Soc.* **2006**, *128*, 9834–9843. https://doi.org/10.1021/JA061400A.

(18) Marshall, E. L.; Gibson, V. C.; Rzepa, H. S. A Computational Analysis of the Ring-Opening Polymerization of Rac-Lactide Initiated by Single-Site β-Diketiminate Metal Complexes: Defining the Mechanistic Pathway and the Origin of Stereocontrol. *J. Am. Chem. Soc.* **2005**, *127* (16), 6048–6051. https://doi.org/10.1021/ja043819b.

(19) Dyer, H. E.; Huijser, S.; Susperregui, N.; Bonnet, F.; Schwarz, A. D.; Duchateau, R.; Maron, L.; Mountford, P. Ring-Opening Polymerization of Rac-Lactide by Bis(Phenolate)Amine-Supported Samarium Borohydride Complexes: An Experimental and DFT Study. *Organometallics* **2010**, *29* (16), 3602–3621. https://doi.org/10.1021/om100513j.
